# Kookaburra Optimization Algorithm: A New Bio-Inspired Metaheuristic Algorithm for Solving Optimization Problems

**DOI:** 10.3390/biomimetics8060470

**Published:** 2023-10-01

**Authors:** Mohammad Dehghani, Zeinab Montazeri, Gulnara Bektemyssova, Om Parkash Malik, Gaurav Dhiman, Ayman E. M. Ahmed

**Affiliations:** 1 Department of Electrical and Electronics Engineering, Shiraz University of Technology, Shiraz 7155713876, Iran; z.montazeri@sutech.ac.ir; 2 Department of Computer Engineering, International Information Technology University, Almaty 050000, Kazakhstan; g.bektemisova@iitu.edu.kz; 3 Department of Electrical and Software Engineering, University of Calgary, Calgary, AB T2N 1N4, Canada; maliko@ucalgary.ca; 4 Department of Electrical and Computer Engineering, Lebanese American University, Byblos 13-5053, Lebanon; gdhiman0001@gmail.com; 5 University Centre for Research and Development, Department of Computer Science and Engineering, Chandigarh University, Mohali 140413, India; 6 Department of Computer Science and Engineering, Graphic Era Deemed to be University, Dehradun 248002, India; 7 Division of Research and Development, Lovely Professional University, Phagwara 144411, India; 8 Faculty of Computer Engineering, King Salman International University, El Tor 46511, Egypt; ayman.ahmed@ksiu.edu.eg

**Keywords:** optimization, bio-inspired, metaheuristic, kookaburra, exploration, exploitation

## Abstract

In this paper, a new bio-inspired metaheuristic algorithm named the Kookaburra Optimization Algorithm (KOA) is introduced, which imitates the natural behavior of kookaburras in nature. The fundamental inspiration of KOA is the strategy of kookaburras when hunting and killing prey. The KOA theory is stated, and its mathematical modeling is presented in the following two phases: (i) exploration based on the simulation of prey hunting and (ii) exploitation based on the simulation of kookaburras’ behavior in ensuring that their prey is killed. The performance of KOA has been evaluated on 29 standard benchmark functions from the CEC 2017 test suite for the different problem dimensions of 10, 30, 50, and 100. The optimization results show that the proposed KOA approach, by establishing a balance between exploration and exploitation, has good efficiency in managing the effective search process and providing suitable solutions for optimization problems. The results obtained using KOA have been compared with the performance of 12 well-known metaheuristic algorithms. The analysis of the simulation results shows that KOA, by providing better results in most of the benchmark functions, has provided superior performance in competition with the compared algorithms. In addition, the implementation of KOA on 22 constrained optimization problems from the CEC 2011 test suite, as well as 4 engineering design problems, shows that the proposed approach has acceptable and superior performance compared to competitor algorithms in handling real-world applications.

## 1. Introduction

There are many problems in science, engineering, mathematics, and real-world applications that have more than one feasible solution. These multi-solution problems are known as optimization problems. According to this definition, the process of determining the best feasible solution among all the available solutions for this type of problem is called optimization [1]. Optimization problems are mathematically modeled using three main parts: decision variables, constraints, and objective functions. The goal of optimization is to determine the optimal values for the decision variables in such a way that, respecting the constraints of the problem, the objective function becomes maximum or minimum [2]. Problem solving techniques that deal with optimization tasks are classified into the following two groups: deterministic and stochastic approaches [3]. Deterministic approaches, which are placed into two classes, gradient-based and non-gradient-based, have good performance in solving convex, linear, continuous, differentiable, and low-dimensional optimization problems [4]. Despite these advantages, deterministic approaches fail to solve complex, non-convex, nonlinear, discontinuous, non-differentiable, and high-dimensional optimization problems by getting stuck in inappropriate local solutions [5]. These are the characteristics and nature of many optimization problems in science, technology, industry, engineering, and real-world applications. Disadvantages and inabilities of deterministic approaches in handling such optimization problems have led researchers to develop stochastic approaches [6].

Metaheuristic algorithms are one of the most widely used stochastic approaches that are able to provide suitable solutions for optimization problems based on a random search in the problem-solving space and the use of random operators and trial-and-error processes. Moreover, there are many advantages, such as the following: independence from the type of problem, convenient implementation, easy concepts, no need for gradient information, efficiency in non-differentiable, discontinuous, complex, non-convex, nonlinear, and high-dimensional problems, and efficiency in nonlinear and unknown search spaces, has led to the popularity of metaheuristic algorithms among researchers. The optimization process in metaheuristic algorithms starts with the random generation of a number of feasible solutions. Then, during successive iterations of the algorithm and based on the update steps, these initial solutions are improved. After the completion of the iterations of the algorithm, the best solution obtained during the search process is presented as a solution to the problem [7]. Metaheuristic algorithms do not provide any guarantee to achieve the global optimal, and this is due to the random search nature of these approaches. However, given that the solutions obtained from metaheuristic algorithms for optimization problems are close to the global optimal, they are acceptable as quasi-optimal solutions. The desire of researchers to achieve better quasi-optimal solutions for optimization problems has led to the design of numerous metaheuristic algorithms [8]. These algorithms are employed to solve optimization problems in various sciences such as the following: structure design [9], simultaneous sound absorption and superior mechanical properties [10], energy [11,12,13], protection [14], electrical engineering [15,16,17,18,19], and energy carriers [20,21]. 

The two main pillars of the success of metaheuristic algorithms in providing an effective search process in the problem-solving space are exploration and exploitation. Exploration is the ability of the algorithm in the global search of the problem-solving space, with the aim of discovering the main optimal area and avoiding getting stuck in local optima. Exploitation is the ability of the algorithm in the local search of the problem-solving space, with the aim of obtaining possible better solutions near the promising areas and the obtained solutions. In addition to having exploration and exploitation, what leads to the successful performance of the metaheuristic algorithm in the optimization process is its ability to establish a balance between exploration and exploitation during the search process [22].

The main research question in the study of metaheuristic algorithms is that despite the many metaheuristic algorithms that have been designed so far, is there still a need to design new metaheuristic algorithms or not? In response to this challenge, the No Free Lunch (NFL) [23] theorem explains that the successful performance of a metaheuristic algorithm in solving a set of optimization problems is not a guarantee to provide the same performance of that algorithm in solving other optimization problems. In fact, a metaheuristic algorithm may provide an even global optimal in solving an optimization problem but fail in solving another optimization problem. According to the NFL theorem, there is no assumption about the success or failure of implementing a metaheuristic algorithm on an optimization problem. According to the NFL theorem, there is no unique metaheuristic algorithm that is the best optimizer for all optimization problems. By keeping the study field of metaheuristic algorithms active, the NFL theorem encourages and motivates researchers to be able to provide more effective solutions for optimization problems by introducing newer metaheuristic algorithms.

The novelty and innovation of this article is in the design of a new metaheuristic algorithm called the Kookaburra Optimization Algorithm (KOA), which is used in solving optimization problems. The scientific contributions of this study are as follows:

- KOA is designed based on mimicking the natural behavior of kookaburras in the wild;
- The fundamental inspiration of KOA is derived from (i) the kookaburras’ strategy during hunting and (ii) the behavior of kookaburras when they slam their prey into a tree to ensure that the prey is killed;
- The implementation steps of KOA are described and mathematically modeled in two phases of exploration and exploitation based on simulating the behavior of kookaburras in nature;
- The effectiveness of KOA in solving optimization problems has been evaluated in the CEC 2017 test suite;
- The performance of KOA in handling real-world applications has been tested on 22 constrained optimization problems from the CEC 2011 test suite as well as 4 engineering design problems;
- The results of KOA have been compared with the performance of 12 well-known metaheuristic algorithms.

The structure of the paper is as follows: the literature review is presented in Section 2. Then the proposed Kookaburra Optimization Algorithm (KOA) is introduced and modeled in Section 3. The simulation studies and results are presented in Section 4. The effectiveness of KOA in solving real-world applications is investigated in Section 5. The conclusions and suggestions for future research are provided in Section 6.

## 2. Literature Review

Metaheuristic algorithms have been developed with inspiration from various natural phenomena, natural behaviors of living organisms in nature, laws of physics, biological concepts, game rules, human behaviors, and other evolutionary phenomena. Based on the main design idea, metaheuristic algorithms are placed in the following five groups: swarm-based, evolutionary-based, physics-based, human-based, and game-based approaches.

Swarm-based metaheuristic algorithms are designed inspired by the swarming phenomena among animals, insects, reptiles, aquatic, birds, and other living organisms. Ant Colony Optimization (ACO) [24], Artificial Bee Colony (ABC) [25], Particle Swarm Optimization (PSO) [26], and Firefly Algorithm (FA) [27] are among the most prominent swarm-based metaheuristic algorithms that have been employed in many optimization applications. ACO is designed inspired by the ant colony’s ability to identify the shortest communication path between the nest and the food source. ABC is designed inspired by the activities and interactions of honeybees in the colony to access food resources. PSO is developed inspired by the swarming movement of flocks of fish and birds searching for food sources. FA is introduced inspired by the exchange and communication of information between fireflies using optical communication. Among the natural swarming behaviors in wildlife, foraging, hunting, chasing, and migration are more prominent and have been sources of inspiration in the design of several metaheuristic algorithms such as: Green Anaconda Optimization (GAO) [28], Coati Optimization Algorithm (COA) [29], Pelican Optimization Algorithm (POA) [30], African Vultures Optimization Algorithm (AVOA) [31], White Shark Optimizer (WSO) [32], Orca Predation Algorithm (OPA) [33], Grey Wolf Optimizer (GWO) [34], Serval Optimization Algorithm (SOA) [35], Marine Predator Algorithm (MPA) [36], Subtraction-Average-Based Optimizer (SABO) [37], Whale Optimization Algorithm (WOA) [38], Golden Jackal Optimization (GJO) [39], Tunicate Swarm Algorithm (TSA) [40], Honey Badger Algorithm (HBA) [41], and Reptile Search Algorithm (RSA) [42].

Evolutionary-based metaheuristic algorithms are designed with inspiration from genetics and biology sciences, the concepts of natural selection and evolutionary operators. Genetic algorithm (GA) [43] and differential evolution (DE) [44] are the most familiar names of evolutionary-based metaheuristic algorithms that have been widely used in handling optimization tasks. GA and DE are developed with inspiration from the process of reproduction, Darwin’s theory of evolution, survival of the fittest, and random genetic operators such as mutation, crossover, and selection. Artificial immune systems (AISs) are designed inspired by the body’s defense and immunity mechanisms against diseases and microbes [45]. Some other evolutionary-based metaheuristic algorithms are as follows: evolution strategy (ES) [46], cultural algorithm (CA) [47], and genetic programming (GP) [48].

Physics-based metaheuristic algorithms are designed with inspiration from forces, laws, concepts, phenomena, and transformations in physics. Simulated annealing (SA) [49] is one of the most widely used physics-based metaheuristic algorithms, which is designed with the inspiration of the metal annealing process where, in order to achieve an ideal crystal, the metal is first melted under heat and then slowly cooled. Physical forces and Newton’s laws of motion are employed in designing algorithms such as the following: Momentum Search Algorithm (MSA) [50] based on momentum force, Spring Search Algorithm (SSA) [51] based on spring force, and Gravitational Search Algorithm (GSA) [52] based on gravitational force. Various physical transformations during the natural water cycle have been the main inspiration in the design of Water Cycle Algorithm (WCA) [53]. The concepts of cosmology have been fundamental in the development of algorithms such as Black Hole Algorithm (BHA) [54] and Multi-Verse Optimizer (MVO) [55]. Some other physics-based metaheuristic algorithms are as follows: Archimedes Optimization Algorithm (AOA) [56], Equilibrium Optimizer (EO) [57], Lichtenberg Algorithm (LA) [58], Thermal Exchange Optimization (TEO) [59], Electro-Magnetism Optimization (EMO) [60], Nuclear Reaction Optimization (NRO) [61], and Henry Gas Optimization (HGO) [62].

Human-based metaheuristic algorithms are designed with inspiration from different human strategies, such as interactions, communication, thoughts, decisions, and other human behaviors in personal and social life. Teaching–Learning Based Optimization (TLBO) is one of the most famous human-based metaheuristic algorithms, which is designed with the inspiration of educational relationships between students and teachers in the classroom [63]. The Mother Optimization Algorithm (MOA) is introduced based on Eshrat’s care of her children in the following three phases: education, advice, and upbringing [64]. The Teamwork Optimization Algorithm (TOA) is introduced with the inspiration of cooperation and interactions between teammates when providing a team work in order to achieve the set goals [65]. The Sewing Training-Based Optimization (STBO) is designed inspired by the process of learning sewing skills of students in sewing schools [66]. The Driving Training-Based Optimization (DTBO) is developed inspired by driving education and interactions between applicants and instructors in driving schools [5]. Some other human-based metaheuristic algorithms are as follows: Doctor and Patient Optimization (DPO) [67], Following Optimization Algorithm (FOA) [68], Ali Baba and the Forty Thieves (AFT) [69], Drawer Algorithm (DA) [70], Election-Based Optimization Algorithm (EBOA) [71], Chef-Based Optimization Algorithm (CHBO) [72], Coronavirus Herd Immunity Optimizer (CHIO) [73], War Strategy Optimization (WSO) [74], and Gaining Sharing Knowledge-Based Algorithm (GSK) [75].

Game-based metaheuristic algorithms are designed inspired by the governing rules, the behavior of players, coaches, referees, and other influential persons in various individual and team games. The Football Game-Based Optimization (FGBO) [76] and Volleyball Premier League (VPL) [77] are among the game-based metaheuristic algorithms developed based on the simulation of league matches between clubs. The effort of players to find a hidden object in the playground has been the main inspiration in the design of Puzzle Optimization Algorithm (POA) [78]. The effort of the players in the archery competition and shooting towards the scoreboard has been the main idea in the design of the Archery Algorithm (AA) [6]. Some other game-based metaheuristic algorithms are as follows: Darts Game Optimizer (DGO) [79], Golf Optimization Algorithm (GOA) [80], Dice Game Optimizer (DGO) [81], Orientation Search Algorithm (OSA) [82], Hide Object Game Optimizer (HOGO) [83], and Ring Toss Game-Based Optimization (RTGBO) [84].

Topology optimization-based methods are mathematical methods for optimizing the material distribution in a certain region based on the given performance metrics, constraints, and load conditions. Topology optimization has significant potential for practical engineering applications due to its greater design freedom compared to structural shape optimization and structural size optimization [85]. Topology optimization-based methods are employed in different applications such as the design of compliant robotic legs [86] and lightweight design [87].

Based on the best knowledge obtained from the literature review, no metaheuristic algorithm based on simulating the natural behavior of kookaburras has been designed so far. This is while the strategy of kookaburras when hunting and killing prey is an intelligent process that can be the basis for designing a new metaheuristic algorithm. With the aim of addressing this research gap in optimization studies, in this paper, a new meta-heuristic algorithm based on modeling the natural behavior of kookaburras in the wild is designed, which is discussed in the next section.

## 3. Kookaburra Optimization Algorithm

In this section, the inspiration source and theory of the proposed Kookaburra Optimization Algorithm (KOA) approach are stated, then its implementation steps are mathematically modeled in order to be used in solving optimization problems.

### 3.1. Inspiration of KOA

The Kookaburra of the Dacelo genus is a bird from the group of terrestrial tree kingfishers that lives on land, is carnivorous, and belongs to the Coraciiformes and Alcedininae families. This bird lives in the native habitats of New Guinea and Australia. They are found in habitats ranging from arid savannah to humid forest, as well as near running water or in suburban areas with tall trees. The sound of this bird is similar to human laughter, and with this sound, the bird basically warns its enemies not to approach its territory [88].

**Figure 1:**
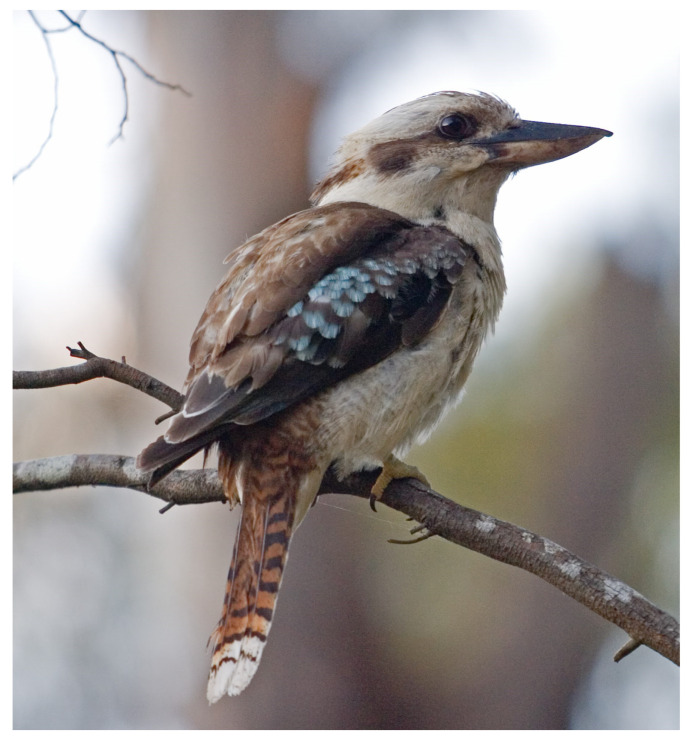
Kookaburra taken from: free media Wikimedia Commons.

Kookaburras can be found in different colors such as blue, brown, and white, and behind the eyes of this bird there is a dark brown spot, which gives the bird an angry awe along with the special shape of the feathers on its head. Kookaburra is between 28 and 47 cm long, and its weight is about 300 g [89]. A picture of a kookaburra is shown in Figure 1.

Kookaburras are carnivorous birds that feed on mice, insects, snakes, frogs, small reptiles, and small birds. The beak of the kookaburra is suitable for diving and hunting. The bird dives towards the prey with an open beak, and after hunting, it returns to the branch of the tree from which it flew from and knocks the prey against the tree several times to make sure it is dead. Then he holds the prey tightly between his claws, crushes it, and eats it [90].

Among the natural behaviors of the kookaburra in the wild, the strategy of this animal in hunting and knocking the prey against the tree in order to ensure that the prey is killed is much more significant. These natural kookaburra behaviors are the intelligent processes employed in the design of proposed KOA approach.

### 3.2. Algorithm Initialization

The proposed KOA approach is a population-based optimizer that is able to provide suitable solutions for optimization problems in an iterative-based process based on a random search in the problem-solving space. The KOA population consists of kookaburras that are placed in the problem-solving space so that each kookaburra determines values for the decision variables based on its position in the problem-solving space; therefore, each kookaburra is a candidate solution to the problem that can be modeled using a vector. Kookaburras together form the KOA population matrix, which can be modeled using a matrix according to Equation (1). The position of the kookaburras at the beginning of KOA implementation is randomly initialized using Equation (2).

(1) $$X={\left[\begin{array}{c} X_{1}\\ \vdots \\ X_{i}\\ \vdots \\ X_{N} \end{array}\right]}_{N\times m}={\left[\begin{array}{ccccc} x_{1,1} & \cdots & x_{1,d} & \cdots & x_{1,m}\\ \vdots & \ddots & \vdots & ⋰ & \vdots \\ x_{i,1} & \cdots & x_{i,d} & \cdots & x_{i,m}\\ \vdots & ⋰ & \vdots & \ddots & \vdots \\ x_{N,1} & \cdots & x_{N,d} & \cdots & x_{N,m} \end{array}\right]}_{N\times m}$$

(2) $$x_{i,d}=lb_{d}+r\cdot (ub_{d}-lb_{d})$$

Here, X is the KOA population matrix, $X_{i}$ is the ith kookaburra (candidate solution), $x_{i,d}$ is its dth dimension in search space (decision variable), N is the number of kookaburras, m is the number of decision variables, r is a random number in interval $\left[0,1\right]$, $lb_{d}$ and $ub_{d}$ are the lower bound and upper bound of the dth. decision variable, respectively.

Considering that the position of each kookaburra in the problem-solving space is a candidate solution for the problem corresponding to each kookaburra, the objective function of the problem can be evaluated. The set of evaluated values for the objective function of the problem can be represented using a vector according to Equation (3).

(3) $$F={\left[\begin{array}{c} F_{1}\\ \vdots \\ F_{i}\\ \vdots \\ F_{N} \end{array}\right]}_{N\times 1}={\left[\begin{array}{c} F(X_{1})\\ \vdots \\ F(X_{i})\\ \vdots \\ F(X_{N}) \end{array}\right]}_{N\times 1}$$

Here, F is the vector of evaluated objective function and $F_{i}$ is the evaluated objective function based on the ith kookaburra.

The evaluated values for the objective function are a suitable criterion for measuring the quality of candidate solutions and population members. The best evaluated value for the objective function corresponds to the best member and the worst evaluated value for the objective function corresponds to the worst member. Considering that in each iteration, the position of the kookaburras in the problem-solving space is updated, the objective function of the problem is reevaluated and based on the comparison of the new values, the best member of the population is also updated.

### 3.3. Mathematical Modelling of KOA

The proposed KOA approach updates the position of kookaburras in the following two phases: exploration and exploitation, in an iterative-based process in order to improve candidate solutions based on the simulation of natural kookaburra behaviors in the wild. Next, the process of updating the KOA population in the search space is presented.

#### 3.3.1. Phase 1: Hunting Strategy (Exploration)

The kookaburra is a carnivorous bird that feeds on other small birds, reptiles, insects, mice, frogs, etc. Although this bird has weak legs, they have a very strong neck that helps them in hunting. The strategy of kookaburras in selecting prey and attacking it leads to large displacement in their position. This process represents the global search with the concept of exploration, which refers to the detailed scanning of the problem-solving space with the aim of avoiding getting stuck in the local optimal in order to discover the main optimal area.

In order to simulate the hunting strategy of kookaburras, the position of other kookaburras, which have a better objective function value, is considered as the prey location in KOA design for each kookaburra. Therefore, based on the comparison of the objective function values, the available prey set for each kookaburra is determined using Equation (4).

(4) $$CP_{i}=\left\{X_{k}:F_{k}<F_{i}\;and\;k\neq i\right\},\;where\;i=1,2,\;\ldots ,\;N\;and\;k\in \left\{1,2,\;\ldots ,\;N\right\}$$

Here, $CP_{i}$ is the set of candidate prey for ith kookaburra, $X_{k}$ is the kookaburra with a better objective function value than the ith kookaburra, and $F_{k}$ is the objective function value.

In the KOA design, it is assumed that each kookaburra randomly selects a prey and attacks it. Based on the simulation of the movement of the kookaburra towards the prey in the hunting strategy, a new position for the kookaburra is calculated using Equation (5). In this case, if the value of the objective function is improved in the new position, this new position will replace the previous position of the corresponding kookaburra according to Equation (6).

(5) $$x_{i,d}^{P1}=x_{i,d}+r\cdot \left(SCP_{i,d}-I\cdot x_{i,d}\right),\;i=1,2,\;\ldots ,\;N,\;\;\;and\;d=1,2,\;\ldots ,m$$

(6) $$X_{i}=\left\{\begin{array}{cc} X_{i}^{P1},\; & F_{i}^{P1}<F_{i}\\ X_{i},\; & else \end{array}\right.$$

Here, $X_{i}^{P1}$ is the new suggested position of the *i*th kookaburra based on first phase of KOA, $x_{i,d}^{P1}$ is its dth dimension, $F_{i}^{P1}$ is its objective function value, r is a random number with a normal distribution in the range of $\left[0,1\right]$, $SCP_{i,d}$ is the dth dimension of selected prey for ith kookaburra, I is a random number from set $\left\{1,2\right\}$, N is the number of kookaburra, and m is the number of decision variables.

#### 3.3.2. Phase 2: Ensuring That the Prey Is Killed (Exploitation)

The second characteristic behavior of kookaburras is that after attacking the prey, the kookaburra carries the prey with itself and makes sure that the prey is killed by repeatedly hitting it against the tree. The kookaburra then holds the prey tightly between its claws and crushes and eats it. This behavior of kookaburras, which happens near the hunting ground, leads to small changes in their position. This process, which represents the local search with the concept of exploitation, refers to the ability of the algorithm to achieve better solutions near the obtained solutions and promising areas.

In the KOA design, in order to simulate this behavior of kookaburras based on their movement near the hunting place, a random position is calculated using Equation (7). In fact, it is assumed that this displacement occurs randomly in a neighborhood to the center of each kookaburra with a radius equal to $\frac{\left(ub_{d}\;-\;lb_{d}\right)}{t}$. The radius of this neighborhood is first set to the maximum value; then, during successive iterations, this radius becomes smaller so that the local search with the aim of converging towards better solutions can be performed more accurately. The new position calculated for each kookaburra replaces its previous position if it improves the value of the objective function according to Equation (8).

(7) $$x_{i,d}^{P2}=x_{i,d}+\left(1-2r\right)\cdot \frac{\left(ub_{d}-lb_{d}\right)}{t},\;i=1,2,\;\ldots ,\;N,\;\;d=1,2,\;\ldots ,m,\;\;\;and\;t=1,2,\;\ldots ,\;T$$

(8) $$X_{i}=\left\{\begin{array}{cc} X_{i}^{P2},\; & F_{i}^{P2}<F_{i}\\ X_{i},\; & \;\;else \end{array}\right.$$

Here, $X_{i}^{P2}$ is the new suggested position of the ith kookaburra based on the second phase of KOA, $x_{i,d}^{P2}$ is its dth dimension, $F_{i}^{P2}$ is its objective function value, t is the iteration counter of the algorithm, and T is the maximum number of algorithm iterations.

### 3.4. Repetition Process, Pseudocode, and Flowchart of KOA

**Figure 2:**
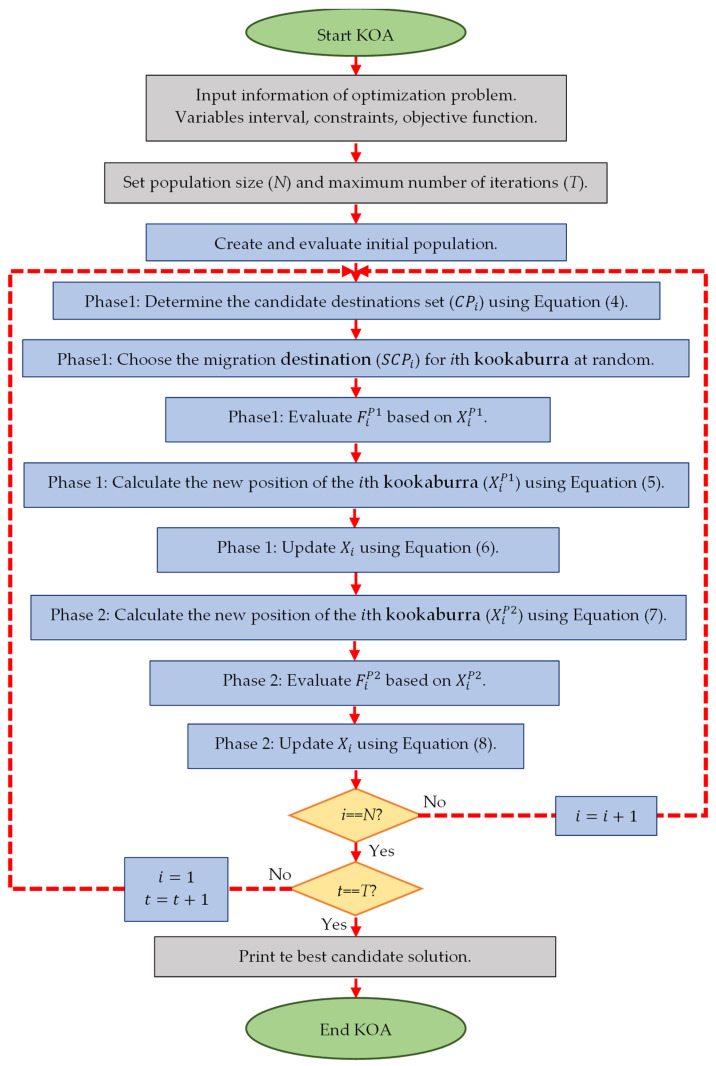
Flowchart of KOA.

The first iteration of KOA is completed after updating the location of all kookaburras based on the first and second phases. At the end of each iteration, the best solution obtained until that iteration is updated and saved. Then, based on the updated positions and the new evaluated values for the objective function, the algorithm enters the next iteration. The process of updating the position of kookaburras continues until the last iteration of the algorithm based on Equations (4)–(8). In the end, the best candidate solution obtained during the iterations of the algorithm is presented as the proposed solution by KOA for the problem. The steps of KOA implementation are presented as a flowchart in Figure 2, and its pseudo code is presented in Algorithm 1.
**Algorithm 1** Pseudocode of KOAStart KOA.1.Input problem information: variables, objective function, and constraints.2.Set KOA population size (*N*) and iterations (*T*).3.Generate the initial population matrix at random using Equation (2). $x_{i,d}\leftarrow lb_{d}+r\cdot (ub_{d}-lb_{d})$4.Evaluate the objective function.5.For t=1 to *T*6.For  i=1 to N
7.Phase 1: hunting strategy (exploration)8.Determine the candidate preys set using Equation (4). $CP_{i}\leftarrow \left\{X_{k_{i}}:F_{k_{i}}<F_{i}\;and\;k_{i}\neq i\right\}$
9.Choose the prey for the *i*th KOA member at random.10.Calculate new position of *i*th KOA member using Equation (5). $x_{i,d}^{P1}\leftarrow x_{i,d}+r\cdot \left(SCP_{i,d}-I\cdot x_{i,d}\right)$
11.Update *i*th KOA member using Equation (6). $X_{i}=\left\{\begin{array}{cc} X_{i}^{P1},\; & F_{i}^{P1}<F_{i}\\ X_{i},\; & \;\;else \end{array}\right.$
12.Phase 2: Ensuring that the prey is killed (exploitation)13.Calculate new position of *i*th KOA member using Equation (7). $x_{i,d}^{P2}\leftarrow x_{i,d}+(1-2r)\cdot \frac{\left(ub_{d}\;-\;lb_{d}\right)}{t}$
14.Update *i*th KOA member using Equation (8). $X_{i}=\left\{\begin{array}{cc} X_{i}^{P2},\; & F_{i}^{P2}<F_{i}\\ X_{i},\; & \;\;else \end{array}\right.$
15.end16.Save the best candidate solution so far.17.end 18. Output the best quasi-optimal solution obtained with the KOA.End KOA.

### 3.5. Computational Complexity of KOA

In this subsection, the analysis of the computational complexity of KOA is discussed. The KOA initialization steps have a complexity equal to *O(Nm)*, where *N* is the number of kookaburras and *m* is the number of decision variables of the problem. In each iteration of KOA, the position of each kookaburra in the problem-solving space is updated in the two phases of exploration and exploitation. Therefore, the process of updating kookaburras has a complexity equal to *O(2NmT)*, where *T* is the maximum number of iterations of the algorithm. Therefore, the total computational complexity of the proposed KOA approach is equal to *O(Nm(1 + 2T))*.

## 4. Simulation Studies and Results

In this section, simulation studies are presented on the performance of KOA in dealing with optimization scenarios. The performance of KOA has been evaluated on 29 standard benchmark functions from the Competitions on Evolutionary Computation (CEC) 2017 test suite for problem dimensions equal to 10, 30, 50, and 100. In order to measure the performance quality of KOA, the obtained results have been compared with the performance of the following 12 well-known metaheuristic algorithms: GA [43], PSO [26], GSA [52], TLBO [63], MVO [55], GWO [34], WOA [38], MPA [36], TSA [40], RSA [42], AVOA [31], and WSO [32]. The control parameters of metaheuristic algorithms are specified in Table 1. The optimization results are reported using the following six statistical indicators: mean, best, worst, standard deviation (std), median, and rank. The ranking criterion of metaheuristic algorithms for each of the benchmark functions is the value of the mean index.

### 4.1. Evaluation CEC 2017 Test Suite

**Figure 3:**
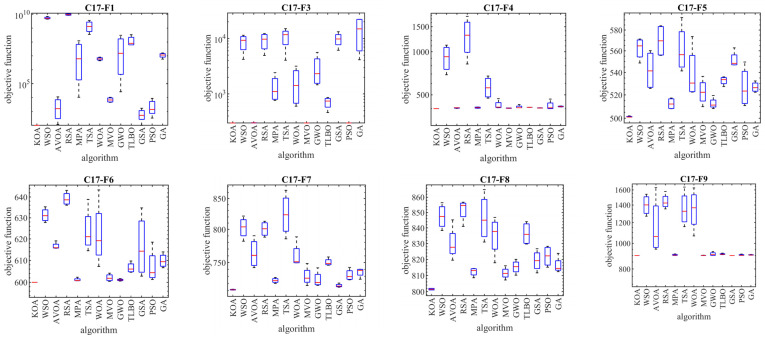

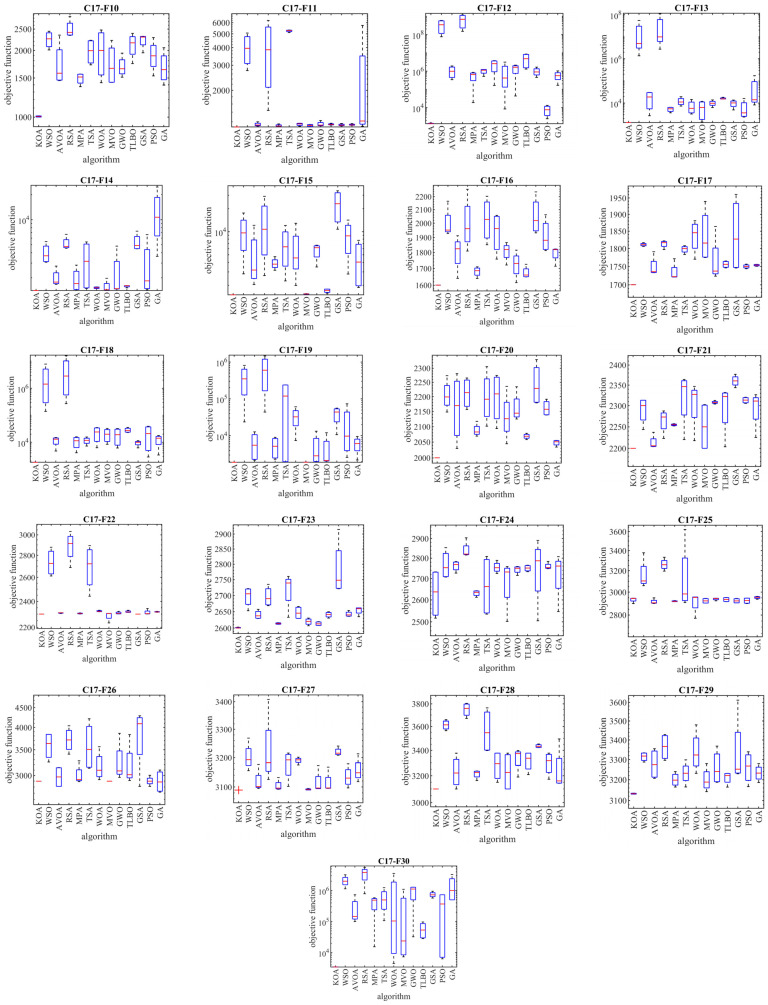
Boxplot diagrams of KOA and competitor algorithms performances on the CEC 2017 test suite (dimension = 10).

**Figure 4:**
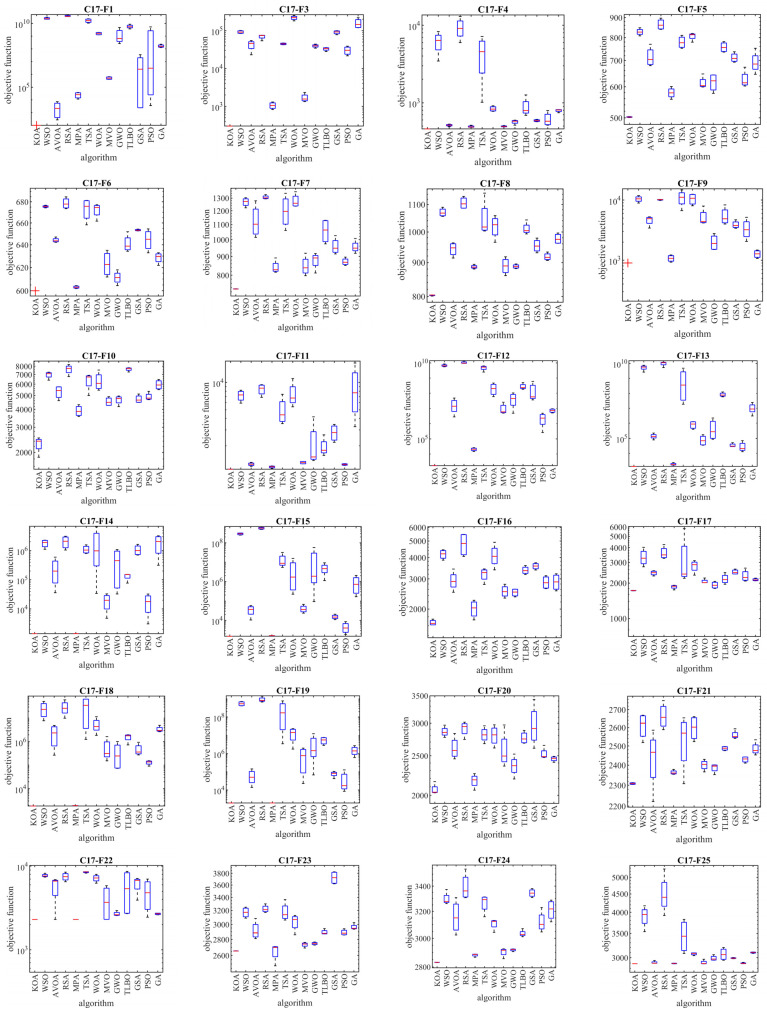

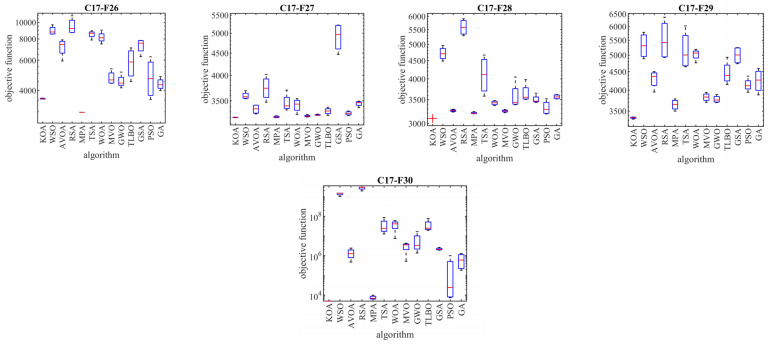
Boxplot diagrams of KOA and competitor algorithms performances on the CEC 2017 test suite (dimension = 30).

**Figure 5:**
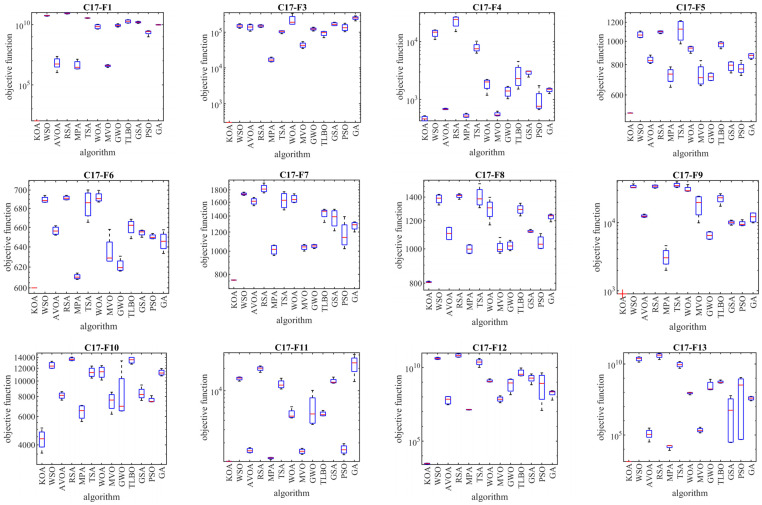

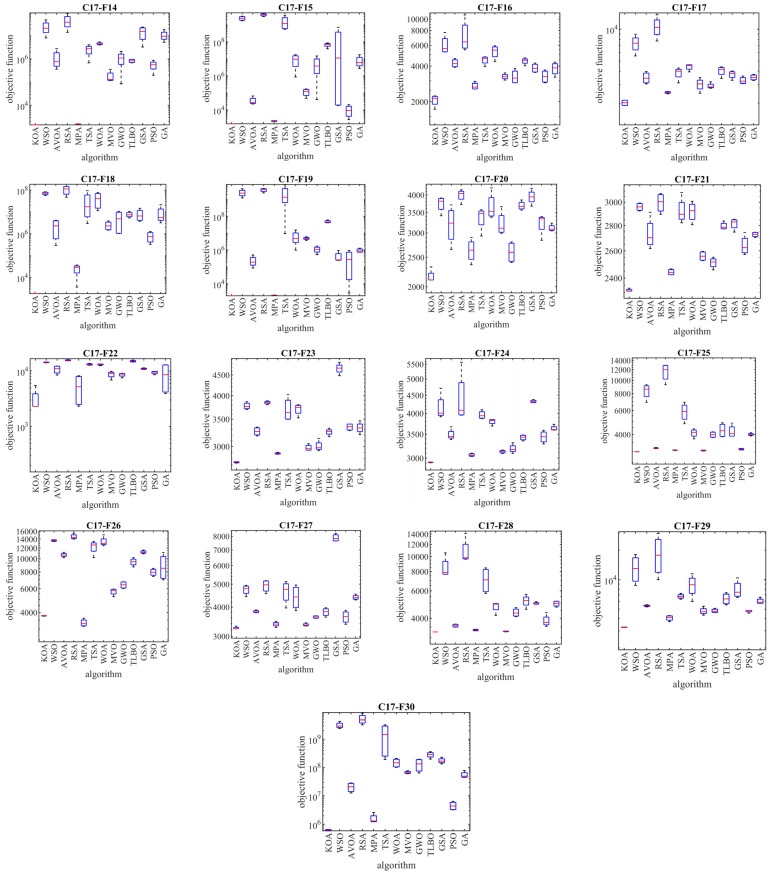
Boxplot diagrams of KOA and competitor algorithms performances on CEC 2017 test suite (dimension = 50).

**Figure 6:**
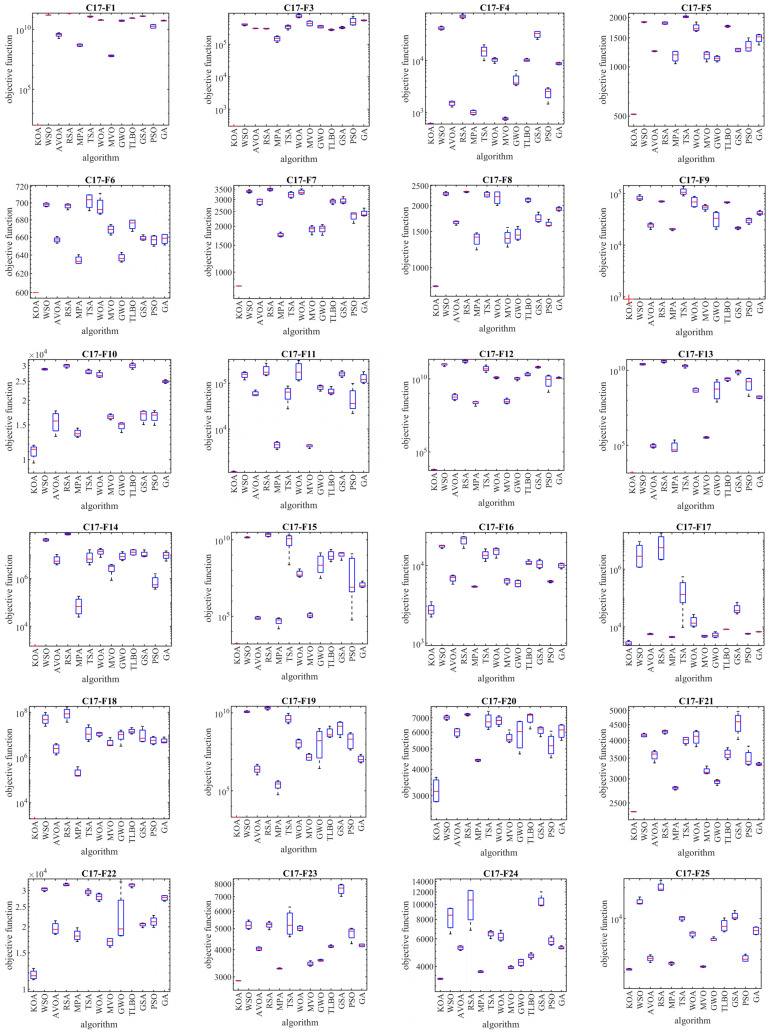

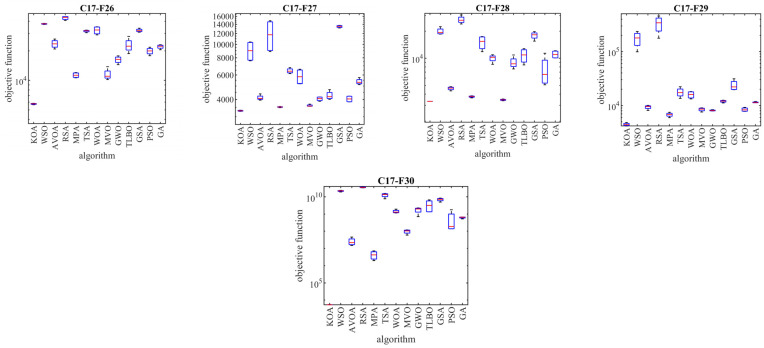
Boxplot diagrams of KOA and competitor algorithms performances on the CEC 2017 test suite (dimension = 100).

In this subsection, the evaluation of the proposed KOA approach in dealing with the Competitions on Evolutionary Computation (CEC) 2017 test suite is discussed. This test suite has 30 standard benchmark functions consisting of the following: 3 unimodal functions of C17-F1 to C17-F3, 7 multimodal functions of C17-F4 to C17-F10, 10 hybrid functions of C17-F11 to C17-F20, and 10 composition functions of C17-F21 to C17-F30. Among these, the C17-F2 function is not considered in the simulation studies due to the instability of the behavior. Information related to CEC 2017 test suite is provided in Appendix A and Table A1. The full description and details of the CEC 2017 test suite are provided in [91]. The implementation results of KOA and competitor algorithms on the CEC 2017 test suite for different dimensions of the problem equal to 10, 30, 50, and 100 are reported in Table 2, Table 3, Table 4 and Table 5. The boxplot diagrams resulting from the application of metaheuristic algorithms on the studied benchmark functions are drawn in Figure 3, Figure 4, Figure 5 and Figure 6. What is evident from the simulation results, in handling the CEC 2017 test suite for the problem dimension equal to 10, KOA is the first best optimizer for functions C17-F1, C17-F3 to C17-F24, and C17-F27 to C17-F30. For problem dimension equal to 30, KOA is the first best optimizer for functions C17-F1, C17-F3 to C17-F5, C17-F7, C17-F12 to C17-F14, C17-F16 to C17-F18, C17-F21 to C17-F27, and C17-F29. For problem dimension equal to 50, KOA is the first best optimizer for functions C17-F1, C17-F3 to C17-F25, and C17-F27 to C17-F30. For problem dimension equal to 100, KOA is the first best optimizer for functions C17-F1, and C17-F3 to C17-F30.

The optimization results show that the proposed KOA approach, with a high ability in both exploration and exploitation and the ability to balance them during the search process, has been able to provide suitable results for the benchmark functions. The analysis of the simulation results indicates that KOA, by providing better results and obtaining the rank of the first best optimizer in most of the benchmark functions, has provided a superior performance compared to competitor algorithms in addressing the CEC 2017 test suite in different dimensions of the problem equal to 10, 30, 50, and 100.

### 4.2. Statistical Analysis

In this subsection, using statistical analysis, it has been checked whether the superiority of KOA against competitor algorithms is significant from a statistical point of view or not. For this purpose, the Wilcoxon rank sum test [92] is used, which is a non-parametric test and is used to determine the significant difference between the average of two data samples. In this test, based on the values calculated for the *p*-value index, it is determined whether there is a statistically significant difference between the performance of the two algorithms or not. 

The results of statistical analysis on the performance of KOA and each of the competitor algorithms in handling the CEC 2017 test suite in different dimensions of the problem are reported in Table 6. Based on the results obtained from the Wilcoxon rank sum test, in cases where the *p*-value is less than 0.05, the proposed KOA approach has a statistically significant superiority in competition with the corresponding metaheuristic algorithm.

## 5. KOA for Real-World Applications

In this section, the efficiency of KOA in addressing real-world applications is challenged. For this purpose, 22 real-world constrained optimization problems from the CEC 2011 test suite as well as 4 classical engineering design problems are employed.

### 5.1. Evaluation CEC 2011 Test Suite

**Figure 7:**
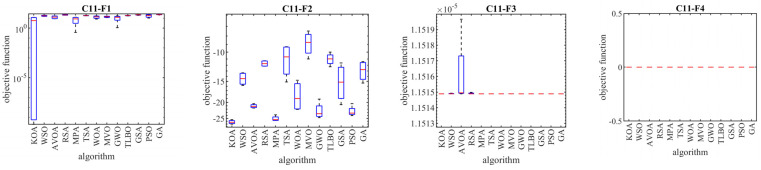

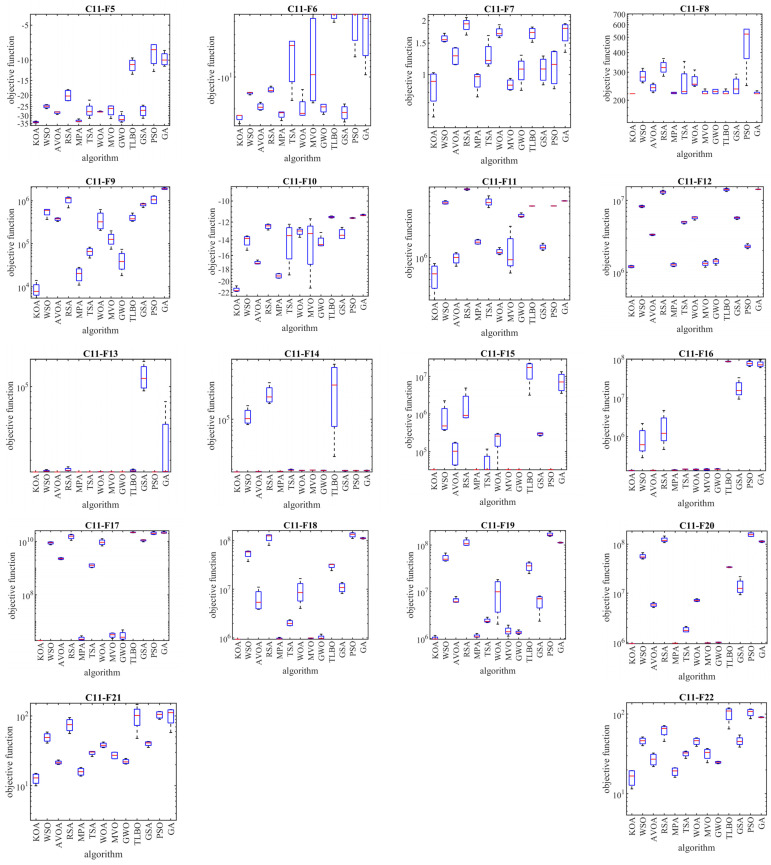
Boxplot diagrams of KOA and competitor algorithms performances on the CEC 2011 test suite.

In this subsection, the ability of KOA and competitor algorithms in handling the CEC 2011 test suite is evaluated. This test suite consists of 22 constrained optimization problems from real-world applications. The full description and details of CEC 2011 test suite are provided in [93]. The optimization results of CEC 2011 test suite using KOA and competitor algorithms are reported in Table 7. Also, the boxplot diagrams obtained from the performance of metaheuristic algorithms in solving optimization problems C11-F1 to C11-F22 are drawn in Figure 7.

The optimization results show that KOA, with its high ability to balance exploration and exploitation, has been able to provide suitable results for optimization problems in real-world applications. Based on the simulation results, KOA is the first best optimizer for C11-F1 to C11-F22. What is concluded from the analysis of the simulation results is that KOA has provided better results in most of the optimization problems compared to the competitor algorithms in handling the CEC 2011 test suite. Also, based on the statistical analysis and the results obtained from the Wilcoxon rank sum test, the superiority of KOA compared to competitor algorithms is significant from a statistical point of view.

### 5.2. Pressure Vessel Design Problem

**Figure 8:**
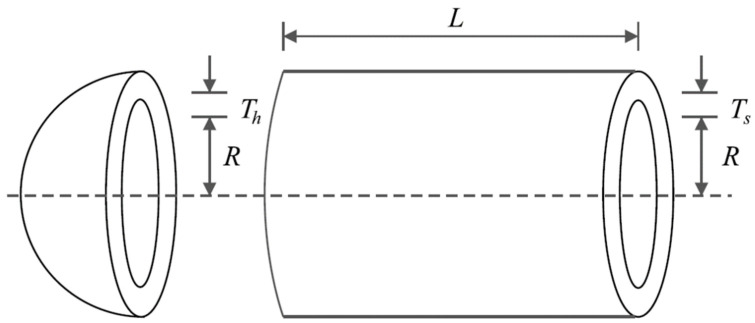
Schematic of pressure vessel design.

Pressure vessel design is a real-world optimization application aimed at minimizing construction cost. Pressure vessel design schematic is shown in Figure 8 and its mathematical model is as follows [94]:

Consider: $X=\left[x_{1},\;x_{2},\;x_{3},\;x_{4}\right]=\left[T_{s},\;T_{h},\;R,\;L\right]$.

Minimize: $f\left(x\right)=0.6224x_{1}x_{3}x_{4}+1.778x_{2}x_{3}^{2}+3.1661x_{1}^{2}x_{4}+19.84x_{1}^{2}x_{3}.$

Subject to:

$$g_{1}\left(x\right)=-x_{1}+0.0193x_{3}\;\leq \;0,\;\;g_{2}\left(x\right)=-x_{2}+0.00954x_{3}\leq \;0,$$

$$g_{3}\left(x\right)=-\pi x_{3}^{2}x_{4}-\frac{4}{3}\pi x_{3}^{3}+1,296,000\leq \;0,\;\;g_{4}\left(x\right)=x_{4}-240\;\leq \;0.$$

with

$$0\leq x_{1},x_{2}\leq 100\;{and\;10\leq x}_{3},x_{4}\leq 200.$$

**Figure 9:**
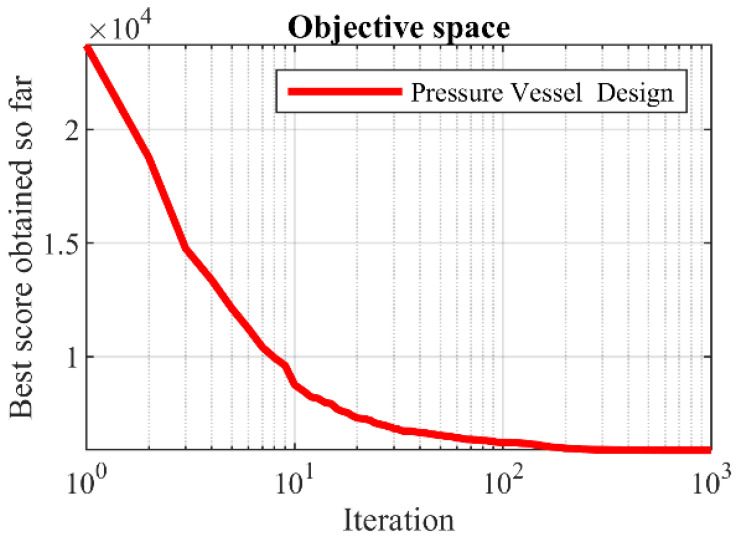
KOA’s performance convergence curve on pressure vessel design.

The pressure vessel design optimization results using KOA and competitor algorithms are reported in Table 8 and Table 9. Based on the results, KOA has provided the optimal design with the values of design variables equal to (0.7780271, 0.3845792, 40.312284, 200) and the corresponding objective function value equal to (5882.8955). The convergence curve of KOA during the pressure vessel design optimization is drawn in Figure 9. Based on the comparison of optimization results, it is evident that KOA has provided superior performance in the pressure vessel design optimization compared to the competitor algorithms.

### 5.3. Speed Reducer Design Problem

**Figure 10:**
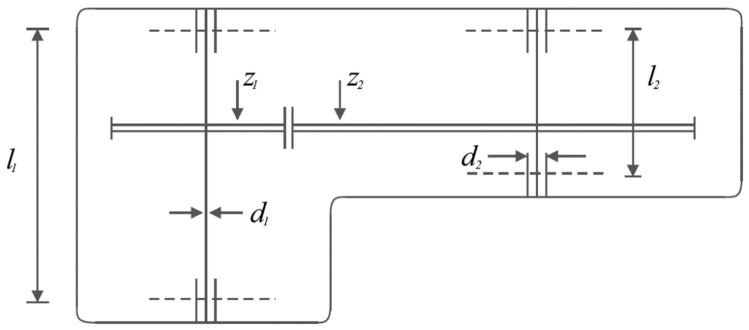
Schematic of the speed reducer design.

Speed reducer design is an engineering challenge with the aim of minimizing the weight of the speed reducer. The schematic of speed reducer design is shown in Figure 10 and its mathematical model is as follows [95,96]:

Consider: $X=\left[x_{1,}x_{2},x_{3},x_{4},x_{5}{,x}_{6},x_{7}\right]=\left[b,m,p,l_{1},l_{2},d_{1},d_{2}\right]$.

Minimize: $f\left(x\right)=0.7854x_{1}x_{2}^{2}\left(3.3333x_{3}^{2}+14.9334x_{3}-43.0934\right)-1.508x_{1}\left(x_{6}^{2}+x_{7}^{2}\right)+7.4777\left(x_{6}^{3}+x_{7}^{3}\right)+0.7854(x_{4}x_{6}^{2}+x_{5}x_{7}^{2})$.

Subject to:

$$g_{1}\left(x\right)=\frac{27}{x_{1}x_{2}^{2}x_{3}}-1\leq 0,\;g_{2}\left(x\right)=\frac{397.5}{x_{1}x_{2}^{2}x_{3}}-1\leq 0,$$

$$g_{3}\left(x\right)=\frac{1.93x_{4}^{3}}{x_{2}x_{3}x_{6}^{4}}-1\leq 0,\;g_{4}\left(x\right)=\frac{1.93x_{5}^{3}}{x_{2}x_{3}x_{7}^{4}}-1\leq 0,$$

$$g_{5}\left(x\right)=\frac{1}{110x_{6}^{3}}\sqrt{{\left(\frac{745x_{4}}{x_{2}x_{3}}\right)}^{2}+16.9\times 10^{6}}-1\leq 0,$$

$$g_{6}(x)=\frac{1}{85x_{7}^{3}}\sqrt{{\left(\frac{745x_{5}}{x_{2}x_{3}}\right)}^{2}+157.5\times 10^{6}}-1\leq 0,$$

$$g_{7}\left(x\right)=\frac{x_{2}x_{3}}{40}-1\leq 0,\;g_{8}\left(x\right)=\frac{{5x}_{2}}{x_{1}}-1\leq 0,$$

$$g_{9}\left(x\right)=\frac{x_{1}}{12x_{2}}-1\leq 0,\;g_{10}\left(x\right)=\frac{{1.5x}_{6}+1.9}{x_{4}}-1\leq 0,$$

$$g_{11}\left(x\right)=\frac{{1.1x}_{7}+1.9}{x_{5}}-1\leq 0.$$

with

$$2.6\leq x_{1}\leq 3.6,\;0.7\leq x_{2}\leq 0.8,\;17\leq x_{3}\leq 28,\;7.3\leq x_{4}\leq 8.3,\;7.8\leq x_{5}\;\leq 8.3,\;2.9\leq x_{6}\leq 3.9,\;and\;5\leq x_{7}\leq 5.5.$$

**Figure 11:**
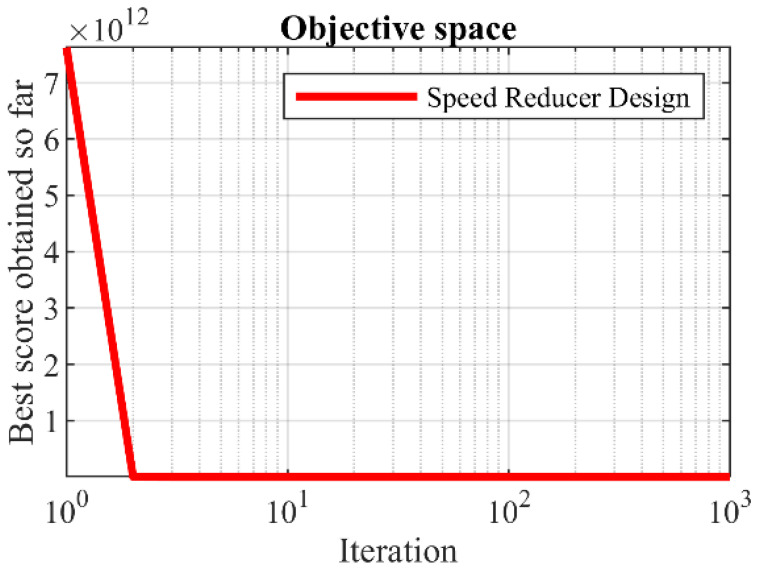
KOA’s performance convergence curve on speed reducer design.

The implementation results of KOA and competitor algorithms on the speed reducer design are presented in Table 10 and Table 11. Based on the results, KOA has provided the optimal design with the values of design variables equal to (3.5, 0.7, 17, 7.3, 7.8, 3.3502147, 5.2866832) and the corresponding objective function value equal to (2996.3482). The convergence curve of KOA while achieving the optimal design for the speed reducer design problem is drawn in Figure 11. The analysis of the simulation results shows the superiority of KOA performance compared to the competitor algorithms in order to handle the speed reducer design.

### 5.4. Welded Beam Design

**Figure 12:**
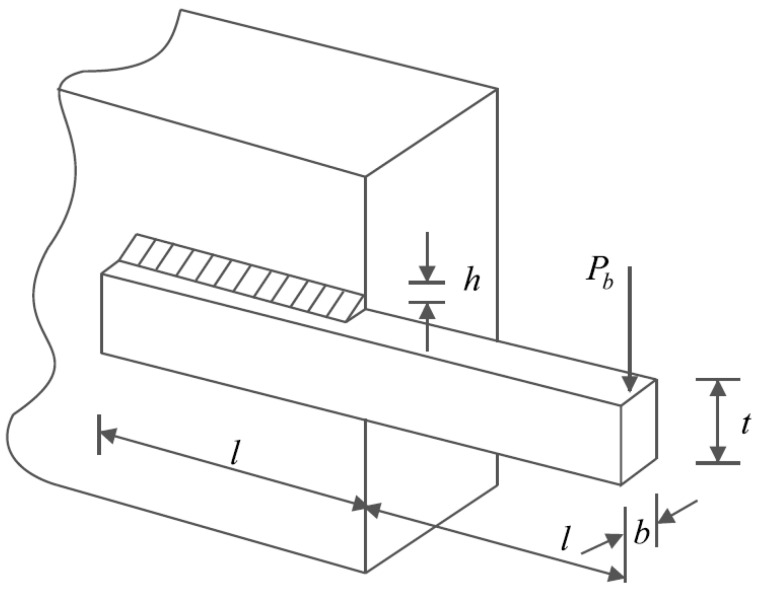
Schematic of welded beam design.

Welded beam design is a real-world application in engineering with the aim of minimizing the fabrication cost of the welded beam. The schematic of welded beam design is shown in Figure 12 and its mathematical model is as follows [38]:

Consider: $X=\left[x_{1},\;x_{2},\;x_{3},\;x_{4}\right]=\left[h,\;l,\;t,\;b\right]$.

Minimize: $f(x)=1.10471x_{1}^{2}x_{2}+0.04811x_{3}x_{4}\;(14.0+x_{2})$.

Subject to:

$$g_{1}\left(x\right)=\tau \left(x\right)-13,600\;\leq \;0,\;g_{2}\left(x\right)=\sigma \left(x\right)-30,000\;\leq \;0,$$

$$g_{3}\left(x\right)=x_{1}-x_{4}\leq \;0,\;g_{4}(x)=0.10471x_{1}^{2}+0.04811x_{3}x_{4}\;(14+x_{2})-5.0\;\leq \;0,$$

$$g_{5}\left(x\right)=0.125-x_{1}\leq \;0,\;g_{6}\left(x\right)=\delta \;\left(x\right)-0.25\;\leq \;0,$$

$$g_{7}\left(x\right)=6000-p_{c}\;\left(x\right)\leq \;0.$$

where

$$\tau \left(x\right)=\sqrt{{\left(\tau^{\prime }\right)}^{2}+\left(2\tau \tau^{\prime }\right)\frac{x_{2}}{2R}+{\left(\tau ”\right)}^{2}\;},\;\tau^{\prime }=\frac{6000}{\sqrt{2}x_{1}x_{2}},\;\tau ”=\frac{MR}{J},$$

$$M=6000\left(14+\frac{x_{2}}{2}\right),\;R=\sqrt{\frac{x_{2}^{2}}{4}+{\left(\frac{x_{1}+x_{3}}{2}\right)}^{2}},$$

$$J=2\left\{x_{1}x_{2}\sqrt{2}\left[\frac{x_{2}^{2}}{12}+{\left(\frac{x_{1}+x_{3}}{2}\right)}^{2}\right]\right\}\;,\;\sigma \left(x\right)=\frac{504,000}{x_{4}x_{3}^{2}}\;,$$

$$\delta \;\left(x\right)=\frac{65,856,000}{\left(30\cdot 10^{6}\right)x_{4}x_{3}^{3}}\;,\;p_{c}\;\left(x\right)=\frac{4.013\left(30\cdot 10^{6}\right)\sqrt{\frac{x_{3}^{2}x_{4}^{6}}{36}}}{196}\left(1-\frac{x_{3}}{28}\sqrt{\frac{30\cdot 10^{6}}{4(12\cdot 10^{6})}}\right)\;.$$

with

$$0.1\leq x_{1},\;x_{4}\leq 2\;and\;0.1\leq x_{2},\;x_{3}\leq 10.$$

**Figure 13:**
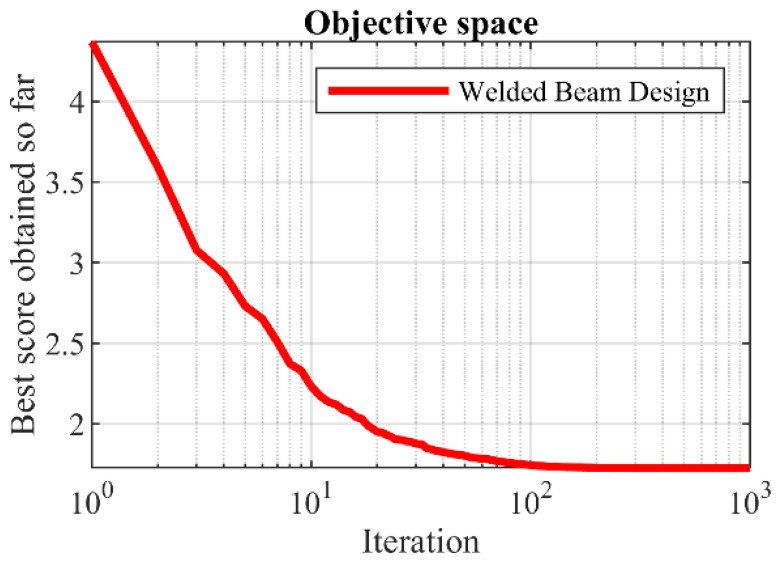
KOA’s performance convergence curve on welded beam design.

The results of employing KOA and competitor algorithms in handling the welded beam design problem are reported in Table 12 and Table 13. Based on the results, KOA has provided the optimal design with the values of design variables equal to (0.2057296, 3.4704887, 9.0366239, 0.2057296) and the corresponding objective function value equal to (1.7246798). The convergence process of KOA towards the optimal solution for the welded beam design problem is drawn in Figure 13. What is clear from the analysis of the optimization results is that KOA has provided a more effective performance compared to the competitor algorithms in the optimization of the welded beam design.

### 5.5. Tension/Compression Spring Design 

**Figure 14:**
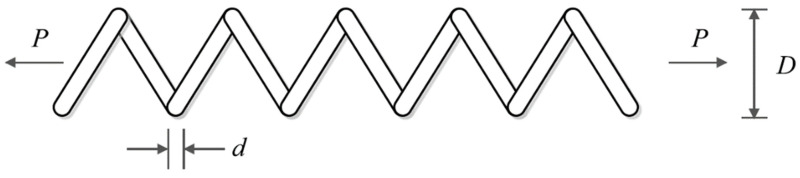
Schematic of tension/compression spring design.

Tension/compression spring design is an engineering subject of real-world applications with the aim of minimizing the weight of tension/compression spring. The schematic of welded beam design is shown in Figure 14 and its mathematical model is as follows [38]:

Consider: $X=\left[x_{1},\;x_{2},\;x_{3}\right]=\left[d,\;D,\;P\right].$

Minimize: $f\left(x\right)=\left(x_{3}+2\right)x_{2}x_{1}^{2}.$

Subject to:

$$g_{1}\left(x\right)=1-\frac{x_{2}^{3}x_{3}}{71,785x_{1}^{4}}\;\leq \;0,\;g_{2}\left(x\right)=\frac{4x_{2}^{2}-x_{1}x_{2}}{12,566(x_{2}x_{1}^{3})}+\frac{1}{5108x_{1}^{2}}-1\leq \;0,$$

$$g_{3}\left(x\right)=1-\frac{140.45x_{1}}{x_{2}^{2}x_{3}}\leq \;0,\;\;g_{4}\left(x\right)=\frac{x_{1}+x_{2}}{1.5}-1\;\leq \;0.$$

with

$$0.05\leq x_{1}\leq 2,\;{0.25\leq x}_{2}\leq 1.3\;and\;2\leq \;x_{3}\leq 15.$$

**Figure 15:**
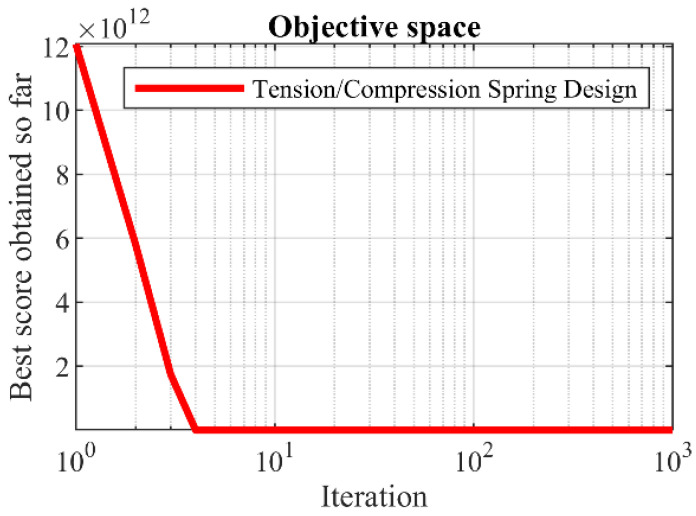
KOA’s performance convergence curve on tension/compression spring.

The optimization results of tension/compression spring design using KOA and competitor algorithms are reported in Table 14 and Table 15. Based on the results, KOA has provided the optimal design with the values of design variables equal to (0.0516891, 0.3567177, 11.288966) and the corresponding objective function value equal to (0.0126019). The convergence curve of KOA to the optimal solution for the tension/compression spring design problem is plotted in Figure 15. The analysis of the simulation results shows that KOA has provided superior performance in dealing with tension/compression spring design by providing better results compared to the competitor algorithms.

## 6. Conclusions and Future Works

In this paper, a new metaheuristic algorithm named the Kookaburra Optimization Algorithm (KOA) was introduced, which has applications in dealing with optimization issues. The fundamental inspiration for KOA is derived from the strategy of kookaburras when hunting and their behavior to ensure that the prey is killed. The theory of KOA was stated and mathematically modeled in the two phases of exploration and exploitation, which are based on simulating the natural behaviors of kookaburras. The effectiveness of the proposed KOA approach in handling optimization tasks was evaluated on the CEC 2017 test suite for problem dimensions equal to 10, 30, 50, and 100. The optimization results showed that KOA, with its high ability in exploration, exploitation, and establishing a balance between them, has been able to provide suitable solutions for the benchmark functions. The quality of KOA in the optimization process was compared with the performance of the 12 well-known metaheuristic algorithms. Based on the simulation results, by achieving better results for most benchmark functions, KOA provided superior performance compared to competitor algorithms in the handling of the CEC 2017 test suite. Also, the implementation of KOA on 22 constrained optimization problems from the CEC 2011 test suite, as well as 4 engineering design problems, indicated the capability of the proposed approach in addressing real-world applications.

By introducing the proposed approach of KOA, several research tasks are proposed for further studies. Designing binary and multi-purpose versions of KOA is one of the most special research proposals of this study. Also, using KOA to solve optimization problems in different sciences and real-world applications are other research proposals for future studies.

## Figures and Tables

**Table 1:** Control parameters values.

Algorithm	Parameter	Value
GA	Type	Real coded
	Selection	Roulette wheel (Proportionate)
	Crossover	Whole arithmetic (Probability = 0.8, $\alpha \in \left[-0.5,1.5\right]$)
	Mutation	Gaussian (Probability = 0.05)
PSO	Topology	Fully connected
	Cognitive and social constant	(*C*_1_, *C*_2_)=(2, 2)
	Inertia weight	Linear reduction from 0.9 to 0.1
	Velocity limit	10% of dimension range
GSA	Alpha, *G*_0_, *R_norm_*, *R_power_*	20, 100, 2, 1
TLBO	*T_F_*: teaching factor	*T_F_* = round $\left[\left(1+rand\right)\right]$
	Random number	*rand* is a random number between [0–1].
GWO	Convergence parameter (*a*)	*a*: Linear reduction from 2 to 0.
MVO	Wormhole existence probability (WEP)	Min(WEP) = 0.2 and Max(WEP) = 1.
	Exploitation accuracy over the iterations (*p*)	p=6.
WOA	Convergence parameter (*a*)	*a*: Linear reduction from 2 to 0.
	*r* is a random vector in $\left[0–1\right].$	
	*l* is a random number in $\left[-1,1\right].$	
TSA		
	P_min_ and P_max_	1, 4
	*c*1, *c*2, *c*3	Random numbers lie in the range of $\left[0–1\right].$
MPA	Constant number	*p* = 0.5
	Random vector	*R* is a vector of uniform random numbers in $\left[0,1\right].$
	Fish Aggregating Devices (*FADs*)	*FADs* = 0.2
	Binary vector	*U* = 0 or 1
RSA	Sensitive parameter	β=0.01
	Sensitive parameter	α=0.1
	Evolutionary Sense (ES)	ES: randomly decreasing values between 2 and −2
AVOA	L_1_, L_2_	0.8, 0.2
	w	2.5
	P_1_, P_2_, P_3_	0.6, 0.4, 0.6
WSO	F_min_ and F_max_	0.07, 0.75
	*τ*, *a*_0_ , *a*_1_ , *a*_2_	4.125, 6.25, 100, 0.0005

**Table 2:** Optimization results of CEC 2017 test suite (dimension = 10).

		KOA	WSO	AVOA	RSA	MPA	TSA	WOA	MVO	GWO	TLBO	GSA	PSO	GA
C17-F1	mean	100	5.42 × 10^9^	3646.942	9.67 × 10^9^	33,430,909	1.65 × 10^9^	6,111,054	7131.038	83,576,400	1.39 × 10^8^	712.6012	2984.583	11,229,309
	best	100	4.46 × 10^9^	114.7977	8.36 × 10^9^	10,619.9	3.53 × 10^8^	4,449,740	4537.763	26,341.55	62,120,924	100.0182	332.7586	5,814,967
	worst	100	7.02 × 10^9^	11,292.36	1.15 × 10^10^	1.21 × 10^8^	3.59 × 10^9^	8,045,954	10,505.13	3.04 × 10^8^	3.36 × 10^8^	1701.327	8827.164	16,120,640
	std	0	1.2 × 10^9^	5643.075	1.54 × 10^9^	63,706,411	1.56 × 10^9^	1,644,930	3021.388	1.59 × 10^8^	1.43 × 10^8^	748.8068	4251.125	4,655,595
	median	100	5.09 × 10^9^	1590.305	9.41 × 10^9^	6,127,683	1.32 × 10^9^	5,974,262	6740.631	15,317,772	79,652,751	524.5298	1389.204	11,490,815
	rank	1	12	4	13	8	11	6	5	9	10	2	3	7
C17-F3	mean	300	8545.047	301.7937	9154.734	1349.094	10627.2	1654.399	300.0517	2922.734	703.7751	9732.523	300	14,009.65
	best	300	4204.342	300	4943.483	765.3836	4056.697	602.4388	300.012	1463.459	462.1985	6130.294	300	4135.906
	worst	300	11,420.58	303.8366	12,243.1	2417.003	15,018.3	3170.638	300.1178	5592.507	861.5824	13,222.67	300	22,134.76
	std	0	3413.27	2.249265	3607.385	823.5855	5030.939	1307.715	0.050225	2058.964	189.2264	3161.605	5.02 × 10^−14^	10,161.97
	median	300	9277.635	301.6691	9716.179	1106.994	11716.9	1422.259	300.0385	2317.485	745.6598	9788.562	300	14,883.97
	rank	1	9	4	10	6	12	7	3	8	5	11	2	13
C17-F4	mean	400	912.8675	404.5044	1301.509	406.3768	567.2482	423.8418	403.1612	411.1278	408.6941	404.3164	419.2569	413.954
	best	400	690.8376	401.1767	821.7783	402.3193	473.7955	406.1071	401.5111	405.7731	407.95	403.3764	400.1002	411.0716
	worst	400	1112.692	406.1874	1771.409	410.788	676.3611	469.7346	404.6409	426.8867	409.1638	405.7603	466.7173	417.4808
	std	0	209.9105	2.551559	438.1046	4.514927	107.3145	33.16825	1.758815	11.35517	0.562504	1.181624	34.54932	3.031633
	median	400	923.97	405.3267	1306.424	406.2	559.4181	409.7628	403.2464	405.9257	408.8312	404.0645	405.1051	413.6319
	rank	1	12	4	13	5	11	10	2	7	6	3	9	8
C17-F5	mean	501.2464	562.3634	542.2295	569.7676	512.4027	561.6767	539.2849	522.754	512.538	532.6659	551.6227	526.777	526.884
	best	500.9951	548.7241	525.743	555.7886	508.0904	541.4349	522.5012	509.838	508.2057	527.4	546.955	510.7172	522.3658
	worst	501.9917	571.2765	560.2126	584.1212	517.2861	592.3801	573.6615	536.4622	519.5033	536.0354	562.8635	549.6066	532.3901
	std	0.537048	11.20594	19.53923	17.01542	5.237015	24.42791	25.90124	12.01489	5.259868	4.100836	8.206794	19.38461	4.88069
	median	500.9993	564.7264	541.4811	569.5803	512.1171	556.4458	530.4886	522.3578	511.2215	533.6141	548.3362	523.3922	526.3901
	rank	1	12	9	13	2	11	8	4	3	7	10	5	6
C17-F6	mean	600	631.2585	616.6484	639.1286	601.1476	623.8678	622.2694	602.0665	601.0833	606.5967	616.5387	607.1419	609.8626
	best	600	627.7654	615.6829	636.0406	600.6833	614.4904	607.2346	600.4538	600.573	604.5743	602.8033	601.3022	606.6375
	worst	600	635.2624	619.101	643.2172	602.3052	638.8541	643.4482	604.1461	601.6524	609.7501	634.7421	618.513	613.9428
	std	0	3.529052	1.772351	3.485675	0.836288	11.35957	16.48107	1.793347	0.482948	2.550371	15.96967	8.437511	3.499868
	median	600	631.003	615.9048	638.6283	600.8009	621.0634	619.1973	601.8331	601.054	606.0311	614.3047	604.3763	609.435
	rank	1	12	9	13	3	11	10	4	2	5	8	6	7
C17-F7	mean	711.1267	802.7762	763.4548	800.7578	724.117	823.9431	760.1147	730.1162	725.4394	750.4728	716.8883	731.9086	735.8794
	best	710.6726	781.8022	742.6204	788.0238	720.0761	785.3852	749.5597	716.9869	717.224	746.1048	714.7148	725.0479	725.955
	worst	711.7995	821.2507	790.1525	812.9158	728.3524	863.7992	788.4426	748.6276	742.2706	758.2657	720.4627	743.0075	740.2679
	std	0.553542	18.15716	23.61747	12.62505	3.762887	36.81428	20.47025	14.39328	12.43131	5.870293	2.693729	8.863192	7.262378
	median	711.0174	804.0259	760.5232	801.0458	724.0197	823.294	751.2283	727.4252	721.1315	748.7603	716.1878	729.7895	738.6474
	rank	1	12	10	11	3	13	9	5	4	8	2	6	7
C17-F8	mean	801.4928	847.3854	829.9963	851.707	812.2457	846.5042	835.0363	811.4403	815.3054	836.332	819.1693	821.9628	816.2124
	best	800.995	838.3184	819.5556	840.9358	808.5515	830.9475	817.9146	807.1841	810.1642	829.6671	811.6011	815.1364	812.3596
	worst	801.9912	856.3685	845.1869	856.7313	814.3296	865.0493	846.7926	816.0273	820.0848	844.0017	826.6263	828.1893	823.6925
	std	0.621323	8.618492	11.69136	7.878719	2.87132	16.40915	13.40674	3.926693	4.487302	7.92135	6.910815	6.993148	5.494601
	median	801.4926	847.4274	827.6213	854.5804	813.0508	845.0101	837.7191	811.2749	815.4864	835.8295	819.2249	822.2628	814.3987
	rank	1	12	8	13	3	11	9	2	4	10	6	7	5
C17-F9	mean	900	1403.175	1177.093	1445.594	904.9995	1362.32	1357.11	900.7708	911.4786	911.3753	900	904.0802	904.9163
	best	900	1269.436	951.6727	1353.459	900.315	1157.773	1067.465	900.001	900.5514	906.9562	900	900.865	902.6913
	worst	900	1539.412	1632.062	1577.82	912.8331	1639.549	1627.449	902.9957	931.8665	919.2412	900	911.849	908.7317
	std	0	133.8949	340.7487	103.2504	6.089832	225.3145	254.7876	1.603621	15.87842	5.835298	0	5.668996	2.952723
	median	900	1401.927	1062.319	1425.548	903.4249	1325.979	1366.763	900.0433	906.7482	909.6519	900	901.8034	904.1211
	rank	1	11	8	12	5	10	9	2	7	6	1	3	4
C17-F10	mean	1006.179	2242.673	1740.928	2503.746	1492.149	1983.773	1976.532	1743.756	1690.998	2116.596	2217.678	1901.082	1681.718
	best	1000.284	2011.33	1460.938	2341.214	1373.017	1721.259	1428.434	1434.112	1513.627	1744.542	1951.564	1534.181	1395.362
	worst	1012.668	2443.533	2347.261	2846.267	1563.4	2224.291	2475.855	2221.473	1944.864	2390.909	2318.319	2287.687	2058.089
	std	7.194373	212.8061	450.1048	254.2606	96.90673	286.6101	547.4841	412.238	198.1502	296.9816	192.1263	334.4831	307.243
	median	1005.882	2257.915	1577.756	2413.751	1516.089	1994.772	2000.92	1659.719	1652.75	2165.467	2300.415	1891.231	1636.71
	rank	1	12	5	13	2	9	8	6	4	10	11	7	3
C17-F11	mean	1100	3931.298	1146.151	3844.336	1125.734	5248.161	1148.486	1126.173	1152.592	1148.443	1137.292	1141.417	2320.565
	best	1100	2748.781	1116.222	1441.219	1112.56	5107.121	1112.331	1105.277	1120.573	1135.997	1118.692	1130.687	1114.315
	worst	1100	5068.238	1196.85	6217.9	1155.93	5325.546	1169.558	1146.541	1222.149	1168.801	1165.288	1161.87	5742.625
	std	0	1127.521	38.35607	2320.262	22.13462	104.9245	28.56128	22.28304	51.17725	15.30439	21.49395	15.17738	2466.306
	median	1100	3954.085	1135.767	3859.113	1117.223	5279.989	1156.028	1126.437	1133.822	1144.486	1132.595	1136.556	1212.661
	rank	1	12	6	11	2	13	8	3	9	7	4	5	10
C17-F12	mean	1352.959	3.37 × 10^8^	1,050,072	6.73 × 10^8^	541,542	991,960.6	2,245,918	981,879.2	1,350,349	4,820,499	973,553.4	7780.181	577,271.4
	best	1318.646	75,442,697	339,707.7	1.49 × 10^8^	19,010.6	514,431.2	163,914.5	8485.281	43,408.79	1,290,069	452,782.8	2463.035	167,249.5
	worst	1438.176	5.89 × 10^8^	1,904,244	1.18 × 10^9^	847,465.2	1,217,819	3,725,865	3,084,075	2,113,661	8,533,694	1,646,391	13,344.2	1,018,988
	std	61.92816	2.81 × 10^8^	790,915.3	5.62 × 10^8^	394,461.6	358,486.6	1,789,591	1,535,516	986,202.2	4,146,912	54,6081.3	5357.753	377,994.9
	median	1327.506	3.42 × 10^8^	978,168.8	6.83 × 10^8^	649,846.1	1,117,796	2,546,947	417,478.1	1,622,163	4,729,116	897,520	7656.745	561,424
	rank	1	12	8	13	3	7	10	6	9	11	5	2	4
C17-F13	mean	1305.324	16,403,745	17,548.34	32,796,754	5244.269	12,211.23	7290.03	6478.772	9884.935	16,016.06	9667.689	6376.406	52,004.5
	best	1303.114	1,369,343	2657.747	2,722,927	3609.484	7298.455	3190.013	1382.32	6267.734	15,126.74	4875.201	2329.488	8210.392
	worst	1308.508	54,446,115	30,025.46	1.09 × 10^8^	6400.179	19,311.59	14,516.01	11,869.56	13,785.16	18,188.27	13,592.34	16,005.28	171,820.9
	std	2.456412	27,470,086	15,291.58	54,938,775	1438.543	5603.79	5580.27	5871.155	3329.694	1580.064	3982.217	7014.873	86382.3
	median	1304.837	4,899,761	18,755.07	9,792,476	5483.707	11,117.44	5727.048	6331.604	9743.423	15,374.63	10,101.61	3585.426	13,993.35
	rank	1	12	10	13	2	8	5	4	7	9	6	3	11
C17-F14	mean	1400.746	3965.806	1993.916	5169.404	1915.876	3297.27	1513.73	1564.223	2303.898	1582.307	5378.146	2923.968	12,441.63
	best	1400	3075.259	1666.562	4532.996	1433.46	1484.029	1478.206	1422.121	1459.571	1510.971	4457.979	1431.065	3622.123
	worst	1400.995	5446.187	2763.948	6650.15	2835.899	5395.317	1551.67	1966.952	4803.25	1611.243	7276.959	6599.163	24,730.34
	std	0.537676	1189.978	558.9709	1074.876	710.916	2248.705	40.57658	290.2479	1800.898	51.64711	1427.55	2669.493	9664.281
	median	1400.995	3670.889	1772.578	4747.234	1697.073	3154.867	1512.521	1433.91	1476.386	1603.507	4888.823	1832.823	10,707.02
	rank	1	10	6	11	5	9	2	3	7	4	12	8	13
C17-F15	mean	1500.331	9923.716	5127.088	13317.23	3864.416	6754.881	6005.951	1539.98	5620.1	1699.806	22873	8659.656	4412.735
	best	1500.001	2822.72	2046.732	2678.572	3145.722	2282.713	1991.354	1524.768	3476.988	1580.333	10,788.43	2809.879	1872.988
	worst	1500.5	17,572.85	12,126.03	29,059.94	4739.333	12,048.5	12,909.3	1551.486	6656.821	1785.449	34,294.88	14,195.79	7720.872
	std	0.254447	6588.979	5082.111	12,449.64	714.5172	4535.851	5143.584	12.61657	1579.156	108.7942	12137.14	5143.125	3142.31
	median	1500.413	9649.649	3167.794	10,765.2	3786.305	6344.154	4561.573	1541.833	6173.294	1716.721	23204.34	8816.476	4028.54
	rank	1	11	6	12	4	9	8	2	7	3	13	10	5
C17-F16	mean	1600.76	1998.51	1800.135	1997.635	1680.458	2027.117	1934.677	1806.505	1722.723	1673.458	2051.818	1909.049	1793.242
	best	1600.356	1927.649	1640.379	1809.456	1639.913	1850.597	1757.659	1720.858	1615.162	1648.669	1931.467	1812.419	1713.384
	worst	1601.12	2164.347	1911.539	2259.074	1709.675	2203.316	2057.119	1865.397	1815.303	1725.282	2237.85	2061.587	1822.905
	std	0.341437	120.0836	123.4574	205.2288	32.43533	172.9718	153.8331	66.0704	89.25039	38.59988	150.5739	124.7427	57.58986
	median	1600.781	1951.022	1824.31	1961.004	1686.121	2027.277	1961.965	1819.883	1730.215	1659.94	2018.978	1881.095	1818.339
	rank	1	11	6	10	3	12	9	7	4	2	13	8	5
C17-F17	mean	1700.099	1811.032	1748.702	1813.071	1734.143	1797.616	1835.539	1836.38	1765.464	1755.775	1840.193	1750.025	1753.483
	best	1700.02	1806.017	1732.879	1796.914	1720.932	1783.119	1770.312	1775.004	1723.38	1746.088	1745.794	1743.685	1750.49
	worst	1700.332	1816.495	1790.749	1821.878	1771.561	1808.017	1880.859	1939.243	1863.908	1765.275	1960.858	1756.411	1755.807
	std	0.1677	4.781214	30.37525	11.99733	26.97435	11.55907	51.90246	84.05218	71.29364	10.26317	118.527	5.882847	2.595165
	median	1700.022	1810.809	1735.591	1816.747	1722.039	1799.664	1845.492	1815.637	1737.285	1755.869	1827.061	1750.002	1753.817
	rank	1	9	3	10	2	8	11	12	7	6	13	4	5
C17-F18	mean	1805.36	2,720,945	11,379.46	5,427,103	10,610.98	11,572.34	22,285.37	20,036.95	19,045.42	28,187.83	9337.384	20,922.06	12,291.63
	best	1800.003	139,186.2	4700.101	268,745.2	4046.981	7197.034	6229.563	8374.975	6109.303	22,936.29	6176.433	2829.558	3358.621
	worst	1820.451	7,885,942	14,941.16	15,754,410	15,819.61	15,599.76	34,954.29	32,194.69	32,078.69	35,234.85	11,378.71	38,889.97	17,690.1
	std	10.87647	3,878,075	4962.863	7,754,807	5786.63	3777.008	14,959.81	12,120.25	14,233.07	6114.069	2399.699	20,119.65	6765.819
	median	1800.492	1,429,325	12,938.29	2,842,628	11,288.65	11,746.28	23,978.81	19,789.06	18,996.84	27290.1	9897.196	20,984.36	14,058.91
	rank	1	12	4	13	3	5	10	8	7	11	2	9	6
C17-F19	mean	1900.445	382,128.4	6480.047	670,534.1	5422.75	119643	33,241.73	1914.076	5218.417	4563.893	38,592.68	23850.92	5979.281
	best	1900.039	23,669.33	2163.674	43,727.29	2298.029	1946.892	7386.054	1908.976	1942.621	2036.535	10,666.06	2590.192	2198.397
	worst	1901.559	807,918.7	12,704.9	1,440,333	9059.158	238,893.5	60,779.89	1923.162	13,242.88	11,986.18	55,936.27	73,319.61	9503.385
	std	0.804778	363,558.4	5540.043	680,945.1	3724.541	146,862.2	23,690.54	7.245959	5842.633	5348.197	21,912.64	36,044.78	3257.444
	median	1900.09	348,462.7	5525.808	599,038.2	5166.907	118,865.8	32,400.49	1912.082	2844.083	2116.429	43,884.19	9746.935	6107.67
	rank	1	12	7	13	5	11	9	2	4	3	10	8	6
C17-F20	mean	2000.312	2204.87	2162.463	2212.433	2087.948	2197.531	2196.784	2132.936	2161.848	2068.592	2241.649	2160.956	2047.84
	best	2000.312	2147.953	2029.856	2156.715	2069.305	2101.643	2093.668	2044.722	2124.703	2058.09	2178.862	2137.978	2034.123
	worst	2000.312	2273.291	2280.509	2265.22	2117.006	2305.659	2274.207	2235.683	2234.319	2078.517	2330.323	2191.288	2055.236
	std	0	55.95046	121.8733	57.71282	22.09133	93.40801	93.27596	84.74551	53.40734	9.256346	79.64236	28.63668	10.52061
	median	2000.312	2199.118	2169.743	2213.898	2082.741	2191.412	2209.631	2125.669	2144.184	2068.881	2228.705	2157.279	2051
	rank	1	11	8	12	4	10	9	5	7	3	13	6	2
C17-F21	mean	2200	2289.451	2213.16	2263.977	2254.517	2319.303	2304.709	2250.653	2307.984	2295.016	2360.425	2313.231	2293.568
	best	2200	2243.728	2203.935	2222.833	2252.144	2220.235	2217.531	2200.007	2303.973	2203.545	2343.766	2305.548	2225.313
	worst	2200	2313.224	2237.184	2287.371	2256.934	2364.061	2346.83	2302.568	2312.721	2331.891	2376.94	2320.429	2326.589
	std	0	35.38769	17.36401	30.84939	2.191022	72.61342	63.60808	63.21616	3.888176	66.38128	14.98381	7.910979	49.80776
	median	2200	2300.426	2205.761	2272.853	2254.494	2346.458	2327.237	2250.019	2307.621	2322.315	2360.496	2313.473	2311.184
	rank	1	6	2	5	4	12	9	3	10	8	13	11	7
C17-F22	mean	2300.073	2735.26	2308.571	2887.391	2304.777	2694.741	2322.704	2286.437	2308.206	2318.672	2300.008	2312.66	2317.103
	best	2300	2612.518	2304.162	2688.154	2300.9	2442.249	2318.25	2232.7	2301.208	2312.71	2300	2300.609	2314.334
	worst	2300.29	2877.797	2310.632	3033.613	2308.929	2892.997	2329.991	2305.061	2321.374	2329.864	2300.032	2343.368	2321.375
	std	0.156805	135.1716	3.217222	157.2229	3.655692	217.4236	5.666251	38.73174	10.02473	8.486782	0.017512	22.17284	3.252344
	median	2300	2725.362	2309.746	2913.897	2304.639	2721.858	2321.286	2303.994	2305.122	2316.057	2300	2303.333	2316.352
	rank	3	12	6	13	4	11	10	1	5	9	2	7	8
C17-F23	mean	2600.919	2696.384	2640.282	2696.181	2613.729	2717.995	2646.632	2619.402	2613.171	2640.736	2783.332	2642.401	2653.732
	best	2600.003	2652.88	2629.345	2668.577	2611.49	2632.886	2629.516	2606.881	2607.585	2630.312	2721.168	2635.614	2634.636
	worst	2602.87	2721.482	2657.221	2734.934	2616.275	2760.405	2666.012	2630.427	2619.553	2649.718	2915.529	2653.755	2661.733
	std	1.427016	34.47961	14.2173	33.55836	2.476768	62.28981	21.24948	11.06696	6.702293	9.295428	98.69733	8.898797	13.97597
	median	2600.403	2705.587	2637.282	2690.607	2613.575	2739.345	2645.5	2620.151	2612.773	2641.457	2748.316	2640.118	2659.279
	rank	1	11	5	10	3	12	8	4	2	6	13	7	9
C17-F24	mean	2630.488	2766.593	2761.5	2840.118	2630.645	2666.605	2754.816	2680.964	2743.511	2750.251	2742.257	2759.547	2718.993
	best	2516.677	2707.392	2726.529	2815.839	2612.188	2534.895	2724.621	2502.024	2715.242	2733.589	2504.848	2748.125	2546.618
	worst	2732.32	2853.043	2783.053	2903.567	2641.946	2808.346	2788.924	2758.212	2758.78	2765.011	2890.232	2783.848	2807.47
	std	125.9143	75.9419	27.6873	45.77957	14.62564	158.6431	28.97391	129.9664	21.1953	16.62111	179.2091	17.7425	126.4467
	median	2636.477	2752.969	2768.209	2820.534	2634.224	2661.59	2752.86	2731.809	2750.01	2751.202	2786.974	2753.108	2760.941
	rank	1	12	11	13	2	3	9	4	7	8	6	10	5
C17-F25	mean	2932.639	3160.476	2914.357	3261.191	2918.565	3124.511	2908.677	2922.579	2938.422	2933.489	2922.742	2923.757	2951.355
	best	2898.047	3059.126	2899.047	3196.151	2915.169	2907.548	2772.937	2902.876	2922.403	2915.286	2904.47	2898.64	2936.383
	worst	2945.793	3377.692	2948.782	3332.197	2924.264	3623.252	2956.366	2943.701	2945.776	2951.903	2943.394	2946.519	2961.892
	std	24.95556	158.0947	24.97843	60.78711	4.621638	363.3763	97.86807	24.42714	11.68901	21.3807	22.74034	27.8157	11.82051
	median	2943.359	3102.544	2904.799	3258.209	2917.413	2983.623	2952.703	2921.87	2942.754	2933.383	2921.552	2924.935	2953.573
	rank	7	12	2	13	3	11	1	4	9	8	5	6	10
C17-F26	mean	2900	3590.504	2976.275	3717.955	3006.657	3588.502	3170.338	2900.141	3248.952	3192.893	3818.581	2903.878	2897.341
	best	2900	3253.229	2811.168	3408.598	2892.468	3133.274	2925.995	2900.108	2966.126	2911.514	2811.168	2811.168	2716.041
	worst	2900	3838.081	3145.308	4039.913	3275.942	4208.271	3563.03	2900.185	3862.063	3832.048	4284.157	3004.343	3100.218
	std	4.01 × 10^−13^	318.5314	206.0825	294.2025	194.9322	568.1313	301.0223	0.036937	445.8368	463.5612	737.6724	85.37276	210.3024
	median	2900	3635.352	2974.312	3711.654	2929.108	3506.23	3096.163	2900.136	3083.81	3014.005	4089.499	2900	2886.553
	rank	2	11	5	12	6	10	7	3	9	8	13	4	1
C17-F27	mean	3089.518	3202.346	3118.638	3224.783	3104.012	3175.498	3190.204	3091.534	3114.92	3113.947	3219.915	3133.99	3156.849
	best	3089.518	3155.296	3095.047	3125.526	3092.121	3101.851	3175.022	3089.702	3094.217	3095.122	3208.298	3096.756	3118.008
	worst	3089.518	3268.648	3176.831	3408.258	3131.827	3215.862	3201.44	3094.72	3172.873	3167.605	3240.504	3179.191	3213.141
	std	2.84 × 10^−13^	51.46529	42.05219	135.3805	20.18765	55.82681	11.91661	2.551364	41.79912	38.67197	15.48972	37.46758	43.47098
	median	3089.518	3192.721	3101.337	3182.674	3096.05	3192.139	3192.177	3090.856	3096.294	3096.53	3215.43	3130.007	3148.123
	rank	1	11	6	13	3	9	10	2	5	4	12	7	8
C17-F28	mean	3100	3613.12	3229.856	3748.016	3213.098	3563.939	3278.224	3232.355	3333.69	3314.747	3434.636	3296.217	3239.629
	best	3100	3564.242	3100	3669.487	3163.857	3398.145	3150.272	3100.118	3190.342	3208.737	3421.984	3173.571	3142.818
	worst	3100	3658.602	3376.998	3804.7	3236.846	3763.532	3377.485	3376.998	3397.699	3377.228	3452.218	3377.203	3494.4
	std	0	45.32491	132.4232	67.82996	36.51153	204.8201	126.2074	165.3185	104.0978	86.92067	15.1415	99.78275	184.2716
	median	3100	3614.818	3221.214	3758.937	3225.844	3547.04	3292.569	3226.152	3373.359	3336.512	3432.171	3317.047	3160.649
	rank	1	12	3	13	2	11	6	4	9	8	10	7	5
C17-F29	mean	3132.241	3314.139	3277.755	3364.373	3199.962	3231.62	3339.237	3199.563	3259.274	3209.074	3336.362	3260.107	3232.573
	best	3130.076	3287.353	3206.836	3296.097	3164.379	3164.684	3231.004	3141.96	3187.414	3164.081	3229.306	3166.223	3186.047
	worst	3134.841	3333.919	3355.015	3428.46	3239.695	3298.457	3479.7	3279.517	3368.515	3230.887	3612.426	3339.331	3279.39
	std	2.682921	24.34197	82.5024	73.75275	35.7886	59.16538	112.7108	62.92391	93.13042	33.84792	199.7285	84.89115	42.47972
	median	3132.023	3317.643	3274.584	3366.468	3197.887	3231.669	3323.123	3188.387	3240.583	3220.665	3251.858	3267.436	3232.427
	rank	1	10	9	13	3	5	12	2	7	4	11	8	6
C17-F30	mean	3418.734	2,094,369	280,491.5	3,496,344	394,645.1	584,643.1	943,812.6	288,243.6	890,238.3	57,836.15	744,606.5	368,495.7	1,452,803
	best	3394.682	1,139,771	99,730.8	787,314.7	15,318.09	106,985.2	4415.907	7241.996	32,113.8	28,026.15	572,501.3	6250.265	500,267.8
	worst	3442.907	3,222,881	730,437.6	5,522,322	582,379.3	1,236,001	3,562,526	1,098,507	1,288,325	96,953.6	950,837.1	730,472.4	3,309,498
	std	30.01454	927,639.1	325,084.1	2,142,747	278,341	518,513.2	1,889,224	584,012.6	637,919.8	36,384.93	169,923.6	451,146.3	1,431,174
	median	3418.673	2007,412	145,898.9	3,837,870	490,441.6	497,792.9	104,154.4	23613	1,120,257	53,182.43	727,543.8	368,630	1,000,724
	rank	1	12	3	13	6	7	10	4	9	2	8	5	11
Sum rank		38	325	177	347	106	282	239	116	188	191	238	183	197
Mean rank		1.31	11.2	6.10	12	3.66	9.72	8.24	4.00	6.48	6.59	8.21	6.31	6.79
Total rank		1	12	4	13	2	11	10	3	6	7	9	5	8

**Table 3:** Optimization results of CEC 2017 test suite (dimension = 30).

		KOA	WSO	AVOA	RSA	MPA	TSA	WOA	MVO	GWO	TLBO	GSA	PSO	GA
C17-F1	mean	100	2.49 × 10^10^	2952.398	3.89 × 10^10^	25,349.41	1.7 × 10^10^	1.61 × 10^9^	508,943.7	1.58 × 10^9^	5.84 × 10^9^	9,943,348	1.33 × 10^9^	1.69 × 10^8^
	best	100	2.14 × 10^10^	270.1836	3.47 × 10^10^	11,668.69	1.07 × 10^10^	1.27 × 10^9^	395,393.4	2.6 × 10^8^	3.69 × 10^9^	2400.729	3551.009	1.26 × 10^8^
	worst	100	3.11 × 10^10^	7250.693	4.79 × 10^10^	38,538.32	2.31 × 10^10^	2 × 10^9^	647,323.3	4.76 × 10^9^	8.71 × 10^9^	34,713,198	5.31 × 10^9^	2.33 × 10^8^
	std	8.87 × 10^−15^	4.89 × 10^9^	3536.471	6.56 × 10^9^	14,034.64	6.3 × 10^9^	4.03 × 10^8^	134,582.1	2.3 × 10^9^	2.26 × 10^9^	18,032,225	2.87 × 10^9^	49,948,590
	median	100	2.35 × 10^10^	2144.357	3.65 × 10^10^	25,595.31	1.7 × 10^10^	1.58 × 10^9^	496,529	6.51 × 10^8^	5.48 × 10^9^	2,528,896	3,024,996	1.58 × 10^8^
	rank	1	12	2	13	3	11	9	4	8	10	5	7	6
C17-F3	mean	300	92,153.76	42,326.24	69,683.06	1059.571	44,689.85	219,526.4	1696.502	39,453.42	32,842.34	90,744.25	30,201.43	158,298.6
	best	300	84,157.35	22,988.07	53,966.73	821.5916	42,342.1	181,622.8	1336.346	34,468.66	27,963.1	78,127.36	21,553	119,788.6
	worst	300	101,179	54,730.87	75,694.69	1300.824	47,088.12	252,193.8	2324.308	44,060.35	35,568.41	99,929.63	38,785.04	219,938.2
	std	0	9080.451	14,710.29	11,360.72	232.5882	2570.157	31,725.49	473.5833	4252.853	3701.235	10,628.4	8483.986	51,375.34
	median	300	91,639.33	45,793	74,535.41	1057.934	44,664.59	222,144.5	1562.677	39,642.33	33,918.93	92,460.01	30,233.85	146,733.9
	rank	1	11	7	9	2	8	13	3	6	5	10	4	12
C17-F4	mean	458.5616	6128.233	511.6871	9325.613	491.3039	4327.565	835.7871	494.7428	565.8432	883.973	587.5266	615.3381	793.266
	best	458.5616	3452.638	490.0475	5985.996	481.3822	1016.012	774.1829	487.3012	513.1528	687.9052	568.2553	512.664	743.8779
	worst	458.5616	8287.243	528.8878	13026.04	511.9059	7178.144	912.6984	507.5081	595.5328	1262.83	609.6755	793.8941	815.7684
	std	0	2165.688	17.41837	3158.382	15.15013	2812.58	68.3073	9.658623	39.12174	278.2517	19.52181	139.5195	36.4108
	median	458.5616	6386.526	513.9066	9145.206	485.9638	4558.052	828.1336	492.081	577.3437	792.5785	586.0879	577.3971	806.7089
	rank	1	12	4	13	2	11	9	3	5	10	6	7	8
C17-F5	mean	502.4874	827.2903	713.9748	864.4705	579.0909	779.0051	806.7678	613.4365	615.7996	756.6456	711.5144	625.9249	692.1274
	best	500.995	808.357	678.9904	839.7565	557.682	751.8996	779.2013	599.2833	577.3504	735.1627	693.0098	602.6682	645.5771
	worst	503.9798	847.5287	769.6813	896.8244	600.8691	810.9206	819.8405	646.7345	643.1417	781.6338	736.4281	672.8274	751.9071
	std	1.388273	17.6368	44.33189	29.45868	19.55685	30.05426	20.08862	24.1357	35.13228	24.27559	20.94377	34.45568	47.6562
	median	502.4874	826.6379	703.6137	860.6505	578.9063	776.6002	814.0148	603.8641	621.3532	754.893	708.3097	614.1021	685.5127
	rank	1	12	8	13	2	10	11	3	4	9	7	5	6
C17-F6	mean	600	675.4703	644.1356	678.461	603.0903	672.7694	672.0451	623.0537	611.238	640.938	653.3996	644.3518	628.5215
	best	600	674.2169	642.2589	673.4417	601.8882	658.2745	661.7335	611.8056	604.4229	634.2103	652.6949	632.8396	621.8723
	worst	600	676.7159	647.0704	684.7624	604.4175	681.2908	677.1697	635.0517	617.9433	651.9022	654.3359	654.5071	632.8719
	std	7.09 × 10^−14^	1.113644	2.247546	5.651016	1.187816	11.69702	7.612886	11.8271	6.014296	8.405992	0.781807	10.3672	5.178989
	median	600	675.4743	643.6065	677.8199	603.0277	675.7562	674.6386	622.6788	611.2929	638.8197	653.2839	645.0302	629.671
	rank	1	12	7	13	2	11	10	4	3	6	9	8	5
C17-F7	mean	733.478	1268.013	1124.657	1306.605	841.0554	1198.026	1276.514	848.0105	877.9558	1057.553	958.8085	871.0261	955.2704
	best	732.8186	1222.778	1014.897	1293.645	815.4113	1060.213	1235.449	797.4056	811.3341	974.0064	914.078	850.9112	917.6521
	worst	734.5199	1302.862	1278.106	1328.756	892.1383	1340.273	1353.189	918.1625	916.1328	1130.318	1025.628	896.943	1007.187
	std	0.814948	37.47192	125.7292	16.8812	37.51134	131.312	59.11282	55.88792	49.56736	88.24602	52.88124	21.53589	40.50579
	median	733.2867	1273.207	1102.813	1302.01	828.3361	1195.808	1258.709	838.2369	892.1782	1062.945	947.7642	868.125	948.1212
	rank	1	11	9	13	2	10	12	3	5	8	7	4	6
C17-F8	mean	803.3298	1070.174	942.2719	1105.554	886.447	1045.612	1018.567	889.0451	887.7806	1011.206	953.4965	917.6519	976.862
	best	801.2023	1055.725	913.5956	1086.05	880.0891	1003.491	964.9791	859.9631	881.1796	993.2392	930.3884	906.5052	961.5987
	worst	804.1574	1089.433	962.762	1131.271	894.2287	1144.329	1058.264	917.3973	895.4335	1042.625	978.9794	932.744	996.3589
	std	1.535629	16.69147	24.14288	24.80971	6.318887	71.82849	43.10224	27.18019	6.702496	23.36502	23.19143	12.62398	18.97697
	median	803.9798	1067.769	946.365	1102.447	885.7352	1017.314	1025.513	889.41	887.2546	1004.481	952.3092	915.6791	974.7452
	rank	1	12	6	13	2	11	10	4	3	9	7	5	8
C17-F9	mean	900	10,428.87	4615.015	10,107.64	1075.025	10,925.08	10487.61	5212.126	2015.128	5513.265	3910.222	3406.802	1272.37
	best	900	8917.718	3422.76	9859.698	928.2135	6674.676	8027.565	4160.937	1504.993	3995.937	3401.26	2052.368	1071.172
	worst	900	11,851.73	5252.057	10,233.16	1219.778	14,741.2	12,497.63	7944.628	2760.734	8299.525	4694.308	5168.799	1472.054
	std	7.09 × 10^−14^	1319.296	884.851	181.7597	145.6568	3601.656	2430.474	1973.745	658.5688	2105.111	615.622	1428.948	203.3894
	median	900	10,473	4892.621	10168.85	1076.054	111,42.23	10,712.61	4371.469	1897.392	4878.799	3772.66	3203.022	1273.127
	rank	1	11	7	10	2	13	12	8	4	9	6	5	3
C17-F10	mean	2293.267	6968.874	5292.417	7618.404	3904.89	6343.463	6283.162	4530.69	4662.67	7637.039	4718.957	4901.116	5947.831
	best	1851.756	6395.829	4601.986	6781.76	3569.884	4998.608	5444.387	4262.331	4179.57	7294.433	4471.779	4672.72	5493.727
	worst	2525.027	7274.901	5750.32	8221.579	4309.538	6917.445	7526.832	4906.336	4954.674	7810.147	5116.464	5348.142	6464.514
	std	324.6445	424.0634	597.1511	655.0785	369.1495	974.92	997.2449	345.3045	366.0832	251.5575	328.7644	330.2447	496.3163
	median	2398.142	7102.382	5408.681	7735.139	3870.069	6728.9	6080.714	4477.045	4758.219	7721.789	4643.792	4791.8	5916.541
	rank	1	11	7	12	2	10	9	3	4	13	5	6	8
C17-F11	mean	1102.987	7176.983	1250.189	8409.622	1166.464	4925.027	7473.318	1303.696	2139.493	1942.773	2806.902	1242.149	8757.885
	best	1100.995	5915.713	1186.572	6856.566	1121.261	3511.464	5386.337	1262.477	1375.234	1564.539	2184.741	1214.111	3247.773
	worst	1105.977	8212.328	1311.123	9458.04	1198.506	7406.649	11,036.34	1343.487	4172.022	2640.713	3444.25	1268.793	16,401.46
	std	2.32642	1091.276	56.12963	1288.136	36.05235	1891.211	2661.731	49.30714	1466.531	515.0742	641.6108	28.63459	6093.036
	median	1102.487	7289.945	1251.531	8661.941	1173.044	4390.997	6735.297	1304.409	1505.358	1782.92	2799.309	1242.846	7691.156
	rank	1	10	4	12	2	9	11	5	7	6	8	3	13
C17-F12	mean	1744.553	6.67 × 10^9^	19,805,086	1.04 × 10^10^	20,633.44	4.81 × 10^9^	2.35 × 10^8^	10,662,962	49,904,305	2.87 × 10^8^	1.89 × 10^8^	2,434,411	7,299,327
	best	1721.81	5.51 × 10^9^	2,786,976	9.23 × 10^9^	14,762.4	2.48 × 10^9^	60150408	4,951,435	4,843,965	1.83 × 10^8^	36548589	263,184.6	5,054,160
	worst	1764.937	8.47 × 10^9^	48,369,635	1.3 × 10^10^	26,305.47	6.3 × 10^9^	4.7 × 10^8^	25,798,820	1.05 × 10^8^	4.98 × 10^8^	6.04 × 10^8^	4,840,042	9,554,266
	std	21.78111	1.37 × 10^9^	21,685,993	1.95 × 10^9^	5316.077	1.78 × 10^9^	2.04 × 10^8^	10,922,498	47,029,306	1.54 × 10^8^	2.99 × 10^8^	2,133,734	2,205,980
	median	1745.733	6.35 × 10^9^	14,031,866	9.58 × 10^9^	20,732.95	5.24 × 10^9^	2.06 × 10^8^	5,950,797	45,060,704	2.33 × 10^8^	57944215	2,317,209	7,294,441
	rank	1	12	6	13	2	11	9	5	7	10	8	3	4
C17-F13	mean	1315.791	5.42 × 10^9^	142,111.3	1 × 10^10^	1860.563	1.39 × 10^9^	858,772.9	86,428.26	716,806.2	83,718,700	34,704.4	30,802.39	11,311,917
	best	1314.587	2.64 × 10^9^	78,705.99	5.26 × 10^9^	1599.709	18,730,491	405,245.9	34,645.94	86,601.55	58,138,598	28,163.04	12,779.66	3,069,111
	worst	1318.646	7.6 × 10^9^	224,731.1	1.23 × 10^10^	2371.471	4.82 × 10^9^	1,269,702	173,553.6	2,224,190	1.23 × 10^8^	50,752.28	69,517.33	24,331,862
	std	2.092732	2.22 × 10^9^	65,540.46	3.48 × 10^9^	376.9899	2.49 × 10^9^	487,133.8	70,503.7	1,100,210	30,542,225	11,691.81	28,221.27	9,848,338
	median	1314.967	5.73 × 10^9^	132,504	1.13 × 10^10^	1735.535	3.58 × 10^8^	880,071.8	68,756.73	278,216.3	76,643,172	29,951.14	20,456.29	8,923,348
	rank	1	12	6	13	2	11	8	5	7	10	4	3	9
C17-F14	mean	1423.017	1,797,166	257,250.7	2,082,651	1439.516	1,113,810	2,108,486	19,356.09	505,563.4	132,706.9	1,084,630	17,864.1	1,903,549
	best	1422.014	1,108,273	36,037.67	1,046,798	1436.282	797,013.4	34,119.66	4805.772	32,658.35	77,153.62	703,827	3083.949	315,132.9
	worst	1423.993	2,274,971	595,484.3	3,101,244	1444.053	1,573,489	6,441,173	32,904.74	1,083,474	152,680.3	1,637,610	32,561.19	3,209,189
	std	0.873477	590,204.9	266,785.8	1,068,141	3.836186	385,143.4	3,179,950	13,081.47	576,571.5	40,043.56	474,950	13,917.18	1,442,616
	median	1423.03	1,902,710	198,740.4	2,091,282	1438.864	1,042,369	979,324.6	19,856.93	453,060.4	150,496.8	998,541.5	17,905.64	2,044,936
	rank	1	10	6	12	2	9	13	4	7	5	8	3	11
C17-F15	mean	1503.129	2.88 × 10^8^	35,569.23	5.66 × 10^8^	1612.888	13,622,278	4,780,527	40,622.22	14,998,288	4,865,200	15,307.25	4607.767	905,696.1
	best	1502.462	2.49 × 10^8^	10,436.55	4.89 × 10^8^	1577.289	5,366,361	220,281.9	23,546.64	93,188.24	1,104,763	10,895.36	1892.48	166,303.4
	worst	1504.265	3.19 × 10^8^	57,716.98	6.25 × 10^8^	1628.803	31,688,091	15,521,689	67,155.27	56,155,773	9,158,236	20,732.11	8499.218	2,029,134
	std	0.924686	37401288	21,580.75	72366016	25.84869	13,131,914	7,844,999	20,427.96	29,669,958	3,569,005	4444.669	3162.61	921,310.6
	median	1502.893	2.92 × 10^8^	37061.7	5.76 × 10^8^	1622.73	8,717,330	1,690,069	35,893.49	1,872,096	4,598,900	14,800.76	4019.686	713,673.5
	rank	1	12	5	13	2	10	8	6	11	9	4	3	7
C17-F16	mean	1663.469	4179.978	2931.478	4803.049	2008.781	3188.323	4109.782	2540.403	2498.513	3370.786	3562.069	2865.142	2883.088
	best	1614.72	3864.085	2506.541	4063.573	1726.769	2785.57	3390.133	2316.58	2354.879	3186.311	3383.684	2632.807	2554.441
	worst	1744.118	4441.066	3426.897	5467.226	2248.557	3431.42	4915.665	2791.01	2613.187	3592.217	3727.743	3130.503	3214.078
	std	66.97934	285.5894	409.083	811.8548	253.5446	308.6033	679.296	221.1256	142.7455	193.5071	165.786	271.8786	346.6355
	median	1647.519	4207.381	2896.238	4840.699	2029.899	3268.152	4066.665	2527.01	2512.993	3352.309	3568.425	2848.63	2881.917
	rank	1	12	7	13	2	8	11	4	3	9	10	5	6
C17-F17	mean	1728.099	3324.479	2438.547	3613.262	1858.056	3196.77	2794.085	2065.676	1925.308	2173.563	2486.032	2307.397	2137.267
	best	1718.761	2752.707	2299.939	3251.306	1752.386	2197.969	2338.319	2016.464	1801.98	1956.937	2390.162	2082.368	2092.547
	worst	1733.659	4032.229	2548.404	4253.792	1916.907	5812.541	3103.359	2208.975	2067.097	2455.83	2629.866	2682.335	2204.127
	std	7.250066	588.8234	117.6491	490.701	78.4514	1887.423	353.6048	103.2636	136.4408	228.6407	126.3251	291.0375	55.36113
	median	1729.987	3256.489	2452.922	3473.974	1881.465	2388.285	2867.33	2018.633	1916.078	2140.743	2462.051	2232.443	2126.196
	rank	1	12	8	13	2	11	10	4	3	6	9	7	5
C17-F18	mean	1825.696	26,931,134	2,510,229	30,965,156	1893.241	34,433,844	5,592,013	606,481.9	397,606.5	1,578,660	488,013.6	130,103.3	3,454,546
	best	1822.524	7,758,022	267,396.5	10,011,130	1871.842	1,262,746	1,884,521	152,677.2	74,409.88	732,924.3	273,634.5	92,598.72	2,696,975
	worst	1828.42	52,301,633	5,008,394	60,834,279	1905.758	65,253,789	11,541,782	1,641,661	1,021,471	1,984,645	950,086.7	154,354	5,063,684
	std	2.920243	21,282,724	2,401,473	23,292,806	16.42987	38,406,296	4,485,278	750,625.6	481,717.9	622,015.2	337,280	29,181.38	1,172,748
	median	1825.92	23,832,440	2,382,564	26,507,607	1897.682	35,609,421	4,470,874	315,794.9	247,272.6	1,798,535	364,166.6	136,730.3	3,028,763
	rank	1	11	8	12	2	13	10	6	4	7	5	3	9
C17-F19	mean	1910.989	5.5 × 10^8^	64,244.08	9.28 × 10^8^	1923.18	2.79 × 10^8^	13,576,235	890,246	3,821,835	5,449,857	77,576.92	42,261.41	1,536,207
	best	1908.84	4.12 × 10^8^	13,773.69	6.7 × 10^8^	1920.673	3464988	1,766,689	22,555.99	67,193.05	2,828,858	42,121.24	8400.832	607,052
	worst	1913.095	7.16 × 10^8^	142,987	1.41 × 10^9^	1927.772	7.73 × 10^8^	23,442,333	2,001,464	12,323,882	7,746,868	104,364.3	126,378.5	2,729,001
	std	2.088116	1.65 × 10^8^	60,833.73	3.53 × 10^8^	3.409684	3.84 × 10^8^	10,683,239	1,040,847	6,167,780	2,614,547	28,005.75	60,818.97	967,255
	median	1911.01	5.37 × 10^8^	50,107.82	8.18 × 10^8^	1922.138	1.7 × 10^8^	14,547,958	768,481.8	1,448,133	5,611,851	81,911.05	17,133.15	1,404,386
	rank	1	12	4	13	2	11	10	6	8	9	5	3	7
C17-F20	mean	2065.787	2861.078	2609.892	2912.496	2171.656	2814.376	2802.585	2580.819	2361.243	2764.973	2966.569	2525.574	2456.993
	best	2029.521	2772.475	2456.75	2741.1	2059.851	2675.689	2611.744	2358.871	2193.895	2683.202	2608.142	2475.735	2409.455
	worst	2161.126	2969.379	2833.462	3016.856	2260.42	2958.251	2974.494	2972.518	2523.041	2881.522	3428.076	2650.355	2491.316
	std	68.78908	87.95898	176.5827	130.5254	90.25521	126.4284	167.7548	291.3799	145.3611	100.6955	371.7545	90.13694	37.95021
	median	2036.25	2851.229	2574.679	2946.014	2183.176	2811.782	2812.052	2495.944	2364.018	2747.584	2915.028	2488.104	2463.601
	rank	1	11	7	12	2	10	9	6	3	8	13	5	4
C17-F21	mean	2308.456	2608.644	2435.242	2663.434	2363.914	2525.058	2597.048	2401.026	2386.5	2487.264	2558.71	2429.096	2484.514
	best	2304.034	2518.58	2221.839	2588.295	2354.475	2308.061	2523.336	2366.7	2352.907	2475.477	2541.004	2410.516	2452.745
	worst	2312.987	2668.213	2585.45	2752.462	2379.402	2653.222	2660.071	2429.705	2401.211	2497.343	2593.162	2442.179	2533.053
	std	4.819332	76.35051	165.502	77.25403	11.8397	164.6534	72.8331	28.30641	24.66245	11.63614	25.27778	16.83741	36.99578
	median	2308.402	2623.892	2466.84	2656.49	2360.89	2569.475	2602.391	2403.85	2395.942	2488.118	2550.336	2431.845	2476.128
	rank	1	12	6	13	2	9	11	4	3	8	10	5	7
C17-F22	mean	2300	7730.487	5617.266	7498.517	2302.796	8485.27	7178.241	3880.696	2685.993	5534.065	6147.871	4769.329	2684.245
	best	2300	7406.201	2302.919	6514.137	2301.824	8264.458	6264.079	2306.212	2562.444	2704.811	3925.426	2450.695	2612.661
	worst	2300	8236.5	6897.797	8487.871	2304.438	8589.519	7993.792	5835.971	2939.979	8670.963	7124.591	7013.27	2739.565
	std	0	383.5138	2391.94	916.8349	1.267341	165.0934	776.8943	1992.539	186.6203	3511.039	1611.848	2267.796	67.97661
	median	2300	7639.625	6634.174	7496.029	2302.461	8543.551	7227.546	3690.3	2620.775	5380.243	6770.735	4806.675	2692.377
	rank	1	12	8	11	2	13	10	5	4	7	9	6	3
C17-F23	mean	2655.081	3170.804	2916.435	3223.533	2646.423	3175.484	3031.962	2734.376	2747.574	2894.308	3724.216	2891.062	2962.989
	best	2653.745	3088.315	2811.411	3171.39	2478.867	3061.316	2861.72	2691.678	2728.555	2873.272	3620.24	2859.001	2934.863
	worst	2657.377	3249.328	3082.548	3299.152	2710.309	3365.1	3127.898	2762.31	2767.576	2942.516	3826.997	2940.298	3024.002
	std	1.786778	81.79862	128.3865	60.06802	121.0374	144.8298	127.7156	32.64114	18.18122	35.37896	118.3582	40.56704	44.366
	median	2654.6	3172.787	2885.891	3211.795	2698.258	3137.761	3069.114	2741.758	2747.083	2880.722	3724.813	2882.475	2946.546
	rank	2	10	7	12	1	11	9	3	4	6	13	5	8
C17-F24	mean	2831.409	3296.848	3158.037	3393.084	2881.606	3263.916	3105.656	2902.994	2917.582	3034.353	3343.978	3119.77	3211.401
	best	2829.992	3260.373	3024.392	3307.127	2866.584	3158.668	3043.783	2856.611	2905.496	3011.572	3308.343	3046.659	3120.381
	worst	2832.366	3372.507	3307.527	3542.546	2888.197	3313.313	3130.843	2924.994	2924.388	3069.382	3380.441	3229.636	3287.664
	std	1.238124	55.19726	134.4146	117.6224	10.96633	77.97751	44.80036	33.87131	9.179607	26.62799	34.43815	84.7416	83.90434
	median	2831.64	3277.257	3150.115	3361.332	2885.822	3291.842	3123.998	2915.185	2920.222	3028.23	3343.564	3101.392	3218.78
	rank	1	11	8	13	2	10	6	3	4	5	12	7	9
C17-F25	mean	2886.698	3898.903	2907.836	4500.07	2891.104	3446.751	3074.216	2908.624	2989.141	3067.347	2991.194	2894.634	3099.011
	best	2886.691	3536.836	2894.158	3919.097	2884.617	3083.235	3039.124	2884.613	2952.551	2951.169	2980.071	2887.569	3082.866
	worst	2886.707	4170.01	2945.463	5274.43	2897.059	3824.188	3092.665	2970.316	3057.272	3199.466	3003.259	2911.21	3110.566
	std	0.00822	285.7001	27.13135	610.0829	6.077511	391.4253	27.21077	44.57098	52.48505	128.3309	10.34894	12.00107	13.22934
	median	2886.698	3944.383	2895.861	4403.376	2891.37	3439.79	3082.537	2889.784	2973.372	3059.377	2990.723	2889.879	3101.307
	rank	1	12	4	13	2	11	9	5	6	8	7	3	10
C17-F26	mean	3578.65	8951.361	7176.459	9515.634	2976.112	8524.239	8181.363	4738	4524.954	5828.595	7315.992	4796.903	4360.868
	best	3559.841	8541.253	5941.453	8710.682	2973.861	7888.519	7475.009	4405.337	4144.872	4500.499	6305.049	3546.88	3990.712
	worst	3607.686	9687.167	7903.144	10,940.76	2979.46	8925.221	9002.183	5345.139	5113.309	7089.473	7842.161	6284.299	4813.412
	std	24.61688	577.1178	932.4917	1131.659	2.89745	480.5102	677.8715	471.9772	446.6385	1282.693	774.509	1381.508	372.9654
	median	3573.536	8788.512	7430.62	9205.547	2975.565	8641.608	8124.131	4600.762	4420.818	5862.204	7558.38	4678.216	4319.674
	rank	2	12	8	13	1	11	10	5	4	7	9	6	3
C17-F27	mean	3207.018	3595.037	3349.708	3744.391	3214.319	3463.398	3419.111	3230.47	3248.194	3313.698	4903.936	3275.914	3450.04
	best	3200.749	3538.093	3266.538	3474.172	3200.956	3334.416	3255.937	3212.443	3239.412	3239.11	4470.334	3238.363	3375.739
	worst	3210.656	3691.737	3422.578	4020.914	3233.651	3703.463	3540.445	3255.803	3262.719	3383.508	5219.687	3316.924	3493.865
	std	5.023361	74.0999	88.62389	253.3399	16.22388	177.9035	131.9415	19.66992	10.89568	64.60731	396.689	36.71613	55.61351
	median	3208.335	3575.159	3354.858	3741.239	3211.335	3407.856	3440.031	3226.816	3245.322	3316.086	4962.861	3274.185	3465.278
	rank	1	11	7	12	2	10	8	3	4	6	13	5	9
C17-F28	mean	3100	4715.982	3259.465	5591.034	3209.553	4117.781	3425.654	3250.611	3578.289	3647.974	3505.382	3320.401	3564.58
	best	3100	4488.68	3229.694	5292.726	3193.586	3580.19	3366.85	3215.783	3386.277	3503.148	3436.694	3190.728	3514.318
	worst	3100	4962.36	3290.424	5903.482	3238.681	4666.198	3478.795	3282.413	4047.397	3979.443	3649.532	3519.049	3619.148
	std	2.84 × 10^−13^	219.3937	26.8343	315.3407	21.78097	543.4944	52.65941	29.60263	340.0468	241.5308	105.1355	164.2269	53.83889
	median	3100	4706.444	3258.871	5583.963	3202.972	4112.368	3428.485	3252.125	3439.74	3554.653	3467.65	3285.913	3562.427
	rank	1	12	4	13	2	11	6	3	9	10	7	5	8
C17-F29	mean	3353.75	5321.805	4294.249	5533.458	3646.04	5169.464	5021.186	3824.482	3774.045	4466.786	4998.063	4137.498	4251.863
	best	3325.385	4887.175	3953.036	4925.474	3498.469	4645.242	4760.858	3700.725	3694.296	4148.96	4734.963	3945.945	3881.835
	worst	3370.797	5782.959	4501.103	6368.181	3780.651	6023.404	5187.571	3942.033	3889.131	4932.794	5246.778	4373.878	4596.038
	std	21.27976	466.7615	262.5544	766.6237	134.739	697.3597	197.3984	110.3398	94.00128	361.6177	296.0413	190.8879	345.3411
	median	3359.41	5308.544	4361.429	5420.09	3652.519	5004.604	5068.157	3827.585	3756.376	4392.696	5005.255	4115.085	4264.79
	rank	1	12	7	13	2	11	10	4	3	8	9	5	6
C17-F30	mean	5007.854	1.36 × 10^9^	1,359,178	2.69 × 10^9^	7559.769	36,618,487	37,367,034	2,947,714	6,078,409	36,074,281	2,156,344	259,931.3	669,154.3
	best	4955.449	1 × 10^9^	479,425.3	1.93 × 10^9^	6312.163	12,519,461	7,452,113	529,406.7	1,356,066	19,310,289	1,882,361	7470.567	185,303.8
	worst	5086.396	1.5 × 10^9^	2,406,694	2.97 × 10^9^	10,000.4	85,561,300	59,876,989	4,220,278	16,413,710	75,668,870	2,594,450	983,374.7	1,279,873
	std	63.73953	2.6 × 10^8^	870,882.7	5.47 × 10^8^	1868.254	35,834,239	23,618,797	1,779,169	7,516,367	28,689,990	331,326.2	521,521	575,976.5
	median	4994.785	1.48 × 10^9^	1,275,296	2.93 × 10^9^	6963.255	24,196,594	41,069,518	3,520,585	3,271,931	24,658,982	2,074,283	24,440	605,720.2
	rank	1	12	5	13	2	10	11	7	8	9	6	3	4
Sum rank		31	334	182	361	57	305	284	128	151	232	231	139	204
Mean rank		1.07	11.5	6.28	12.4	1.97	10.5	9.79	4.41	5.21	8.00	7.97	4.79	7.03
Total rank		1	12	6	13	2	11	10	3	5	9	8	4	7

**Table 4:** Optimization results of CEC 2017 test suite (dimension = 50).

		KOA	WSO	AVOA	RSA	MPA	TSA	WOA	MVO	GWO	TLBO	GSA	PSO	GA
C17-F1	mean	100	5.65 × 10^10^	8,732,323	8.85 × 10^10^	5,320,127	3.6 × 10^10^	7.27 × 10^9^	3,840,463	8.84 × 10^9^	1.96 × 10^10^	1.62 × 10^10^	2.39 × 10^9^	9.82 × 10^9^
	best	100	5.04 × 10^10^	1,039,624	7.74 × 10^10^	2,053,288	3.31 × 10^10^	4.29 × 10^9^	2,748,680	6.37 × 10^9^	1.33 × 10^10^	1.29 × 10^10^	9.81 × 10^08^	9.35 × 10^9^
	worst	100	6.05 × 10^10^	23,107,831	9.67 × 10^10^	13,489,625	3.87 × 10^10^	1.09 × 10^10^	4,780,327	1.21 × 10^10^	2.64 × 10^10^	1.94 × 10^10^	3.19 × 10^9^	1.06 × 10^10^
	std	0	4.79 × 10^9^	10,597,589	9.11 × 10^9^	5,928,769	2.5 × 10^9^	3.37 × 10^9^	903,719.7	2.58 × 10^9^	6.88 × 10^9^	2.85 × 10^9^	1.05 × 10^9^	6.22 × 10^8^
	median	100	5.75 × 10^10^	5,390,919	8.99 × 10^10^	2,868,796	3.6 × 10^10^	6.96 × 10^9^	3,916,422	8.44 × 10^9^	1.93 × 10^10^	1.62 × 10^10^	2.7 × 10^9^	9.68 × 10^9^
	rank	1	12	4	13	3	11	6	2	7	10	9	5	8
C17-F3	mean	300	149,583.9	138,352.8	149,030.3	16,943.78	103,002.4	220,840.5	43,670.63	122,679.4	92,792.69	167,990.6	136,635.8	248,646.8
	best	300	128,298.8	106,309.5	135,194	14,639.09	90,497.96	166,559	34,628.8	107,779	70,179.53	151,707.6	102,695.6	207,248.4
	worst	300	172,029	168,328.7	162,452.7	19,994.51	109,817.3	336,875	54,310.2	137,706	105,874.8	189,799.9	178,041.7	285,707.9
	std	0	19,900.14	30,273.96	13,080.54	2598.191	9650.742	86,695.78	8871.025	13,217.26	17,616.45	19,901.6	35,320.14	34,727.35
	median	300	149,004	139,386.6	149,237.2	16,570.75	105,847.1	189,964	42,871.76	122,616.4	97,558.22	165,227.4	132,903	250,815.5
	rank	1	10	8	9	2	5	12	3	6	4	11	7	13
C17-F4	mean	470.3679	13,956.83	684.8009	22,448.11	527.6775	7871.064	1857.265	557.9105	1381.66	2664.031	2918.089	985.3263	1465.974
	best	428.5127	10,846.92	669.6698	14,824.78	492.1734	6310.075	1187.093	521.0501	1032.473	1515.614	2439.486	669.7292	1268.232
	worst	525.7252	15,889.86	708.8812	26,809.34	579.9513	10,160.22	2218.682	629.8218	1684.796	4543.687	3103.746	1738.409	1585.165
	std	53.57701	2437.601	19.91459	5912.057	44.54252	1758.579	499.2023	53.51063	317.6341	1438.256	346.4791	545.1676	150.7205
	median	463.6168	14,545.27	680.3262	24,079.17	519.2927	7506.981	2011.642	540.3851	1404.685	2298.413	3064.563	766.5837	1505.249
	rank	1	12	4	13	2	11	8	3	6	9	10	5	7
C17-F5	mean	504.7261	1065.433	837.4285	1092.993	722.7024	1109.837	929.9259	725.1277	712.5915	970.3472	788.6582	772.5602	869.4968
	best	503.9798	1034.652	808.7226	1075.198	645.7768	975.5303	891.6619	655.8271	686.7938	930.8254	739.0804	721.1544	841.164
	worst	505.9698	1103.502	876.4592	1105.322	783.0094	1217.212	953.6909	831.6686	739.3389	996.3264	823.1554	833.2044	889.5473
	std	1.029571	35.75922	31.60248	14.9716	62.21624	126.9977	29.8511	85.07309	30.38572	31.62067	42.82098	49.85016	24.82183
	median	504.4773	1061.789	832.2661	1095.727	731.0116	1123.303	937.1754	706.5076	712.1166	977.1185	796.1986	767.941	873.638
	rank	1	11	7	12	3	13	9	4	2	10	6	5	8
C17-F6	mean	600	689.4033	656.5336	691.3502	610.6613	684.4566	691.9562	635.373	621.4235	660.3259	654.4208	650.3345	645.6004
	best	600	686.586	652.0101	689.2108	608.0459	665.3258	686.9105	625.7742	616.0881	648.3769	649.8097	648.1898	633.4675
	worst	600	694.0688	661.6627	694.1036	614.1181	700.135	699.6824	657.9483	630.6965	668.465	657.1603	653.6786	657.4692
	std	0	3.733946	4.819692	2.475418	2.810919	16.71551	5.995706	16.56411	7.084689	9.290449	3.495826	2.681362	10.85583
	median	600	688.4793	656.2309	691.0432	610.2406	686.1829	690.6159	628.8848	619.4548	662.2308	655.3566	649.7347	645.7324
	rank	1	11	8	12	2	10	13	4	3	9	7	6	5
C17-F7	mean	756.7298	1731.165	1612.083	1825.017	1012.626	1627.768	1651.629	1036.242	1047.359	1435.796	1372.132	1173.181	1274.479
	best	754.7543	1707.594	1545.095	1748.785	959.0639	1484.474	1592.429	1000.577	1025.438	1316.109	1213.45	1022.934	1200.637
	worst	758.3522	1761.078	1674.633	1922.444	1057.935	1768.548	1733.263	1064.965	1065.255	1493.97	1493.801	1392.142	1322.701
	std	1.678837	24.06938	59.46511	80.53832	51.74499	143.179	71.00264	29.43994	20.11823	87.36261	136.5406	172.2738	58.16152
	median	756.9065	1727.993	1614.302	1814.419	1016.753	1629.026	1640.413	1039.713	1049.372	1466.552	1390.637	1138.824	1287.289
	rank	1	12	9	13	2	10	11	3	4	8	7	5	6
C17-F8	mean	805.721	1383.747	1105.554	1409.746	998.4823	1400.121	1294.188	1009.222	1020.516	1291.782	1120.587	1041.767	1231.146
	best	802.9849	1329.89	1061.81	1379.857	969.3698	1306.445	1168.316	971.7921	987.8738	1238.631	1112.55	1001.552	1191.784
	worst	810.9445	1425.038	1150.513	1430.397	1028.234	1526.639	1397.851	1076.128	1056.699	1344.626	1134.428	1103.928	1253.498
	std	3.864789	46.39957	54.21158	23.03374	33.11967	102.6223	102.3809	49.57683	33.2358	47.41642	10.58349	52.43473	29.22653
	median	804.4773	1390.03	1104.946	1414.365	998.1629	1383.7	1305.294	994.4836	1018.745	1291.936	1117.686	1030.794	1239.651
	rank	1	11	6	13	2	12	10	3	4	9	7	5	8
C17-F9	mean	900	34,017.31	12,532.18	34,198.38	3177.381	35,681.16	31,068.86	18,528.59	6510.378	22,640.43	10,076.76	9719.426	12,073.9
	best	900	32,673.96	11,946.23	32,133.22	2002.422	32,892.84	28,919.61	9937.486	5666.427	17447.6	9185.167	9007.899	9942.128
	worst	900	37,146.23	13,346.71	35,882.85	4583.441	39,793.84	36,333.24	24,489.87	7408.203	26,631.57	10,878.18	11,039.89	13,899.21
	std	1 × 10^−13^	2290.983	654.6614	1919.083	1152.366	3213.01	3805.245	7397.037	977.6157	4122.296	760.6097	987.5776	2266.459
	median	900	33,124.53	12,417.9	34,388.73	3061.83	35,018.98	29511.3	19,843.5	6483.442	23,241.29	10,121.84	9414.956	12,227.13
	rank	1	11	7	12	2	13	10	8	3	9	5	4	6
C17-F10	mean	4347.157	12,501.61	8106.923	13,649.47	6421.64	11,350.83	11,358.13	7480.766	8426.742	13,449.81	8363.771	7602.505	11,284.14
	best	3555.132	11,986.21	7592.666	13,341.92	5583.099	10,422.06	10,135.81	6201.663	6491.304	12,737.64	7552.501	7404.352	10,755.79
	worst	5099.795	13,238.04	8574.312	14,051.99	7036.682	12,391.52	12,472.96	8513.43	13,314.41	13,992.03	9435.288	8097.764	11,957.84
	std	696.8528	648.0204	444.9295	348.1376	748.2319	920.4325	1107.857	1070.697	3548.154	691.959	849.0965	357.7925	557.7497
	median	4366.851	12,391.09	8130.356	13,601.99	6533.389	11,294.87	11,411.87	7603.986	6950.626	13,534.78	8233.648	7453.953	11,211.46
	rank	1	11	5	13	2	9	10	3	7	12	6	4	8
C17-F11	mean	1128.435	14,581.89	1578.784	19,859.3	1248.085	12,260.21	4870.902	1544.047	5845.583	4885.891	13455.6	1641.182	22712
	best	1121.25	13,439.55	1465.724	17,672.52	1202.111	10,546.11	4299.987	1401.329	3534.593	4585.473	12623.46	1383.954	13,300.14
	worst	1133.132	15,307.05	1722.936	21,518.66	1277.336	14,706.61	6080.301	1688.152	10,104.49	5431.524	15242.9	1948.663	30,437.42
	std	5.882766	892.6061	128.4513	1736.908	36.19834	1938.428	884.7653	134.8492	3279.846	420.9414	1301.835	261.2576	7660.041
	median	1129.678	14,790.49	1563.238	20,123.01	1256.447	11,894.07	4551.66	1543.353	4871.626	4763.284	12978.01	1616.056	23,555.22
	rank	1	11	4	12	2	9	6	3	8	7	10	5	13
C17-F12	mean	2905.102	4.12 × 10^10^	69,333,380	6.72 × 10^10^	13,605,050	2.44 × 10^10^	1.25 × 10^9^	74,845,249	9.05 × 10^8^	4.77 × 10^9^	2.05 × 10^9^	1.52 × 10^9^	1.93 × 10^8^
	best	2527.376	3.46 × 10^10^	29,368,335	4.9 × 10^10^	12,815,734	1.03 × 10^10^	1.03 × 10^9^	40,312,005	1.42 × 10^8^	2.69 × 10^9^	6.75 × 10^8^	11,998,079	60,941,738
	worst	3168.37	4.94 × 10^10^	1.07 × 10^8^	9.22 × 10^10^	14,242,998	4.11 × 10^10^	1.7 × 10^9^	1.19 × 10^8^	1.68 × 10^9^	9.39 × 10^9^	3.69 × 10^9^	4.38 × 10^9^	2.67 × 10^8^
	std	295.8235	7.22 × 10^9^	45,023,338	2.15 × 10^10^	720,098.7	1.38 × 10^10^	3.32 × 10^8^	35,795,915	8.3 × 10^8^	3.39 × 10^9^	1.35 × 10^9^	2.2 × 10^9^	98012316
	median	2962.331	4.04 × 10^10^	70,415,577	6.39 × 10^10^	13,680,735	2.32 × 10^10^	1.13 × 10^9^	69,985,918	8.98 × 10^8^	3.51 × 10^9^	1.92 × 10^9^	8.35 × 10^8^	2.22 × 10^8^
	rank	1	12	3	13	2	11	7	4	6	10	9	8	5
C17-F13	mean	1340.1	2.32 × 10^10^	140,897.8	4.07 × 10^10^	15,504.05	9.53 × 10^9^	89,692,261	228,031.4	3.38 × 10^8^	5.53 × 10^8^	17,510,759	4.51 × 10^8^	39,233,118
	best	1333.781	1.34 × 10^10^	32,451.57	2.06 × 10^10^	8237.933	5.06 × 10^9^	67,431,253	142,302.5	1.53 × 10^8^	4.51 × 10^8^	29,576.44	482,13.45	25,574,069
	worst	1343.015	3.17 × 10^10^	310,391.5	5.86 × 10^10^	18,238.2	1.48 × 10^10^	1.02 × 10^8^	355,794.9	8.49 × 10^8^	7.56 × 10^8^	59,025,621	1.14 × 10^9^	52,436,717
	std	4.628289	8.68 × 10^9^	128,454.8	1.72 × 10^10^	5240.914	4.47 × 10^9^	16,449,744	98,176.65	3.69 × 10^8^	1.49 × 10^8^	30,426,143	6 × 10^8^	12,965,594
	median	1341.801	2.39 × 10^10^	110,374.1	4.19 × 10^10^	17,770.04	9.11 × 10^9^	94,744,815	207,014.2	1.74 × 10^8^	5.03 × 10^8^	5,493,918	3.33 × 10^8^	39,460,843
	rank	1	12	3	13	2	11	7	4	8	10	5	9	6
C17-F14	mean	1429.458	24,547,842	1,156,499	45,767,518	1555.593	2,541,057	4,508,830	180,566.8	1,089,071	818,521.1	14,325,101	542,813	10,601,656
	best	1425.995	8,018,287	358,272.2	14,037,026	1542.966	671,287.8	3,991,939	114,408	84,867	674,927.6	3,247,926	195,033.9	5,216,894
	worst	1431.939	48,055,805	2,754,473	92,664,890	1578.888	4,030,294	5,358,038	350,420	2,101,517	944,373.3	23,520,465	869,340.2	18,246,361
	std	2.833096	18,261,458	1,177,220	36,148,276	17.75996	1,505,301	637,596.4	122,802.9	889,843.5	151,902	9,933,238	298,267.8	5,945,636
	median	1429.95	21,058,639	756,625.2	38,184,079	1550.258	2,731,324	4,342,672	128,719.6	1,084,951	827,391.7	15,266,006	553,439	9,471,685
	rank	1	12	7	13	2	8	9	3	6	5	11	4	10
C17-F15	mean	1530.66	2.46 × 10^9^	36,017.3	3.96 × 10^9^	2221.151	1.61 × 10^9^	9,383,591	114,874.9	5,626,635	66,737,994	1.87 × 10^8^	10,348.77	8,110,945
	best	1526.359	1.74 × 10^9^	22,265.22	3.09 × 10^9^	2095.201	5.54 × 10^8^	864,975.3	47,605.91	40,115.14	39,133,444	18,196.86	2707.533	2,756,255
	worst	1532.953	3.23 × 10^9^	66,256.73	4.69 × 10^9^	2360.307	3.51 × 10^9^	17,520,860	171,303.9	14,819,639	86,871,947	7.25 × 10^8^	20,257.53	17,603,160
	std	3.171095	7.54 × 10^8^	22,039.2	7.66 × 10^8^	151.7346	1.48 × 10^9^	7,912,741	59,427.02	6,970,083	21,577,152	3.88 × 10^8^	8430.859	7,096,598
	median	1531.664	2.44 × 10^9^	27,773.62	4.02 × 10^9^	2214.548	1.19 × 10^9^	9,574,265	120,294.8	3,823,394	70,473,293	11,294,126	92,15.01	6,042,184
	rank	1	12	4	13	2	11	8	5	6	9	10	3	7
C17-F16	mean	2062.891	6055.705	4230.866	7286.688	2715.947	4502.88	5307.482	3259.66	3256.914	4410.308	3853.938	3272.332	3814.265
	best	1728.6	5263.506	3906.274	5485.973	2565.283	3956.823	4366.29	3042.062	2885.687	4023.354	3533.835	2884.743	3220.098
	worst	2242.663	7712.695	4629.947	10,846.63	2972.475	4797.383	5941.226	3497.433	3815.358	4684.977	4243.311	3702.034	4321.48
	std	251.732	1242.726	370.6909	2648.976	207.9269	410.7924	747.5385	203.9334	487.8471	301.2195	372.8875	442.5776	518.7537
	median	2140.15	5623.309	4193.621	6407.076	2663.014	4628.656	5461.206	3249.573	3163.305	4466.451	3819.303	3251.275	3857.742
	rank	1	12	8	13	2	10	11	4	3	9	7	5	6
C17-F17	mean	2021.151	7318.249	3475.427	10565.46	2529.709	3852.955	4398.159	3013.855	2917.177	4034.141	3723.2	3279.233	3500.279
	best	1900.43	5587.931	3048.898	7733.522	2457.896	3105.043	3948.283	2486.807	2773.782	3424.845	3287.92	3067.102	3274.936
	worst	2138.267	8946.153	3980.719	13693.19	2586.617	4291.887	4614.205	3479.429	3181.295	4400.552	4018.469	3599.306	3731.108
	std	145.0735	1495.612	477.3995	2652.838	59.50525	559.1043	336.93	443.0295	195.5701	466.1732	342.289	271.9751	230.6331
	median	2022.954	7369.456	3436.045	10417.56	2537.161	4007.445	4515.074	3044.593	2856.815	4155.583	3793.205	3225.261	3497.537
	rank	1	12	6	13	2	9	11	4	3	10	8	5	7
C17-F18	mean	1830.62	71,644,933	2,282,053	1.06 × 10^8^	24,933.8	33,174,787	42,757,812	2,499,198	5,417,447	7,761,805	7,959,184	780,149.2	8,964,267
	best	1822.239	57,331,012	295,708.8	47,785,454	3639.966	2,982,075	11,579,835	1,472,444	1,032,978	5,338,148	3,763,256	332,571.7	3,212,137
	worst	1841.673	84,482,938	4,179,812	1.47 × 10^8^	37,239.95	94,779,822	77,398,257	3,890,401	10,806,504	10,789,188	14,873,844	1,279,249	21,549,980
	std	8.802698	12,676,296	2,127,114	52,960,169	15,864.59	45,596,982	35,182,593	1,250,349	5,511,839	2,492,407	5,474,104	469,263.6	9,154,274
	median	1829.285	72,382,892	2,326,346	1.15 × 10^8^	29,427.64	17,468,625	41,026,578	2,316,973	4,915,153	7,459,941	6,599,817	754,387.7	5,547,476
	rank	1	12	4	13	2	10	11	5	6	7	8	3	9
C17-F19	mean	1925.185	2.58 × 10^9^	245,908.8	3.63 × 10^9^	2073.532	2.53 × 10^9^	6,475,385	4,850,397	1,100,770	47,980,587	427,886.7	372,637.8	938,716.6
	best	1924.437	1.23 × 10^9^	86,374.43	2.45 × 10^9^	2015.54	9254734	974,023.2	3,692,101	538,842.2	40,733,792	246,096.2	2846.643	734,316.2
	worst	1926.121	4.3 × 10^9^	507,060.7	4.5 × 10^9^	2102.175	7.39 × 10^9^	15,261,932	6,015,265	1,692,437	60,929,130	937,548.3	930,588.7	1,271,577
	std	0.855282	1.4 × 10^9^	197,321.6	9.82 × 10^8^	42.77584	3.57 × 10^9^	6,635,893	1,025,132	521,283.7	9,716,370	367,361.1	478,110	274,030
	median	1925.091	2.39 × 10^9^	195,100.1	3.8 × 10^9^	2088.206	1.36 × 10^9^	4,832,792	4,847,111	1,085,900	45,129,714	263,951.2	278,557.9	874,486.6
	rank	1	12	3	13	2	11	9	8	7	10	5	4	6
C17-F20	mean	2160.172	3739.708	3206.236	3993.273	2632.62	3366.622	3665.426	3219.449	2598.753	3689.507	3941.403	3228.136	3113.444
	best	2104.423	3417.755	2647.545	3723.93	2361.815	2932.995	3379.457	2994.244	2404.456	3567.919	3676.154	2839.06	3047.135
	worst	2323.891	3908.591	3710.536	4160.335	2899.03	3574.769	4219.373	3661.5	2802.626	3850.492	4204.9	3393.829	3232.907
	std	117.9742	242.6596	492.3778	203.6536	245.8431	317.2803	414.1949	330.2313	222.9824	131.9757	234.3118	282.0011	89.87337
	median	2106.186	3816.244	3233.432	4044.414	2634.817	3479.362	3531.436	3111.026	2593.965	3669.808	3942.28	3339.828	3086.866
	rank	1	11	5	13	3	8	9	6	2	10	12	7	4
C17-F21	mean	2314.895	2958.552	2733.348	2995.17	2442.254	2925.395	2916.744	2560.302	2510.584	2796.451	2815.649	2641.299	2727.929
	best	2309.045	2923.947	2617.377	2895.165	2423.487	2823.876	2807.691	2526.766	2458.473	2773.337	2747.894	2572.718	2705.321
	worst	2329.683	2992.548	2912.56	3077.266	2465.361	3086.454	3007.585	2595.961	2551.222	2838.569	2852.73	2743.557	2745.95
	std	10.6856	36.84403	137.6229	93.90139	23.25965	122.2094	92.7019	38.81585	42.5424	32.71981	51.42597	81.54098	21.65185
	median	2310.426	2958.857	2701.727	3004.125	2440.085	2895.624	2925.85	2559.24	2516.321	2786.948	2830.986	2624.459	2730.223
	rank	1	12	7	13	2	11	10	4	3	8	9	5	6
C17-F22	mean	3095.169	14,381.28	10,735.22	15,586.33	5238.992	13,204.05	13,134.48	8696.273	8577.841	15,062.48	11,011.68	9417.496	8539.763
	best	2300	14,075.99	8477.805	15,337.64	2319.192	12,757.95	12,515.38	6902.035	7540.137	14,564.9	10,682.75	8607.223	3940.882
	worst	5480.678	14,614.08	12,338.27	15,901.64	8225.291	13,754.75	13,440.31	9900.313	9093.964	15,587.42	11,455.85	9873.749	13,014.37
	std	1718.838	252.9608	1986.881	297.0998	3431.247	461.0354	459.4987	1378.681	763.6749	524.5437	354.9343	643.2589	5437.789
	median	2300	14,417.51	11,062.41	15,553.02	5205.742	13,151.75	13,291.11	8991.372	8838.632	15,048.8	10,954.06	9594.506	8601.899
	rank	1	11	7	13	2	10	9	5	4	12	8	6	3
C17-F23	mean	2743.354	3773.165	3267.399	3845.773	2883.332	3699.586	3702.012	2978.122	3007.731	3257.733	4667.345	3349.089	3335.195
	best	2729.988	3697.11	3186.145	3800.165	2870.86	3497.727	3526.302	2937.802	2930.594	3172.86	4480.352	3284.399	3209.871
	worst	2752.657	3867.238	3345.548	3885.063	2902.648	4024.291	3799.342	3048.593	3141.995	3323.727	4832.166	3404.779	3468.512
	std	10.82585	80.48427	81.78723	38.4172	14.81681	271.1811	131.9987	56.41876	99.99404	67.63572	155.9564	69.12909	114.4869
	median	2745.387	3764.156	3268.952	3848.932	2879.909	3638.163	3741.202	2963.047	2979.168	3267.174	4678.432	3353.588	3331.199
	rank	1	11	6	12	2	9	10	3	4	5	13	8	7
C17-F24	mean	2919.043	4158.336	3489.092	4422.753	3059.509	3961.155	3793.065	3126.536	3187.581	3426.834	4322.995	3441.03	3634.107
	best	2909.046	3912.993	3382.367	3954.227	3030.994	3868.606	3686.068	3089.738	3092.641	3352.168	4289.373	3286.781	3595.241
	worst	2924.412	4706.895	3667.655	5570.329	3096.139	4095.636	3844.358	3160.497	3312.122	3483.627	4373.877	3591.621	3729.153
	std	7.375459	398.7304	133.6558	835.2546	31.62752	112.601	79.15459	33.14311	98.95744	66.27591	42.52424	146.7457	68.72469
	median	2921.358	4006.728	3453.172	4083.228	3055.451	3940.189	3820.917	3127.955	3172.78	3435.77	4314.364	3442.86	3606.016
	rank	1	11	7	13	2	10	9	3	4	5	12	6	8
C17-F25	mean	2983.145	8358.473	3169.846	11550.19	3064.409	5875.926	4102.998	3051.876	3987.845	4312.736	4220.542	3115.534	4001.888
	best	2980.235	6904.775	3142.826	9298.866	3044.6	4799.928	3711.576	3018.797	3799.736	3847.277	3887.124	3072.536	3898.905
	worst	2991.831	9278.402	3214.139	12932.72	3082.091	6899.094	4398.395	3070.37	4183.116	4880.249	4852.507	3162.809	4119.426
	std	6.258337	1137.135	33.26204	1845.364	16.73698	975.136	315.7771	25.35295	215.8708	563.6518	490.7048	49.57299	98.33715
	median	2980.257	8625.356	3161.209	11984.59	3065.473	5902.342	4151.01	3059.169	3984.264	4261.709	4071.269	3113.396	3994.61
	rank	1	12	5	13	3	11	8	2	6	10	9	4	7
C17-F26	mean	3776.432	13,660.32	10,678.55	14,603.91	3346.367	12,259.93	13,401.96	5707.421	6402.076	9477.971	11,225.99	7944.296	8773.46
	best	3748.807	13,432.6	10,187.45	14,009.68	3152.363	10,228.86	12,511.53	5236.83	6026.365	8701.664	10,888.84	7391.57	6977.404
	worst	3793.643	13,844.31	11,170.63	15,517.04	3624.726	13,476.25	15,057.43	5962.706	6754.967	10,201.56	11,612.76	8484.606	11,098.61
	std	21.02196	205.2241	434.607	707.3723	231.469	1523.052	1220.589	355.1918	410.8507	679.9345	326.2241	529.5801	2118.333
	median	3781.639	13,682.2	10,678.05	14,444.45	3304.189	12,667.3	13,019.43	5815.074	6413.485	9504.333	11,201.18	7950.503	8508.914
	rank	2	12	8	13	1	10	11	3	4	7	9	5	6
C17-F27	mean	3251.26	4734.4	3825.496	4915.84	3378.218	4648.455	4409.715	3357.69	3624.023	3806.748	7887.712	3629.276	4394.857
	best	3227.701	4428.353	3780.013	4554.826	3273.743	3963.317	3857.547	3318.37	3579.701	3620.846	7642.785	3375.58	4287.832
	worst	3313.631	4943.81	3887.409	5174.739	3474.196	5125.306	4961.724	3424.965	3671.332	3970.743	8232.623	3865.274	4534.067
	std	45.07966	245.282	53.15123	319.8348	88.96046	546.2171	561.1654	50.84626	51.51511	168.3518	307.5623	239.8878	112.9655
	median	3231.854	4782.718	3817.282	4966.898	3382.467	4752.599	4409.794	3343.713	3622.53	3817.701	7837.719	3638.124	4378.764
	rank	1	11	7	12	3	10	9	2	4	6	13	5	8
C17-F28	mean	3258.849	8498.986	3579.443	10,843.58	3348.462	7086.436	4756.321	3284.876	4355.288	5165.78	4984.769	3846.733	4965.869
	best	3258.849	7680.203	3500.359	9615.557	3313.193	5764.138	4172.184	3263.878	4095.464	4569.582	4926.445	3541.393	4722.747
	worst	3258.849	10,568.39	3665.74	14105.18	3391.773	8447.196	4979.887	3303.061	4681.937	5690.371	5099.843	4341.775	5145.847
	std	0	1503.213	88.19974	2354.415	41.65681	1470.184	422.2117	20.76792	295.661	497.7929	85.16528	374.3908	221.6401
	median	3258.849	7873.678	3575.837	9826.799	3344.442	7067.205	4936.606	3286.283	4321.875	5201.584	4956.394	3751.881	4997.441
	rank	1	12	4	13	3	11	7	2	6	10	9	5	8
C17-F29	mean	3263.038	13193.72	5410.664	18815.8	4060.692	6750.47	8803.392	4773.422	4809.39	6400.658	7974.326	4776.587	6030.774
	best	3247.132	8747.727	5271.406	10,011.71	3718.13	6321.401	5975.384	4344.624	4611.737	5532.581	6592.523	4554.931	5732.968
	worst	3278.787	18,067.57	5545.833	29,679.85	4295.145	7256.817	11,494.21	5348.985	5097.581	7352.586	10,413.84	4859.451	6612.477
	std	18.86722	4636.255	121.2592	9468.58	282.1155	419.2955	2456.69	454.5769	240.968	931.8459	1860.347	159.8595	445.044
	median	3263.116	12,979.79	5412.707	17,785.82	4114.748	6711.83	8871.987	4700.039	4764.122	6358.733	7445.468	4845.984	5888.826
	rank	1	12	6	13	2	9	11	3	5	8	10	4	7
C17-F30	mean	623,575.2	3.1 × 10^9^	20,745,165	5.2 × 10^9^	1,604,261	1.57 × 10^9^	1.5 × 10^8^	66,811,920	1.32 × 10^8^	2.85 × 10^8^	1.75 × 10^8^	4,592,435	55,400,197
	best	582,411.6	2.4 × 10^9^	12,686,412	3.19 × 10^9^	1,222,345	1.93 × 10^8^	1.02 × 10^8^	60,379,936	63,953,348	1.98 × 10^8^	1.34 × 10^8^	3,217,030	44,705,905
	worst	655,637.4	4.21 × 10^9^	28,422,580	8.17 × 10^9^	2,594,292	3.19 × 10^9^	2.07 × 10^8^	76,849,184	1.96 × 10^8^	3.6 × 10^8^	2.29 × 10^8^	6,374,175	77,753,600
	std	35,305.29	8.56 × 10^8^	8,355,125	2.32 × 10^9^	716,597.8	1.67 × 10^9^	57,361,836	7,724,811	72,107,272	73395175	43188598	1,687,509	16,536,183
	median	628,125.9	2.9 × 10^9^	20,935,834	4.73 × 10^9^	1,300,204	1.45 × 10^9^	1.46 × 10^8^	65,009,280	1.34 × 10^8^	2.9 × 10^8^	1.68 × 10^8^	4,389,268	49,570,641
	rank	1	12	4	13	2	11	8	6	7	10	9	3	5
Sum rank		30	335	166	367	63	294	269	112	144	248	254	150	207
Mean rank		1.03	11.6	5.72	12.7	2.17	10.1	9.28	3.86	4.97	8.55	8.76	5.17	7.14
Total rank		1	12	6	13	2	11	10	3	4	8	9	5	7

**Table 5:** Optimization results of CEC 2017 test suite (dimension = 100).

		KOA	WSO	AVOA	RSA	MPA	TSA	WOA	MVO	GWO	TLBO	GSA	PSO	GA
C17-F1	mean	100	1.58 × 10^11^	3.62 × 10^9^	2.2 × 10^11^	4.92 × 10^8^	1.19 × 10^11^	5.93 × 10^10^	62,253,430	5.4 × 10^10^	8.63 × 10^10^	1.29 × 10^11^	1.9 × 10^10^	5.31 × 10^10^
	best	100	1.54 × 10^11^	1.76 × 10^9^	2.17 × 10^11^	3.72 × 10^8^	1.05 × 10^11^	5.61 × 10^10^	51,873,540	4.68 × 10^10^	8.21 × 10^10^	1.19 × 10^11^	1.28 × 10^10^	5.02 × 10^10^
	worst	100	1.62 × 10^11^	5.21 × 10^9^	2.22 × 10^11^	6.21 × 10^8^	1.33 × 10^11^	6.64 × 10^10^	72,902,555	6.12 × 10^10^	9.51 × 10^10^	1.38 × 10^11^	2.58 × 10^10^	6 × 10^10^
	std	1.25 × 10^−14^	3.49 × 10^9^	1.53 × 10^9^	2.74 × 10^9^	1.3 × 10^8^	1.27 × 10^10^	5.16 × 10^9^	11,099,561	7.35 × 10^9^	6.46 × 10^9^	8.88 × 10^9^	7.73 × 10^9^	5.01 × 10^9^
	median	100	1.58 × 10^11^	3.76 × 10^9^	2.21 × 10^11^	4.87 × 10^8^	1.19 × 10^11^	5.74 × 10^10^	62,118,813	5.41 × 10^10^	8.4 × 10^10^	1.3 × 10^11^	1.86 × 10^10^	5.1 × 10^10^
	rank	1	12	4	13	3	10	8	2	7	9	11	5	6
C17-F3	mean	300	404,928	308,705.2	305,078.4	149,288.1	343,809.3	746,230.4	440,732.8	348,188.2	280,055.7	324,723.4	511,448.9	545,904.9
	best	300	368,992.5	301,489	294,284.2	114,279.6	275,513.4	653,126.4	366,098.8	318,613.1	262,693.3	300,559.7	387,456.7	523,506.8
	worst	300	423,506.9	315,620.7	311,426.4	180,638.3	392,636.1	864,185.5	527,615.9	381,299.2	296,333.9	355,410.5	717,963.1	563,654.1
	std	0	27,550.87	6455.142	8675.46	31,154.28	53,608.71	98,334.04	88,879.94	36,246.4	14,858.87	24,599.61	165,406	19,163.79
	median	300	413,606.4	308,855.5	307,301.5	151,117.3	353,543.8	733,804.8	434,608.2	346,420.3	280,597.9	321,461.8	470,188	548,229.4
	rank	1	9	5	4	2	7	13	10	8	3	6	11	12
C17-F4	mean	602.1722	42,138.29	1502.374	71,028.99	995.9106	15,163.2	10374.2	751.7312	4255.296	10,185.59	32,273.01	2370.108	8733.789
	best	592.0676	38,782.36	1266.555	64,387.23	889.1278	9932.833	8841.988	699.3299	3275.684	9707.125	25,669.07	1446.025	8255.076
	worst	612.2769	46,196.97	1651.894	79,138.29	1106.866	20,151.17	11,387.64	808.4239	6380.153	11,011.91	36,518.9	2982.036	9277.937
	std	12.61058	3445.179	189.1307	6610.851	113.7622	4563.887	1170.386	49.21291	1545.055	670.7662	5662.855	715.5603	514.181
	median	602.1722	41,786.92	1545.524	70,295.22	993.8243	15284.4	10,633.58	749.5855	3682.673	10,011.66	33,452.04	2526.186	8701.071
	rank	1	12	4	13	3	10	9	2	6	8	11	5	7
C17-F5	mean	512.9345	1875.442	1245.443	1846.994	1162.661	2018.132	1732.529	1172.417	1123.255	1765.013	1265.907	1338.14	1494.762
	best	510.9445	1857.699	1234.193	1813.979	1044	1994.674	1641.963	1070.597	1070.278	1739.553	1234.13	1245.053	1358.062
	worst	514.9244	1886.314	1254.082	1879.377	1242.711	2045.421	1874.127	1237.929	1168.535	1792.377	1295.293	1499.148	1576.841
	std	1.963315	13.38656	9.021254	35.65558	103.5764	25.30619	108.9261	81.39582	46.37727	23.34161	34.43228	128.972	106.3335
	median	512.9345	1878.878	1246.749	1847.309	1181.966	2016.216	1707.014	1190.572	1127.102	1764.06	1267.103	1304.179	1522.073
	rank	1	12	5	11	3	13	9	4	2	10	6	7	8
C17-F6	mean	600	697.8946	656.8698	696.2964	634.3171	702.0988	695.5838	668.6175	636.9566	674.7253	658.8237	656.4638	657.9726
	best	600	695.399	653.1221	691.7436	630.7892	690.7201	686.5543	662.3883	632.3377	666.5929	656.4249	649.8042	651.1735
	worst	600	700.2302	660.7982	698.9958	640.2906	709.9386	711.556	674.4143	642.7928	679.6217	662.7141	661.8933	663.1022
	std	0	2.356801	3.419722	3.464414	4.86788	10.02518	12.12875	5.583037	4.901089	6.734223	2.998644	6.206345	6.49099
	median	600	697.9746	656.7795	697.2231	633.0944	703.8682	692.1125	668.8336	636.3479	676.3432	658.0778	657.0789	658.8074
	rank	1	12	5	11	2	13	10	8	3	9	7	4	6
C17-F7	mean	811.392	3384.456	2895.673	3491.538	1755.078	3223.741	3357.825	1906.897	1921.165	2910.907	2934.314	2338.869	2429.204
	best	810.0205	3303.584	2747.83	3407.135	1700.904	3057.858	3245.78	1756.368	1745.893	2776.807	2812.989	2091.564	2336.126
	worst	813.1726	3479.205	3019.769	3563.237	1830.313	3380.477	3520.905	2021.001	2050.532	3021.071	3135.055	2449.042	2632.894
	std	1.579207	77.97248	146.985	72.46239	60.45772	157.4048	136.5337	119.0395	137.7656	109.0443	151.8379	183.0957	148.6763
	median	811.1874	3377.518	2907.546	3497.891	1744.548	3228.315	3332.307	1925.109	1944.117	2922.875	2894.607	2407.434	2373.898
	rank	1	12	7	13	2	10	11	3	4	8	9	5	6
C17-F8	mean	812.437	2286.799	1659.732	2337.135	1378.597	2265.896	2192.315	1400.374	1456.862	2132.372	1740.834	1630.792	1929.733
	best	808.9546	2239.937	1608.077	2314.305	1220.571	2202.749	2006.981	1259.576	1359.291	2072.598	1664.978	1592.233	1882.246
	worst	816.9143	2342.537	1685.519	2351.528	1476.704	2346.692	2334.862	1568.602	1586.323	2181.048	1861.416	1718.649	1977.445
	std	3.673025	47.51148	38.48614	17.31721	121.5195	74.94211	181.4813	137.8975	110.5319	50.88975	94.97685	63.7038	43.93465
	median	811.9395	2282.361	1672.667	2341.353	1408.557	2257.071	2213.708	1386.66	1440.917	2137.922	1718.471	1606.142	1929.621
	rank	1	12	6	13	2	11	10	3	4	9	7	5	8
C17-F9	mean	900	82,357.33	24,238.04	70,609.31	20534.9	110,065.6	70,164.92	54,136.77	32,881.11	68,027.74	21,571.53	30,064.46	42,041.39
	best	900	73,524.29	20,181.86	68,255.4	19,115.08	90,235.06	54,566.97	45,653.35	20,357.27	65,153.88	20,080.31	25,427.36	38,078.22
	worst	900	95,138.23	27,278.31	72,536.97	21,177.59	137,283.4	88,423.61	61,570.09	44,704.04	69,555.68	22,726.29	33,483.09	47,354.09
	std	1 × 10^−13^	10,079.51	3196.155	2016.478	1031.208	21,350.02	18,271.2	7083.24	12,846.03	2171.179	1195.911	3871.169	4210.184
	median	900	80,383.4	24,746.01	70,822.43	20,923.46	106,372.1	68,834.55	54,661.83	33,231.56	68,700.7	21,739.76	30673.7	41,366.63
	rank	1	12	4	11	2	13	10	8	6	9	3	5	7
C17-F10	mean	11,023.04	28,764.18	15,559.65	29,986.93	13,634.91	27,925.44	26,965.42	16,501.04	14,842.15	29,995.83	16,716.58	16,576.16	24,908.94
	best	9625.608	28,501.48	13,149.25	29,169.72	12,988.72	27,288.18	26,175.72	15,914.27	13,748.85	28,785.02	15,044.3	14,929.99	24,375.93
	worst	11,858.81	29,077.7	17,675.53	30,462.85	14,449.59	28,802.58	28,299.11	17,067.07	15,395.76	31,002.21	17,671.53	17718.83	25,453.71
	std	1047.15	280.1557	2147.914	640.6182	673.5145	744.1283	1034.361	532.7258	812.0447	1002.222	1299.163	1274.408	476.2344
	median	11,303.87	28,738.77	15,706.92	30,157.57	13,550.67	27,805.5	26,693.43	16,511.41	15,112	30,098.05	17,075.24	16,827.92	24,903.06
	rank	1	11	4	12	2	10	9	5	3	13	7	6	8
C17-F11	mean	1162.329	152,618.7	59,526.88	191,511	4526.662	60,681.68	193,293.4	4339.101	80,911.4	66,616.12	160,253.9	48,336.21	129,219.1
	best	1139.568	118,460.4	53,490.28	146,528.4	3580.408	27,691.6	112,503.9	3785.463	67,217	56,196.42	133,547.3	22,017.74	98,621.27
	worst	1220.662	177,615.2	71,119.92	272,854.4	5398.409	86,791.06	311,624.6	4595.548	91,165.53	84,913.49	186,954.5	98,740.3	178,142.1
	std	42.18991	27,488.35	8816.241	61,513.04	845.6541	26,477.2	100,114.6	403.6663	11,075.45	13,609.17	23,840.8	37,083.83	37,651.49
	median	1144.542	157,199.6	56,748.65	173,330.6	4563.915	64,122.04	174,522.6	4487.696	82631.53	62,677.29	160,256.9	36,293.39	120,056.5
	rank	1	10	5	12	3	6	13	2	8	7	11	4	9
C17-F12	mean	5974.805	9.79 × 10^10^	6.11 × 10^8^	1.59 × 10^11^	2.42 × 10^8^	5.27 × 10^10^	1.22 × 10^10^	3.08 × 10^8^	1.06 × 10^10^	2.03 × 10^10^	6.2 × 10^10^	9.36 × 10^9^	1.14 × 10^10^
	best	5383.905	6.95 × 10^10^	3.24 × 10^8^	1.19 × 10^11^	1.35 × 10^8^	2.7 × 10^10^	9.93 × 10^9^	1.96 × 10^8^	7.35 × 10^9^	1.6 × 10^10^	5.37 × 10^10^	1.22 × 10^9^	1.04 × 10^10^
	worst	6570.199	1.09 × 10^11^	9.75 × 10^8^	1.85 × 10^11^	2.9 × 10^8^	8.74 × 10^10^	1.4 × 10^10^	4.84 × 10^8^	1.26 × 10^10^	2.8 × 10^10^	7.29 × 10^10^	1.78 × 10^10^	1.35 × 10^10^
	std	534.4265	2.05 × 10^10^	3.04 × 10^8^	3.27 × 10^10^	77639019	2.73 × 10^10^	1.84 × 10^9^	1.37 × 10^8^	2.46 × 10^9^	5.95 × 10^9^	8.62 × 10^9^	8.16 × 10^9^	1.52 × 10^9^
	median	5972.559	1.06 × 10^11^	5.72 × 10^8^	1.66 × 10^11^	2.71 × 10^8^	4.82 × 10^10^	1.25 × 10^10^	2.77 × 10^8^	1.12 × 10^10^	1.87 × 10^10^	6.06 × 10^10^	9.22 × 10^9^	1.09 × 10^10^
	rank	1	12	4	13	2	10	8	3	6	9	11	5	7
C17-F13	mean	1407.28	2.58 × 10^10^	91,256.28	3.96 × 10^10^	90,004.15	1.98 × 10^10^	4.85 × 10^8^	328,704.5	8.79 × 10^8^	2.61 × 10^9^	8.1 × 10^9^	1.64 × 10^9^	1.62 × 10^8^
	best	1371.145	2.25 × 10^10^	64,482.87	3.06 × 10^10^	38,600.78	1.41 × 10^10^	3.45 × 10^8^	289,666.1	75804760	1.81 × 10^9^	4.98 × 10^9^	1.8 × 10^8^	1.27 × 10^8^
	worst	1439.935	2.87 × 10^10^	124,386.1	4.49 × 10^10^	223,420.4	2.37 × 10^10^	6.56 × 10^8^	383,212.3	2.32 × 10^9^	3.16 × 10^9^	1.04 × 10^10^	2.96 × 10^9^	1.95 × 10^8^
	std	37.55799	3.47 × 10^9^	27,453.11	7.12 × 10^9^	96,700.25	4.42 × 10^9^	1.73 × 10^8^	44,258.34	1.12 × 10^9^	6.68 × 10^8^	2.45 × 10^9^	1.48 × 10^9^	38092155
	median	1409.02	2.61 × 10^10^	88,078.07	4.15 × 10^10^	48,997.69	2.07 × 10^10^	4.7 × 10^8^	320,969.8	5.58 × 10^8^	2.74 × 10^9^	8.51 × 10^9^	1.7 × 10^9^	1.63 × 10^8^
	rank	1	12	3	13	2	11	6	4	7	9	10	8	5
C17-F14	mean	1467.509	42,216,311	6,204,107	74,060,256	84,566.61	8,269,863	13,526,119	2,820,503	8,941,375	12,930,337	10,688,997	757,803.5	9,764,305
	best	1458.803	36,457,596	3,762,266	67,546,820	24,206.95	3,756,391	7,786,344	851,265	5,655,602	9,633,942	8,238,887	360,174.1	5,463,372
	worst	1472.733	48,225,605	10,299,057	81,072,680	179,579.5	16,135,326	18,489,581	3,882,368	13,404,428	16,523,669	16,030,636	1,573,345	14,383,968
	std	6.533884	5,586,786	3,112,254	7,022,252	75,198.33	5,894,917	4,756,293	1,460,448	3,671,034	3,893,637	3,892,590	596,855.2	4,013,341
	median	1469.25	42,091,021	5,377,553	73,810,762	67,240.01	6,593,868	13,914,276	3,274,190	8,352,736	12,781,869	9,243,232	548,847.3	9,604,939
	rank	1	12	5	13	2	6	11	4	7	10	9	3	8
C17-F15	mean	1609.893	1.43 × 10^10^	78,513.4	2.19 × 10^10^	52,157.64	1.12 × 10^10^	65,169,852	117,705.4	4.66 × 10^8^	1.11 × 10^9^	1.15 × 10^9^	3.1 × 10^8^	11,796,816
	best	1551.154	1.32 × 10^10^	64,221.08	1.56 × 10^10^	15,118.62	2.33 × 10^8^	36,296,713	80,566.26	3,057,2171	3.7 × 10^8^	4.62 × 10^8^	57,252.33	7,606,569
	worst	1652.294	1.61 × 10^10^	98,545.34	2.73 × 10^10^	79,155.02	2.1 × 10^10^	1.25 × 10^8^	173,167.1	1.4 × 10^9^	2.37 × 10^9^	1.48 × 10^9^	1.22 × 10^9^	20,100,543
	std	47.73046	1.34 × 10^9^	17,735.99	6.24 × 10^9^	29,217.94	9.74 × 10^9^	43,882,701	44,064.82	6.83 × 10^8^	9.46 × 10^8^	5.07 × 10^8^	6.59 × 10^8^	6,134,076
	median	1618.063	1.4 × 10^10^	75,643.59	2.23 × 10^10^	57,178.46	1.18 × 10^10^	49,580,925	108,544.1	2.19 × 10^8^	8.48 × 10^8^	1.34 × 10^9^	8,025,672	9,740,076
	rank	1	12	3	13	2	11	6	4	8	9	10	7	5
C17-F16	mean	2711.795	17,807.45	6829.288	21,253.99	5332.392	13,739.07	15,293.69	6329.66	5869.532	10,884.72	10,477.1	6225.534	9999.215
	best	2171.69	16598	5742.525	16,745.04	5239.684	11,341.61	12,487.67	5625.82	5308.174	10,390.05	9089.455	5978.097	9046.099
	worst	3397.326	18,341.03	7514.044	23745.9	5460.054	16,458.29	16,919.1	6794.504	6497.975	11,891.14	12,089.74	6424.768	10,750.83
	std	551.162	879.6119	831.8722	3433.857	106.0604	2272.515	2146.317	563.4107	667.5113	764.3616	1452.409	199.4727	836.6546
	median	2639.081	18,145.39	7030.292	22,262.51	5314.915	13,578.19	15,884	6449.159	5835.99	10,628.84	10,364.6	6249.636	10,099.97
	rank	1	12	6	13	2	10	11	5	3	9	8	4	7
C17-F17	mean	2716.564	3,927,275	5624.724	7,725,893	4511.263	203,733.2	16042.01	4809.563	5308.937	8323.727	43,381.69	5860.353	6845.326
	best	2275.021	1,151,113	5414.129	2,094,250	4288.731	9678.96	9909.52	4382.975	4306.933	8195.078	28,508.01	5606.956	6686.242
	worst	3429.127	8,935,000	6060.028	17,777,316	4710.347	540,996.5	27,054.55	5129.665	6853.048	8495.943	70,408.53	6081.679	7005.675
	std	556.02	3,965,848	328.0954	7,974,652	229.3653	250,918.8	8340.432	407.3598	1220.069	157.2312	20,046.65	216.3887	143.4659
	median	2581.054	2,811,493	5512.37	5,516,003	4522.987	132,128.7	13,601.98	4862.807	5037.884	8301.944	37,305.12	5876.389	6844.694
	rank	1	12	5	13	2	11	9	3	4	8	10	6	7
C17-F18	mean	1903.746	54,356,713	2,621,689	95,922,318	216,127.9	13,870,673	11,171,762	4,568,794	10,201,355	15,082,925	10,942,616	5,991,480	5,620,042
	best	1881.15	24,625,693	1,303,737	37,231,272	150,714.8	5,195,488	8,309,688	3,383,599	3,212,533	11,114,282	5,044,896	3,700,303	4,506,220
	worst	1919.921	98,298,865	4,144,976	1.75 × 10^8^	389,411.7	28,344,410	13,234,559	7,674,482	16,485,084	21,319,750	24,326,599	8,631,796	8,136,061
	std	20.94507	34,014,380	1,391,324	62,946,116	125,243.3	11,273,064	2,425,796	2,246,539	5,902,257	4,738,024	9,823,889	2,476,657	1,846,375
	median	1906.955	47,251,148	2,519,022	85,531,102	162,192.5	10,971,396	11,571,401	3,608,547	10,553,901	13,948,834	7,199,485	5,816,911	4,918,944
	rank	1	12	3	13	2	10	9	4	7	11	8	6	5
C17-F19	mean	1972.839	1.18 × 10^10^	2,680,996	2.08 × 10^10^	260,890.9	4.7 × 10^9^	1.25 × 10^8^	15,497,056	3.36 × 10^8^	6.23 × 10^8^	1.47 × 10^9^	2.51 × 10^8^	11,920,249
	best	1967.139	1.04 × 10^10^	1,026,475	1.52 × 10^10^	54,987	2.08 × 10^9^	49,517,164	9,038,752	2,665,277	2.7 × 10^8^	2.65 × 10^8^	41735153	6,085,280
	worst	1977.869	1.39 × 10^10^	4,935,230	2.59 × 10^10^	441,843	9.34 × 10^9^	2.1 × 10^8^	24,632,296	1.01 × 10^9^	1.43 × 10^9^	2.78 × 10^9^	5.43 × 10^8^	21,558,115
	std	4.903424	1.7 × 10^9^	1,785,587	4.78 × 10^9^	173,544.5	3.47 × 10^9^	80,527,497	8,319,908	5.08 × 10^8^	5.9 × 10^8^	1.35 × 10^9^	2.63 × 10^8^	7,420,795
	median	1973.174	1.15 × 10^10^	2,381,140	2.11 × 10^10^	273,366.8	3.69 × 10^9^	1.2 × 10^8^	14,158,588	1.65 × 10^8^	3.94 × 10^8^	1.42 × 10^9^	2.1 × 10^8^	10,018,800
	rank	1	12	3	13	2	11	6	5	8	9	10	7	4
C17-F20	mean	3192.04	7023.285	5985.235	7260.025	4412.931	6781.841	6793.6	5644.431	5890.077	6984.874	6120.649	5233.459	6075.159
	best	2806.762	6829.743	5654.233	7149.763	4348.395	6182.804	6387.46	5349.746	4727.223	6213.519	5707.013	4530.691	5488.476
	worst	3662.121	7208.922	6244.551	7348.729	4462.415	7523.552	7159.203	6141.253	6760.14	7304.646	6356.099	6062.992	6518.798
	std	474.8604	174.0937	305.793	89.29151	54.68408	625.1056	363.8662	371.3965	1086.402	559.1247	314.4945	708.369	532.1535
	median	3149.639	7027.237	6021.078	7270.804	4420.457	6710.503	6813.868	5543.363	6036.472	7210.666	6209.742	5170.075	6146.681
	rank	1	12	6	13	2	9	10	4	5	11	8	3	7
C17-F21	mean	2342.155	4151.348	3574.821	4266.647	2799.036	3997.715	4093.548	3175.182	2932.013	3613.086	4549.153	3496.065	3343.183
	best	2338.689	4107.393	3376.278	4194.602	2757.144	3862.258	3811.848	3110.225	2854.849	3461.704	4028.685	3319.511	3309.565
	worst	2346.015	4217.579	3704.394	4320.067	2831.742	4089.581	4311.231	3297.735	2982.826	3785.023	4967.745	3829.46	3390.042
	std	3.641031	56.93501	152.9064	58.93068	34.17408	120.6932	241.6377	90.93975	58.8322	148.8337	423.2554	249.8118	37.59003
	median	2341.959	4140.209	3609.306	4275.959	2803.629	4019.511	4125.556	3146.383	2945.189	3602.808	4600.09	3417.645	3336.562
	rank	1	11	7	12	2	9	10	4	3	8	13	6	5
C17-F22	mean	11,739	30,389.97	19,679.11	31,939.77	18,226.43	29,450.95	27,924.04	16,910.76	22,548.19	31,825.23	20,522.33	21,234.75	27,607.99
	best	11,119.08	29,591.74	18,382.78	31,596.57	16,981.68	28,324.93	26,479.85	15,962.66	18,088.1	30,868.7	19,828.32	19,895.94	26,619.11
	worst	12,601.6	30,838.34	21,415.94	32,523.91	19,791.77	30,521.39	29,094.01	17,579.6	32,990.52	32,297.18	20,865.15	22,720.8	28,365.86
	std	705.4602	624.8952	1474.099	465.5068	1281.164	971.7317	1246.104	857.364	7647.385	700.4176	508.5205	1275.296	909.2197
	median	11,617.67	30,564.91	19,458.85	31,819.29	18,066.14	29,478.73	28,061.15	17,050.39	19,557.07	32,067.52	20,697.92	21,161.13	27,723.5
	rank	1	11	4	13	3	10	9	2	7	12	5	6	8
C17-F23	mean	2877.697	5183.395	4035.008	5185.429	3271.212	5296.835	5008.148	3447.223	3572.999	4129.891	7584.361	4745.825	4179.074
	best	2872.107	4945.115	3958.545	4931.657	3256.419	4577.595	4872.533	3360.159	3542.184	4079.205	7020.19	4252.255	4115.922
	worst	2884.013	5462.627	4115.774	5384.701	3300.867	6271.779	5144.648	3559.774	3616.474	4204.22	7983.468	5007.602	4240.551
	std	5.637338	250.2595	80.11963	202.7715	21.6953	819.3767	140.7254	91.22045	36.74315	57.3217	470.63	367.588	73.25936
	median	2877.334	5162.918	4032.856	5212.679	3263.78	5168.984	5007.706	3434.48	3566.67	4118.07	7666.893	4861.722	4179.912
	rank	1	10	5	11	2	12	9	3	4	6	13	8	7
C17-F24	mean	3327.407	8237.866	5258.389	10,101.14	3694.095	6484.728	6209.392	3932.62	4240.506	4672.899	10,398.02	5813.699	5261.184
	best	3295.518	6456.699	5051.152	6815.066	3649.127	6017.893	5808.354	3866.027	4014.79	4451.077	9777.358	5457.958	5175.657
	worst	3357.991	9447.59	5431.181	12,287.15	3757.133	6792.878	6818.978	4037.589	4443.96	4891.541	12026.38	6267.152	5422.226
	std	32.01326	1546.525	182.1746	2862.73	55.89092	357.0162	475.2686	87.21459	239.8235	195.1663	1174.871	390.3473	121.0442
	median	3328.059	8523.587	5275.612	10,651.17	3685.059	6564.071	6105.117	3913.432	4251.637	4674.488	9894.175	5764.842	5223.427
	rank	1	11	6	12	2	10	9	3	4	5	13	8	7
C17-F25	mean	3185.232	14,590.42	4086.698	20,310.76	3658.19	10,067.47	7074.01	3396.171	6250.883	8588.452	10594.16	4087.701	7625.964
	best	3137.371	13,878.09	3727.323	18,843.71	3489.381	9445.538	6483.569	3333.134	6101.685	7418.938	9782.467	3832.002	6948.964
	worst	3261.571	16,259.31	4423.181	23,589.11	3778.549	10,467.63	7437.697	3461.629	6633.122	10,150.83	12,040.09	4493.102	8316.299
	std	64.74694	1215.09	310.2059	2414.639	131.1693	501.7799	465.92	58.5326	276.6495	1350.275	1080.962	342.0666	767.1223
	median	3170.992	14,112.14	4098.144	19,405.11	3682.415	10178.35	7187.386	3394.961	6134.363	8392.017	10277.05	4012.85	7619.298
	rank	1	12	4	13	3	10	7	2	6	9	11	5	8
C17-F26	mean	5757.621	37,599.89	23,572.79	43,207.23	11,303.81	31,755.76	32,347.71	11,500.89	16,242.84	22,847.97	32,255.29	19,867.5	22,032.2
	best	5645.905	37,074.74	20,870.36	40,786.27	10,621.25	30,564.12	29,032.47	10,208.24	14,452.99	18,739.45	30,934.73	17,821.34	20,493.21
	worst	5844.642	38,074.5	26,341.57	44706.3	12,019.7	32,501.94	35,155.84	13,765.58	17,757.37	28,060.05	33,983.27	21,759.69	23,073.9
	std	90.69965	451.3916	2529.672	2034.296	747.0293	902.2386	3268.805	1686.672	1505.917	4178.487	1383.563	1793.502	1201.027
	median	5769.969	37,625.15	23,539.62	43,668.18	11,287.15	31,978.48	32,601.27	11,014.87	16,380.51	22,296.19	32,051.58	19,944.49	22,280.85
	rank	1	12	8	13	2	9	11	3	4	7	10	5	6
C17-F27	mean	3309.493	9004.867	4118.41	11805.97	3522.956	6429.837	5864.661	3607.936	4041.885	4275.759	13479.04	4034.66	5372.113
	best	3278.01	7603.881	3953.301	8878.405	3486.441	6143.785	5196.528	3568.344	3879.077	4011.16	13152.01	3840.815	5123.343
	worst	3344.5	10,418.38	4391.013	14849.6	3554.811	6785.19	6607.717	3699.249	4170.417	4711.741	13739.86	4228.793	5747.071
	std	30.65647	1653.48	204.4777	3479.24	30.3614	300.5311	822.9413	66.8764	155.0178	336.4504	287.0312	231.2728	288.6769
	median	3307.732	8998.604	4064.662	11747.94	3525.287	6395.185	5827.2	3582.075	4059.022	4190.067	13512.14	4034.515	5309.019
	rank	1	11	6	12	2	10	9	3	5	7	13	4	8
C17-F28	mean	3322.242	19,970.48	4634.162	26,913.35	3747.849	15,045.04	10,001.47	3451.906	8955.999	10,757.53	17,980.34	7416.474	11,054.79
	best	3318.742	18,603.57	4346.486	24,122.95	3629.68	11,836.61	8565.014	3371.964	7606.689	8432.049	15,531.52	5079.455	10,078.92
	worst	3327.816	22,500.95	4846.292	30,405.94	3832.176	17,476.75	10942.3	3530.021	10,881.4	12,798.86	19,827.58	11,380.86	12,136.52
	std	4.736062	1916.19	228.6993	2847.832	92.0496	2921.811	1093.816	70.30743	1492.568	2199.987	1942.042	3103.737	1190.622
	median	3321.205	19,388.7	4671.935	26,562.25	3764.769	15,433.4	10,249.28	3452.819	8667.953	10,899.6	18,281.13	6602.789	11,001.85
	rank	1	12	4	13	3	10	7	2	6	8	11	5	9
C17-F29	mean	4450.696	173,640.1	9332.285	330,325.3	6743.429	17,673.7	15876.58	8443.804	8086.701	11,981.54	23,809.95	8409.62	11,421.28
	best	4169.151	99,002.72	8115.408	177,338.5	5954.734	13,612.53	13,266.43	7567.553	7913.373	11,160.53	19,685.19	7779.584	11,224.67
	worst	4829.521	236,869.2	10,049.58	458,501.8	7479.424	22,334.88	18165	9053.309	8377.159	12,560.52	31,169.25	9253.719	11,868
	std	305.1554	63,448.63	910.0788	129,502.2	675.575	3932.541	2606.5	696.2608	223.4335	643.3947	5778.63	752.3304	326.3862
	median	4402.056	179,344.2	9582.073	342,730.4	6769.779	17,373.69	16,037.45	8577.176	8028.136	12,102.54	22,192.68	8302.588	11,296.22
	rank	1	12	6	13	2	10	9	5	3	8	11	4	7
C17-F30	mean	5407.166	2.18 × 10^10^	26,142,846	3.56 × 10^10^	4,427,168	1.26 × 10^10^	1.41 × 10^9^	97,057,830	1.73 × 10^9^	3.57 × 10^9^	6.93 × 10^9^	5.7 × 10^8^	6.28 × 10^8^
	best	5337.48	1.92 × 10^10^	14,897,444	3.32 × 10^10^	1,972,928	7.69 × 10^9^	1.16 × 10^9^	59,721,612	7.11 × 10^8^	1.34 × 10^9^	4.94 × 10^9^	1.39 × 10^8^	5.24 × 10^8^
	worst	5557.155	2.38 × 10^10^	45,972,422	3.84 × 10^10^	7,228,943	1.56 × 10^10^	1.92 × 10^9^	1.19 × 10^8^	2.26 × 10^9^	6.62 × 10^9^	8.39 × 10^9^	1.77 × 10^9^	6.73 × 10^8^
	std	109.3306	2.08 × 10^9^	15,036,707	2.43 × 10^9^	2,625,311	3.76 × 10^9^	3.69 × 10^8^	28729772	7.55 × 10^8^	2.86 × 10^9^	1.57 × 10^9^	8.64 × 10^8^	75569225
	median	5367.014	2.22 × 10^10^	21,850,759	3.53 × 10^10^	4,253,401	1.36 × 10^10^	1.29 × 10^9^	1.05 × 10^8^	1.98 × 10^9^	3.16 × 10^9^	7.19 × 10^9^	1.88 × 10^8^	6.57 × 10^8^
	rank	1	12	3	13	2	11	7	4	8	9	10	5	6
Sum rank		29	336	140	355	65	293	265	114	156	249	272	162	203
Mean rank		1.00	11.6	4.83	12.2	2.24	10.1	9.14	3.93	5.38	8.59	9.38	5.59	7.00
Total rank		1	12	4	13	2	11	9	3	5	8	10	6	7

**Table 6:** Wilcoxon rank sum test results.

Compared Algorithm	Objective Function Type			
	CEC 2017			
	D = 10	D = 30	D = 50	D = 100
KOA vs. WSO	2.02 × 10^−21^	1.97 × 10^−21^	1.97 × 10^−21^	1.97 × 10^−21^
KOA vs. AVOA	3.77 × 10^−19^	3.02 × 10^−21^	1.97 × 10^−21^	1.97 × 10^−21^
KOA vs. RSA	1.97 × 10^−21^	1.97 × 10^−21^	1.97 × 10^−21^	1.97 × 10^−21^
KOA vs. MPA	2 × 10^−18^	1.56 × 10^−16^	6.62 × 10^−18^	1.97 × 10^−21^
KOA vs. TSA	9.5 × 10^−21^	1.97 × 10^−21^	1.97 × 10^−21^	1.97 × 10^−21^
KOA vs. WOA	9.5 × 10^−21^	1.97 × 10^−21^	1.97 × 10^−21^	1.97 × 10^−21^
KOA vs. MVO	9.03 × 10^−19^	2.13 × 10^−21^	1.97 × 10^−21^	1.97 × 10^−21^
KOA vs. GWO	5.23 × 10^−21^	1.97 × 10^−21^	1.97 × 10^−21^	1.97 × 10^−21^
KOA vs. TLBO	3.69 × 10^−21^	1.97 × 10^−21^	1.97 × 10^−21^	1.97 × 10^−21^
KOA vs. GSA	1.6 × 10^−18^	2.02 × 10^−21^	1.97 × 10^−21^	1.97 × 10^−21^
KOA vs. PSO	1.54 × 10^−19^	2.35 × 10^−21^	1.97 × 10^−21^	1.97 × 10^−21^
KOA vs. GA	2.71 × 10^−19^	1.97 × 10^−21^	1.97 × 10^−21^	1.97 × 10^−21^

**Table 7:** Optimization results of the CEC 2011 test suite.

		KOA	WSO	AVOA	RSA	MPA	TSA	WOA	MVO	GWO	TLBO	GSA	PSO	GA
C11-F1	mean	5.920103	17.69621	12.96467	21.97703	7.568894	18.42799	13.25656	14.01025	10.86648	18.45995	21.6979	17.96646	23.39238
	best	2E-10	15.32282	8.843037	20.11259	0.371001	17.49967	8.211643	11.67231	1.113002	17.0621	19.58887	10.44374	22.24898
	worst	12.30606	20.53782	16.90606	24.49662	12.68518	19.88034	17.35422	16.01316	17.36624	19.9275	23.28124	24.29279	25.59286
	std	7.399774	2.789025	4.832981	2.313631	6.116142	1.131403	4.572836	2.388968	7.500419	1.265393	1.662208	6.896339	1.616415
	median	5.687176	17.4621	13.05478	21.64946	8.609699	18.16597	13.7302	14.17777	12.49333	18.42511	21.96075	18.56466	22.86384
	rank	1	7	4	12	2	9	5	6	3	10	11	8	13
C11-F2	mean	−26.3179	−14.5099	−21.0989	−11.7212	−25.1038	−11.4432	−18.702	-8.98275	−22.6755	−11.0541	−15.6523	−22.7239	−13.0666
	best	−27.0676	−15.8192	−21.6498	−12.1497	−25.7333	−15.1217	−22.0626	−10.9937	−24.7335	−12.251	−20.672	−24.0624	−15.3528
	worst	−25.4328	−13.3261	−20.3939	−11.2765	−23.7963	−9.28114	−14.7666	−7.46542	−19.1239	−9.99702	−11.6214	−20.3886	−11.4145
	std	0.75982	1.374082	0.593766	0.518106	0.977426	2.981024	4.069634	1.659995	2.68147	1.011466	4.444806	1.736414	2.007767
	median	−26.3856	−14.4472	−21.176	−11.7292	−25.4427	−10.6851	−18.9893	−8.73593	−23.4223	−10.9842	−15.1579	−23.2224	−12.7496
	rank	1	8	5	10	2	11	6	13	4	12	7	3	9
C11-F4	mean	1.15 × 10^−5^	1.15 × 10^−5^	1.15 × 10^−5^	1.15 × 10^−5^	1.15 × 10^−5^	1.15 × 10^−5^	1.15 × 10^−5^	1.15 × 10^−5^	1.15 × 10^−5^	1.15 × 10^−5^	1.15 × 10^−5^	1.15 × 10^−5^	1.15 × 10^−5^
	best	1.15 × 10^−5^	1.15 × 10^−5^	1.15 × 10^−5^	1.15 × 10^−5^	1.15 × 10^−5^	1.15 × 10^−5^	1.15 × 10^−5^	1.15 × 10^−5^	1.15 × 10^−5^	1.15 × 10^−5^	1.15 × 10^−5^	1.15 × 10^−5^	1.15 × 10^−5^
	worst	1.15 × 10^−5^	1.15 × 10^−5^	1.15 × 10^−5^	1.15 × 10^−5^	1.15 × 10^−5^	1.15 × 10^−5^	1.15 × 10^−5^	1.15 × 10^−5^	1.15 × 10^−5^	1.15 × 10^−5^	1.15 × 10^−5^	1.15 × 10^−5^	1.15 × 10^−5^
	std	2.06 × 10^−19^	2.23 × 10^−11^	2.56 × 10^−9^	5.02 × 10^−11^	1.25 × 10^−15^	2.4 × 10^−14^	6.12 × 10^−19^	1 × 10^−12^	3.75 × 10^−15^	7.88 × 10^−14^	2.01 × 10^−19^	6.24 × 10^−20^	2.77 × 10^−18^
	median	1.15 × 10^−5^	1.15 × 10^−5^	1.15 × 10^−5^	1.15 × 10^−5^	1.15 × 10^−5^	1.15 × 10^−5^	1.15 × 10^−5^	1.15 × 10^−5^	1.15 × 10^−5^	1.15 × 10^−5^	1.15 × 10^−5^	1.15 × 10^−5^	1.15 × 10^−5^
	rank	1	11	13	12	6	8	4	10	7	9	3	2	5
C11-F4	mean	0	0	0	0	0	0	0	0	0	0	0	0	0
	best	0	0	0	0	0	0	0	0	0	0	0	0	0
	worst	0	0	0	0	0	0	0	0	0	0	0	0	0
	std	0	0	0	0	0	0	0	0	0	0	0	0	0
	median	0	0	0	0	0	0	0	0	0	0	0	0	0
	rank	1	1	1	1	1	1	1	1	1	1	1	1	1
C11-F5	mean	−34.1274	−24.9831	−28.2273	−20.2162	−33.2934	−27.268	−27.7581	−27.1305	−31.6254	−11.175	−27.481	−9.04533	−9.89133
	best	−34.7494	−26.1071	−29.2962	−22.3239	−33.8791	−31.5884	−27.9121	−31.7824	−34.1584	−13.2553	−31.5869	−12.5467	−11.3064
	worst	−33.3862	−24.0719	−27.7732	−17.9006	−31.9949	−22.0431	−27.3472	−24.7046	−27.6716	−9.5842	−24.3626	−7.35878	−8.26201
	std	0.606664	0.954124	0.784459	2.5404	0.942943	4.250482	0.296382	3.570447	3.001446	1.69024	3.418314	2.641394	1.456586
	median	−34.1871	−24.8768	−27.9199	−20.3201	−33.6499	−27.7202	−27.8866	−26.0175	−32.3359	−10.9302	−26.9873	−8.13789	−9.99846
	rank	1	9	4	10	2	7	5	8	3	11	6	13	12
C11-F6	mean	−24.1119	−14.2181	−19.1303	−13.2402	−22.6478	−7.84618	−20.0368	−9.78463	−19.7197	−2.69281	−21.935	−3.54463	−4.43655
	best	−27.4298	−14.7657	−20.5764	−13.8753	−25.7881	−16.6588	−22.9903	−17.5337	−22.5047	−3.06335	−26.5427	−6.47064	−9.54426
	worst	−23.0059	−13.9749	−17.3535	−12.2206	−21.3642	−4.61296	−13.1388	−2.5693	−18.0809	−2.5693	−17.9788	−2.5693	−2.5693
	std	2.390663	0.397953	1.588785	0.820027	2.291442	6.365898	5.059561	8.726487	2.281691	0.266989	3.995435	2.108302	3.688901
	median	−23.0059	−14.0658	−19.2956	−13.4325	−21.7195	−5.05648	−22.009	−9.51776	−19.1466	−2.5693	−21.6092	−2.5693	−2.81632
	rank	1	7	6	8	2	10	4	9	5	13	3	12	11
C11-F7	mean	0.860699	1.588929	1.274321	1.895534	0.928052	1.291753	1.723324	0.8806	1.06276	1.698963	1.074534	1.117448	1.72023
	best	0.582266	1.522831	1.135433	1.662115	0.753267	1.11618	1.60924	0.82157	0.819432	1.514974	0.877437	0.835816	1.331785
	worst	1.025027	1.700462	1.415644	2.080803	1.011016	1.650959	1.89666	0.95286	1.28323	1.841822	1.270257	1.353691	1.92319
	std	0.217481	0.085626	0.166357	0.187391	0.128277	0.262758	0.132348	0.068143	0.205983	0.153914	0.191356	0.287067	0.290068
	median	0.91775	1.566211	1.273103	1.919609	0.973964	1.199937	1.693697	0.873984	1.074189	1.719527	1.07522	1.140142	1.812972
	rank	1	9	7	13	3	8	12	2	4	10	5	6	11
C11-F8	mean	220	283.7103	240.076	323.3338	222.3985	256.5766	265.1711	223.9974	227.1954	223.9974	245.8376	464.6706	222.4429
	best	220	257.7358	223.5533	283.1596	220	220	244.7841	220	220	220	220	247.5379	220
	worst	220	317.7863	256.5988	367.1058	224.7969	351.9156	310.3422	235.9898	234.3908	235.9898	291.9539	563.2385	229.7715
	std	0	28.41633	15.36966	37.21541	2.993307	69.08559	32.80142	8.640932	8.97992	8.640932	36.87853	161.4928	5.28057
	median	220	279.6596	240.076	321.535	222.3985	227.1954	252.779	220	227.1954	220	235.6982	523.9529	220
	rank	1	10	6	11	2	8	9	4	5	4	7	12	3
C11-F9	mean	8789.286	547,069.9	371,511.9	1,042,447	19,988.23	65,162.08	367,913.7	131,066	42,386.12	401,241.9	808,215.2	1,062,531	1,906,799
	best	5457.674	365,873.9	328,486.9	680,930.6	10,949.51	46,760.89	203,657	74,302.37	18,223.63	331,972.8	691,779.1	852,885.8	1,827,490
	worst	14,042.29	628,479.1	399,868.7	1,222,895	28,267.97	82,676.68	623,167.9	198,505.7	73,888.55	514,880.9	870,022.6	1,301,526	2,018,443
	std	3999.103	133,854.2	33,856.02	265,605.1	8314.79	16,558.29	206,695.1	55,384.58	25,423.37	87,013.21	85,744.4	259,125.6	101,650
	median	7828.591	596,963.3	378,846	1,132,982	20,367.72	65,605.38	322,415	125,728	38,716.15	379,057	835,529.5	1,047,857	1,890,632
	rank	1	9	7	11	2	4	6	5	3	8	10	12	13
C11-F10	mean	−21.4889	−14.12	−17.0125	−12.4613	−19.0763	−14.5312	−13.0442	−14.8369	−14.2509	−11.4892	−13.3191	−11.587	−11.2973
	best	−21.8299	−15.3076	−17.2046	−12.8483	−19.4648	−18.9145	−13.6865	−21.1931	−14.7348	−11.5875	−13.831	−11.6414	−11.351
	worst	−20.7878	−13.528	−16.6264	−12.2013	−18.6742	−12.2245	−12.5764	−11.6609	−13.1159	−11.3805	−12.5594	−11.5289	−11.207
	std	0.512709	0.876051	0.289133	0.307505	0.431975	3.257029	0.502411	4.650939	0.829736	0.095696	0.674467	0.049723	0.067749
	median	−21.669	−13.8221	−17.1095	−12.3978	−19.083	−13.4929	−12.9569	−13.2468	−14.5765	−11.4944	−13.4429	−11.5888	−11.3155
	rank	1	7	3	10	2	5	9	4	6	12	8	11	13
C11-F11	mean	571,712.3	5,699,003	982,575	8,695,685	1,637,333	5,838,576	1,202,495	1,293,544	3,768,573	5,118,129	1,394,379	5,128,987	6,013,014
	best	260,837.9	5,433,021	762,118.9	8,399,152	1,523,824	4,860,529	1,092,833	614,398.3	3,577,942	5,083,410	1,249,502	5,105,127	5,964,397
	worst	828,560.9	6,062,259	1,164,658	8,886,901	1,774,658	7,058,743	1,366,333	2,688,299	4,113,168	5,146,813	1,569,798	5,154,249	6,081,889
	std	268,296.7	317,615.3	189,801.9	225,029.1	132,340.1	981,647.8	127,052.8	1,018,233	255,889.5	29,963.32	142,773.7	27,163.81	54,586.44
	median	598,725.2	5,650,367	1,001,762	8,748,344	1,625,424	5,717,516	1,175,408	935,740.1	3,691,590	5,121,145	1,379,107	5,128,286	6,002,886
	rank	1	10	2	13	6	11	3	4	7	8	5	9	12
C11-F12	mean	1,199,805	8,247,720	3332149	12996285	1273063	4953266	5722934	1324897	1419579	14067521	5,698,270	2,291,644	14,225,236
	best	1,155,937	7,907,266	3,230,675	12,072,526	1,198,236	4,690,112	5,313,383	1,176,929	1,258,103	13,243,726	5,415,337	2,125,889	14,099,206
	worst	1,249,353	8,550,516	3,399,703	13,809,129	1,351,682	5,093,366	5,926,822	1,465,198	1,556,340	14,706,404	5,901,330	2,495,183	14,354,468
	std	48,490.42	288,426.6	79,806.35	769,838.2	72,755.16	202,184.7	305,498.4	127,369.5	133,721.5	663,071.6	226,167.1	164,645.1	112,791.6
	median	1,196,965	8,266,549	3,349,110	13,051,743	1,271,167	5,014,794	5,825,766	1,328,729	1,431,937	14,159,977	5,738,206	2,272,753	14,223,634
	rank	1	10	6	11	2	7	9	3	4	12	8	5	13
C11-F13	mean	15,444.2	15,849.66	15,447.92	16,293.63	15,462.8	15,489.3	15,533.71	15,506.67	15,500.01	15,923.76	126,162.5	15,489.93	29,722.15
	best	15,444.19	15,667.75	15,446.94	15,885.5	15,460.54	15,479.58	15,490.86	15,486.82	15,493.22	15,623.98	91,287.79	15,473.04	15,460.14
	worst	15,444.21	16,290.4	15,449.01	17,307.6	15,466.72	15,501.67	15,591.47	15,544.43	15,511.76	16,476.01	173,464.7	15,525.75	72,163.25
	std	0.009348	320.6504	0.938979	736.6177	2.959633	11.80676	50.59361	28.86293	8.881369	416.8059	39,987.59	26.08554	30,580.59
	median	15,444.2	15,720.26	15,447.87	15,990.71	15,461.98	15,487.98	15,526.25	15,497.71	15,497.53	15,797.52	119,948.7	15,480.47	15,632.6
	rank	1	9	2	11	3	4	8	7	6	10	13	5	12
C11-F14	mean	18,295.35	110,685.3	18,515.1	225,080.2	18,601.83	19,507.73	19,207.97	19,397.93	19,214.78	305,234.2	19,077.49	19,109.43	19,096.92
	best	18,241.58	84,287.1	18,400.49	165,852.5	18,517.66	19,256.18	19,055.73	19,297.38	19,069.82	30,025.79	18,794.25	18,949.03	18,818.17
	worst	18,388.08	154,677.8	18,613.52	324,221.2	18,678.45	20,044.58	19,324.43	19,476.34	19,395.21	588,832.6	19,282.27	19,254.99	19,389.24
	std	73.62303	34,029.61	108.1547	76,671.27	74.21487	390.7942	134.2957	82.00688	155.2719	289,952.1	228.9053	135.3985	252.3024
	median	18,275.87	101,888	18,523.19	205,123.5	18,605.6	19,365.08	19,225.86	19,409	19,197.05	301,039.2	19,116.72	19,116.85	19,090.13
	rank	1	11	2	12	3	10	7	9	8	13	4	6	5
C11-F15	mean	32,883.58	891,992.1	106,461.7	1,880,192	32,947.15	54,153.9	215,191.7	33,096.01	33,074.28	15,137,368	294,860.9	33,280.57	7,790,850
	best	32,782.17	368,009.3	42,983.25	786,645	32,868.23	33,046.02	33,005.18	33,011.17	33,040.28	3,172,284	261,029.5	33,272.8	3,546,456
	worst	32,956.46	2,242,044	177,487.4	4,907,029	33,017.52	117,203.6	307,751.1	33,154.09	33,139.62	22,572,975	318,021.3	33,293.28	13,351,586
	std	79.12175	976,285.5	78,132.6	2,184,347	66.11049	45,429.94	134,057.9	67.98468	50.6283	9,534,343	28,655.01	9.676778	4,859,075
	median	32,897.86	478,957.5	102,688	913,547.5	32,951.42	33,182.97	260,005.3	33,109.38	33,058.6	17,402,107	300,196.3	33,278.1	7,132,679
	rank	1	10	7	11	2	6	8	4	3	13	9	5	12
C11-F16	mean	133,550	930,219.4	135,146.9	1,915,857	137,581.4	144,911.9	142,003.9	141,654.9	145,644.1	87,266,512	18,380,211	78,107,911	74,996,694
	best	131,374.2	286,073.5	133,610	467,490	135,495.9	142,214	136,296.5	133,236.5	143,189	85,038,927	9,336,639	64,610,606	60,613,719
	worst	136,310.8	2,194,266	135,733	4,757,764	141,249.3	146,800.8	147,282.8	150,243.3	151,126.3	89,779,020	33,251,973	93,336,289	95,925,797
	std	2459.812	927,612	1111.105	2,085,461	2774.227	2428.678	4954.729	7708.969	3994.098	2,147,100	11,176,659	13,382,204	16,212,147
	median	133,257.5	620,269	135,622.3	1,219,088	136,790.1	145,316.4	142,218.1	141,570	144,130.5	87,124,050	15,466,115	77,242,375	71,723,630
	rank	1	8	2	9	3	6	5	4	7	13	10	12	11
C11-F17	mean	1,926,615	8.8 × 10^9^	2.27 × 10^9^	1.52 × 10^10^	2,284,236	1.26 × 10^9^	9.52 × 10^9^	3,090,266	2,999,477	2.19 × 10^10^	1.1 × 10^10^	2.04 × 10^10^	2.15 × 10^10^
	best	1,916,953	7.5 × 10^9^	2.06 × 10^9^	1.09 × 10^10^	1,956,608	1.04 × 10^9^	6.79 × 10^9^	2,290,263	2,035,918	2.11 × 10^10^	9.69 × 10^9^	1.8 × 10^10^	2.01 × 10^10^
	worst	1,942,685	9.75 × 10^9^	2.49 × 10^9^	1.86 × 10^10^	2,888,986	1.44 × 10^9^	1.27 × 10^10^	3,709,332	4,826,319	2.29 × 10^10^	1.17 × 10^10^	2.36 × 10^10^	2.42 × 10^10^
	std	12,342.79	1.08 × 10^9^	2.01 × 10^8^	3.56 × 10^9^	451,894.6	2.23 × 10^8^	2.67 × 10^9^	707,895.2	1,358,575	7.99 × 10^8^	9.7 × 10^8^	2.73 × 10^9^	2.05 × 10^9^
	median	1,923,412	8.97 × 10^9^	2.27 × 10^9^	1.57 × 10^10^	2,145,674	1.28 × 10^9^	9.31 × 10^9^	3,180,735	2,567,834	2.18 × 10^10^	1.13 × 10^10^	2.01 × 10^10^	2.08 × 10^10^
	rank	1	7	6	10	2	5	8	4	3	13	9	11	12
C11-F18	mean	942,057.5	53,992,864	6,452,285	1.16 × 10^8^	971,200.5	2,029,797	9,420,999	987,267	1029,486	30,449,642	10,940,996	1.32 × 10^8^	1.12 × 10^8^
	best	938,416.2	37,139,709	3,886,612	80,284,910	949,566.1	1,777,412	4,062,802	963,557.9	966,544.1	24,137,837	8,169,164	1.11 × 10^8^	1.08 × 10^8^
	worst	944,706.9	61,413,964	11,054,310	1.33 × 10^8^	1,028,421	2,366,184	16,526,008	998,198.6	1,195,849	32,937,033	13,797,505	1.47 × 10^8^	1.17 × 10^8^
	std	2852.546	12,286,170	3,607,658	26,529,357	41,362.17	306,787.1	5,687,958	17,303.95	120,120.5	4,566,634	2,717,842	17344620	3652762
	median	942,553.5	58,708,890	5,434,109	1.26 × 10^8^	953,407.4	1,987,796	8,547,593	993,655.7	977,776.1	32,361,850	10,898,658	1.36 × 10^8^	1.12 × 10^8^
	rank	1	10	6	12	2	5	7	3	4	9	8	13	11
C11-F19	mean	1,025,341	53,148,463	6,553,177	1.14 × 10^8^	1,135,759	2,437,008	10,049,581	1,468,377	1,356,595	34,957,413	6,171,131	1.7 × 10^8^	1.13 × 10^8^
	best	967,927.7	45,352,373	5,986,725	98,336,201	1,066,369	2,201,050	2,039,611	1,125,232	1,227,679	24,485,310	2,364,740	1.54 × 10^8^	1.1 × 10^8^
	worst	1,167,142	67,568,653	7,933,742	1.43 × 10^8^	1,290,586	2,872,382	18,188,632	1,941,594	1,537,473	43,599,463	8,096,958	1.96 × 10^8^	1.16 × 10^8^
	std	102,492.2	10,833,756	1,001,413	22,546,262	112,531.9	322,100.6	8,216,956	369,271.1	140,621.9	8,948,137	2,812,157	19,783,157	2,737,120
	median	983,146.6	49,836,412	6,146,121	1.07 × 10^8^	1,093,040	2,337,301	9,985,041	1,403,340	1,330,614	35,872,439	7,111,412	1.64 × 10^8^	1.13 × 10^8^
	rank	1	10	7	12	2	5	8	4	3	9	6	13	11
C11-F20	mean	941,250.4	56,509,020	5,801,655	1.23 × 10^8^	959,996	1,810,298	7,164,791	972,234.1	997,487.3	33,954,979	14,026,422	1.56 × 10^8^	1.13 × 10^8^
	best	936,143.2	49,719,897	5,117,171	1.08 × 10^8^	956,898.3	1,629,827	6,751,421	962,492.3	976,961.7	33,210,844	9,322,495	1.43 × 10^8^	1.08 × 10^8^
	worst	946,866.6	66,914,630	6,533,334	1.46 × 10^8^	961,914.3	2,110,594	7,716,489	983,286	1,013,457	34,759,801	21,703,055	1.7 × 10^8^	1.17 × 10^8^
	std	5155.253	7,919,081	635,291	17,773,474	2340.981	246,729.1	445,895.7	9846.595	16,998.67	696,370.4	5,847,652	16,191,798	4,388,670
	median	940,995.9	54,700,777	5778,058	1.19 × 10^8^	960,585.7	1,750,387	7,095,627	971,579.1	999,765.5	33,924,636	12,540,070	1.56 × 10^8^	1.14 × 10^8^
	rank	1	10	6	12	2	5	7	3	4	9	8	13	11
C11-F21	mean	12.71443	49.5692	21.48233	75.31365	15.87735	29.51655	38.32132	27.27063	22.20321	99.10431	40.19808	104.0484	100.9755
	best	9.974206	40.87478	20.10833	56.13583	13.68984	26.19513	35.12125	24.26861	20.4207	47.72372	35.44736	89.98702	57.96669
	worst	14.97499	58.87718	23.31862	94.46155	18.16644	30.99758	42.3	30.29163	24.54933	145.8555	43.09654	115.6756	123.1913
	std	2.480858	8.382327	1.480962	18.29105	2.24619	2.4322	3.426502	3.672225	1.98522	43.45024	3.685619	13.69468	32.80485
	median	12.95425	49.26243	21.25119	75.32861	15.82657	30.43674	37.932	27.26113	21.92141	101.419	41.12421	105.2655	111.372
	rank	1	9	3	10	2	6	7	5	4	11	8	13	12
C11-F22	mean	16.12513	46.26618	27.24332	62.63881	19.02841	31.85176	45.79438	32.0051	24.83497	101.0649	46.14521	105.026	91.21431
	best	11.50133	40.28398	21.98339	45.5216	16.10377	27.78821	39.51137	24.57618	23.84349	65.45846	38.64725	88.0089	90.18309
	worst	19.55286	51.79321	32.49921	72.15042	21.23028	34.41746	50.53472	37.07604	25.60263	119.7263	54.87466	116.0237	92.84607
	std	4.316441	5.332108	5.373903	12.72094	2.646729	3.089736	5.385079	5.98562	0.850828	26.35509	7.226255	13.61933	1.242127
	median	16.72317	46.49376	27.24535	66.44161	19.3898	32.6007	46.56572	33.18408	24.94688	109.5374	45.52947	108.0357	90.91404
	rank	1	9	4	10	2	5	7	6	3	12	8	13	11
Sum rank		22	191	109	231	55	146	145	118	97	222	157	198	224
Mean rank		1.00	8.68	4.95	10.5	2.50	6.64	6.59	5.36	4.41	10.1	7.14	9.00	10.2
Total rank		1	2	12	4	13	3	11	9	6	7	10	5	8
Wilcoxon: *p*-value			1.71 × 10^−15^	9.77 × 10^−15^	1.71 × 10^−15^	7.10 × 10^−15^	3.66 × 10^−15^	1.71 × 10^−15^	3.99 × 10^−12^	7.10 × 10^−15^	5.36 × 10^−15^	8.52 × 10^−15^	2.54 × 10^−15^	5.36 × 10^−15^

**Table 8:** Performance of optimization algorithms on the pressure vessel design problem.

Algorithm	Optimum Variables				Optimum Cost
	*T_s_*	*T_h_*	*R*	*L*	
KOA	0.7780271	0.3845792	40.312284	200	5882.8955
WSO	0.7780271	0.3845792	40.312284	200	5882.9013
AVOA	0.7780314	0.3845813	40.312509	199.99686	5882.9088
RSA	1.2659852	0.683916	63.993566	22.16777	8079.2663
MPA	0.7780271	0.3845792	40.312284	200	5882.9013
TSA	0.77975	0.38603	40.399151	200	5913.8806
WOA	0.9342363	0.4623891	47.238081	122.5792	6336.8911
MVO	0.8440256	0.4218218	43.712275	157.7639	6024.4345
GWO	0.7785336	0.3860227	40.322047	199.95837	5891.4545
TLBO	1.6957318	0.4977642	48.952657	111.82372	11,645.486
GSA	1.189919	1.2892052	44.756424	189.21969	13,022.865
PSO	1.6814562	0.6637242	67.02456	24.219082	10,699.118
GA	1.5133659	0.8511325	61.306853	52.513848	11,777.624

**Table 9:** Statistical results of optimization algorithms on the pressure vessel design problem.

Algorithm	Mean	Best	Worst	Std	Median	Rank
KOA	5882.8955	5882.8955	5882.8955	1.87 × 10^−12^	5882.8955	1
WSO	5892.6429	5882.9013	5979.0155	26.000485	5882.9017	3
AVOA	6276.8326	5882.9088	7244.3289	412.32817	6075.7427	5
RSA	13,520.395	8079.2663	22,393.029	3659.2762	12,342.889	9
MPA	5882.9013	5882.9013	5882.9013	4.31 × 10^−6^	5882.9013	2
TSA	6337.2069	5913.8806	7129.7183	389.86033	6187.9875	6
WOA	8358.7251	6336.8911	13,983.562	1968.1481	7868.4088	8
MVO	6626.21	6024.4345	7249.1095	374.8357	6689.5036	7
GWO	6034.402	5891.4545	6805.1241	280.12773	5901.2126	4
TLBO	32,084.076	11,645.486	69,575.146	16,143.602	28,224.957	12
GSA	23,155.786	13,022.865	36,568.394	7853.9605	22,204.189	10
PSO	33,739.014	10,699.118	58,342.053	15,113.442	37,275.068	13
GA	28,754.207	11,777.624	52,278.822	12,671.826	25,388.128	11

**Table 10:** Performance of optimization algorithms on the speed reducer design problem.

Algorithm	Optimum Variables							Optimum Cost
	b	*M*	*p*	*l*_1_	*l*_2_	*d*_1_	*d*_2_	
KOA	3.5	0.7	17	7.3	7.8	3.3502147	5.2866832	2996.3482
WSO	3.5000005	0.7	17	7.3000102	7.8000004	3.3502148	5.2866833	2996.3483
AVOA	3.5	0.7	17	7.3000008	7.8	3.3502147	5.2866832	2996.3482
RSA	3.5950209	0.7	17	8.2502092	8.2751046	3.3558321	5.4893788	3188.6002
MPA	3.5	0.7	17	7.3	7.8	3.3502147	5.2866832	2996.3482
TSA	3.5132973	0.7	17	7.3	8.2751046	3.3505506	5.2903255	3014.418
WOA	3.5901774	0.7	17	7.3	8.0158051	3.361964	5.286758	3039.5462
MVO	3.5023215	0.7	17	7.3	8.0773644	3.3701939	5.2868879	3008.6019
GWO	3.5006611	0.7	17	7.3053023	7.8	3.3643722	5.2888758	3001.6737
TLBO	3.5578323	0.704121	26.612082	8.1261628	8.1558799	3.6731217	5.3409871	5340.6121
GSA	3.5236186	0.7028384	17.380563	7.8366336	7.8923823	3.4105869	5.389006	3175.0876
PSO	3.5084369	0.7000742	18.129553	7.4021022	7.870135	3.603038	5.3457978	3312.0108
GA	3.5804277	0.7057375	17.839	7.7562744	7.8575718	3.7124313	5.3481792	3360

**Table 11:** Statistical results of optimization algorithms on the speed reducer design problem.

Algorithm	Mean	Best	Worst	Std	Median	Rank
KOA	2996.3482	2996.3482	2996.3482	9.33 × 10^−13^	2996.3482	1
WSO	2996.6405	2996.3483	2998.8751	0.6029466	2996.3649	3
AVOA	3000.9954	2996.3482	3011.5309	4.0907833	3000.8928	4
RSA	3285.4608	3188.6002	3345.574	59.295486	3300.799	9
MPA	2996.3482	2996.3482	2996.3482	3.28 × 10^−6^	2996.3482	2
TSA	3033.2399	3014.418	3047.3957	10.453483	3035.0831	7
WOA	3154.8097	3039.5462	3458.9999	109.58738	3120.44	8
MVO	3030.8587	3008.6019	3072.4627	13.667672	3031.3121	6
GWO	3004.8775	3001.6737	3011.027	2.5848683	3004.3436	5
TLBO	7.171 × 10^13^	5340.6121	5.19 × 10^14^	1.193 × 10^14^	2.808 × 10^13^	12
GSA	3468.8298	3175.0876	4109.0755	270.3186	3335.169	10
PSO	1.058 × 10^14^	3312.0108	5.361 × 10^14^	1.278 × 10^14^	7.569 × 10^13^	13
GA	5.095 × 10^13^	3357.7007	3.289 × 10^14^	8.026 × 10^13^	2.041 × 10^13^	11

**Table 12:** Performance of optimization algorithms on the welded beam design problem.

Algorithm	Optimum Variables				Optimum Cost
	*h*	*l*	*t*	*b*	
KOA	0.2057296	3.4704887	9.0366239	0.2057296	1.7246798
WSO	0.2057296	3.4704887	9.0366239	0.2057296	1.7248523
AVOA	0.2049413	3.4875839	9.036514	0.2057347	1.7259523
RSA	0.1964182	3.5366405	9.9520327	0.2181668	1.9831072
MPA	0.2057296	3.4704887	9.0366239	0.2057296	1.7248523
TSA	0.2041488	3.4961387	9.0650317	0.2061694	1.7341191
WOA	0.2139726	3.3254365	8.9719001	0.2214638	1.8242648
MVO	0.2060012	3.4646369	9.0449319	0.2060655	1.7284719
GWO	0.2055878	3.4737417	9.0362284	0.2058009	1.7255441
TLBO	0.3185927	4.4505676	6.729429	0.4317775	3.0631667
GSA	0.2965218	2.6988989	7.3719858	0.3110575	2.0954226
PSO	0.3776166	3.4232855	7.2930935	0.5851578	4.0927486
GA	0.2248746	7.0193634	7.724663	0.3073695	2.7924716

**Table 13:** Statistical results of optimization algorithms on the welded beam design problem.

Algorithm	Mean	Best	Worst	Std	Median	Rank
KOA	1.7246798	1.7246798	1.7246798	2.28 × 10^−16^	1.7246798	1
WSO	1.7248527	1.7248523	1.724858	1.289 × 10^−6^	1.7248523	3
AVOA	1.7623182	1.7259523	1.8462594	0.0375758	1.7480047	7
RSA	2.1954892	1.9831072	2.5536739	0.1485183	2.1696915	8
MPA	1.7248523	1.7248523	1.7248523	3.46 × 10^−9^	1.7248523	2
TSA	1.7437046	1.7341191	1.7531762	0.0057759	1.7438038	6
WOA	2.3285011	1.8242648	4.1166131	0.6611968	2.0967293	9
MVO	1.7417197	1.7284719	1.7765724	0.0141747	1.7375259	5
GWO	1.7273254	1.7255441	1.7314921	0.0014042	1.7270728	4
TLBO	3.427 × 10^13^	3.0631667	3.307 × 10^14^	8.359 × 10^13^	5.8118858	12
GSA	2.4655242	2.0954226	2.7834667	0.1973298	2.4959644	10
PSO	4.726 × 10^13^	4.0927486	2.861 × 10^14^	9.026 × 10^13^	6.881908	13
GA	1.16 × 10^13^	2.7924716	1.255 × 10^14^	3.561 × 10^13^	5.7774686	11

**Table 14:** Performance of optimization algorithms on the tension/compression spring design problem.

Algorithm	Optimum Variables			Optimum Cost
	*d*	*D*	*P*	
KOA	0.0516891	0.3567177	11.288966	0.0126019
WSO	0.051687	0.3566687	11.291844	0.0126652
AVOA	0.0511766	0.3445208	12.043632	0.0126703
RSA	0.0500841	0.3128747	14.815225	0.0131729
MPA	0.0516908	0.3567595	11.286517	0.0126652
TSA	0.0509675	0.3395949	12.379923	0.0126825
WOA	0.0511504	0.3439031	12.084068	0.0126709
MVO	0.0500841	0.3188461	13.963843	0.0127523
GWO	0.0519643	0.363356	10.914486	0.0126708
TLBO	0.0682172	0.9079231	2.462505	0.0176238
GSA	0.0552141	0.4436774	7.7157776	0.0130859
PSO	0.0681323	0.9047167	2.462505	0.0175188
GA	0.0686985	0.9159566	2.462505	0.0180295

**Table 15:** Statistical results of optimization algorithms on the tension/compression spring design problem.

Algorithm	Mean	Best	Worst	Std	Median	Rank
KOA	0.0126019	0.0126019	0.0126019	6.88 × 10^−18^	0.0126019	1
WSO	0.0126766	0.0126652	0.0128288	3.645 × 10^−5^	0.0126657	3
AVOA	0.0133542	0.0126703	0.0141777	0.0005668	0.0132848	8
RSA	0.013256	0.0131729	0.0134024	7.054 × 10^−5^	0.0132346	6
MPA	0.0126652	0.0126652	0.0126652	2.90 × 10^−9^	0.0126652	2
TSA	0.0129674	0.0126825	0.0135406	0.0002456	0.0128926	5
WOA	0.0132825	0.0126709	0.0145297	0.0006143	0.013081	7
MVO	0.0165382	0.0127523	0.0179998	0.0016747	0.0174694	9
GWO	0.0127239	0.0126708	0.012951	5.622 × 10^−5^	0.0127214	4
TLBO	0.0181642	0.0176238	0.0187814	0.000364	0.0181192	10
GSA	0.0195368	0.0130859	0.0323907	0.0043308	0.0191036	11
PSO	2.127 × 10^13^	0.0175188	3.774 × 10^14^	8.445 × 10^13^	0.0175188	13
GA	1.661 × 10^12^	0.0180295	1.719 × 10^13^	4.961 × 10^12^	0.0257708	12

## Data Availability

Not applicable.

## Appendix A

The information of CEC 2017 test suite is determined in Table A1.

**Table A1:** Information of the CEC 2017 test functions.

		Functions	Fmin
Unimodal functions	C1	Shifted and Rotated Bent Cigar Function	100
	C2	Shifted and Rotated Sum of Different Power Function	200
	C3	Shifted and Rotated Zakharov Function	300
Simple multimodal functions	C4	Shifted and Rotated Rosenbrock’s Function	400
	C5	Shifted and Rotated Rastrigin’s Function	500
	C6	Shifted and Rotated Expanded Scaffer’s Function	600
	C7	Shifted and Rotated Lunacek Bi_Rastrigin Function	700
	C8	Shifted and Rotated Non-Continuous Rastrigin’s Function	800
	C9	Shifted and Rotated Levy Function	900
	C10	Shifted and Rotated Schwefel’s Function	1000
Hybrid functions	C11	Hybrid Function 1 (*N* = 3)	1100
	C12	Hybrid Function 2 (*N* = 3)	1200
	C13	Hybrid Function 3 (*N* = 3)	1300
	C14	Hybrid Function 4 (*N* = 4)	1400
	C15	Hybrid Function 5 (*N* = 4)	1500
	C16	Hybrid Function 6 (*N* = 4)	1600
	C17	Hybrid Function 6 (*N* = 5)	1700
	C18	Hybrid Function 6 (*N* = 5)	1800
	C19	Hybrid Function 6 (*N* = 5)	1900
	C20	Hybrid Function 6 (*N* = 6)	2000
Composition functions	C21	Composition Function 1 (*N* = 3)	2100
	C22	Composition Function 2 (*N* = 3)	2200
	C23	Composition Function 3 (*N* = 4)	2300
	C24	Composition Function 4 (*N* = 4)	2400
	C25	Composition Function 5 (*N* = 5)	2500
	C26	Composition Function 6 (*N* = 5)	2600
	C27	Composition Function 7 (*N* = 6)	2700
	C28	Composition Function 8 (*N* = 6)	2800
	C29	Composition Function 9 (*N* = 3)	2900
	C30	Composition Function 10 (*N* = 3)	3000
